# Supplementary material for: Establishment of DNA methylation during primate germ cell development
Source: Nat Commun. 2026 Apr 8;17:4983. doi: 10.1038/s41467-026-71405-z (PMC13237064; doi:10.1038/s41467-026-71405-z)

Establishment of DNA methylation during primate germ cell development

Supplementary Figure Legends

Supplementary Fig. 1. Genomic annotations and DNA methylation patterns along chromosomes in marmosets

The difference in average methylation levels between G7 (60%–80.1%) and G5 (40%–50%) is shown as the blue line (G7–G5), whereas the red line (G3–G1) represents the difference between G3 (20%–30%) and G1 (0%–10%). Mappability, determined by Bismap software with a 50-nt input length, indicates the uniqueness of bisulfite-converted genomic sequences. The average DNA methylation level of 3-month testis germ cells is depicted in black (labeled as 3M). scATAC-seq data of newborn (NB) and 3-month-old (TM) testes germ cells are included. DNA methylation and ATAC analyses for the 3-month-old marmoset were performed on the same sample.

Supplementary Fig. 2. Gene-structure-based annotation

(a) Definitions of gene structures, including intergenic, promoter, exon, intron, and gene flanking regions, are presented. When multiple variants were present, annotation was performed in order of priority as described in supplementary methods. (b) Gene structure-based classification of CpG islands, ATAC peaks, and retrotransposon sequences in marmosets (top) and cynomolgus monkeys (bottom). Source data are provided as a Source Data file.

Supplementary Fig. 3. Germ cell cluster selection in newborn testis scATAC-seq

Cluster 13 was identified as the germ cell cluster based on chromatin states of marker genes (GATA4: Sertoli cells, INSL3: Leydig cells, PIWIL4/DDX4/GTSF1: germ cells).

Supplementary Fig. 4. Germ cell cluster selection in 3-month testis ATAC-seq Cluster 13 was identified as the germ cell cluster for the 3-month testis.

Supplementary Fig. 5. Genomic annotations and DNA methylation patterns along chromosomes in cynomolgus monkeys

The difference in average methylation levels between G5 (68.2%–72.8%) and G3 (52.0%–59.8%) is

shown as the blue line (G5–G3), whereas the red line (G2–G1) represents the difference between G2 (28.4%–41.1%) and G1 (2.5%–4.8%). See the legend of Supplementary Fig. 1 for other information.

Supplementary Fig. 6. Loss of 5mC signal after long-term storage

Immunofluorescence analyses of a 1-year 5-month testis examined either 6 days (top) or 9 months (bottom) after sampling. Results from the same testis at 6 days post-sampling are shown in Fig. 7B. White arrows in DAPI/5mC images indicate nuclei lacking 5mC staining.

Supplementary Fig. 7. Genomic annotations and DNA methylation patterns along human chromosomes.

See the legend of Supplementary Fig. 1 for additional information.

Supplementary Fig. 8. FACS of germ cells

(a) Gating strategies for isolating germ cells from 7-month-old marmoset, 1-year-and-8-month-old cynomolgus monkey, and 1-year-and-5-month-old human testes are shown. Cells were selected using CD9 and CD90 signals (marmosets and cynomolgus monkeys) or the SSEA4 signal (humans), followed by FSC gating. (b) Immunofluorescence analysis of FACS-sorted cells to determine germ cell rates post-sorting (Supplementary Table 2).

Supplementary Methods

Sperm collection

Ejaculated sperm were collected via penile vibratory stimulation from a 12-year-old marmoset. Sperm were suspended in 600 μL TYH, preincubated in a 5% CO_2 incubator, and centrifuged at 500 *g* for 5 min at room temperature. After removing 550 μL of the supernatant, 500 μL of fresh TYH was added, and the sample was incubated for 30 min in 5% CO_2. Activated sperm were centrifuged, and the pellet was resuspended in 100 μL of TYH. Resuspended sperm were placed under 0.7 mL TYH in a 5 mL tube and incubated for 30 min in 5% CO_2. Swim-up sperm were collected, and sperm DNA was isolated using lysis solution [20 mM Tris–HCl (pH 8), 10 mM dithiothreitol, 150 mM NaCl, 10 mM EDTA (pH 8), 1.0% SDS, and 20 μg/mL proteinase K] at 50°C overnight. Phenol/Chloroform/Isoamyl alcohol (25:24:1) extraction and isopropanol precipitation were performed to purify DNA.

Annotation

NCBI *Callithrix jacchus* Annotation Release 105 (calJac4), NCBI *Macaca fascicularis* Annotation Release 101 (macfas5), and NCBI human (hg38) Refseq annotation (Refseq All) retrieved using UCSC table browser were used for gene structure-based annotation. Annotations (see Supplementary Fig. 2A for the definition of gene structure) were prioritized in the following order: promoter, exon, intron, gene flanking, and intergenic regions, considering all gene variants in the analyses. Promoters were defined as 501-bp regions (positions −250 to +250) surrounding the most upstream transcription start sites (position 0) across the gene variants. Intergenic regions were defined as genomic regions located more than 10 kbp away from genes. For retrotransposon analyses, coordinates of full-length LINE1 and Platy-1 elements were obtained from L1base2 (l1base.charite.de/l1base.php) and relevant literature[1], respectively. The calJac3 coordinates were converted to calJac4 using UCSC's Lift Genome Annotations tool. Major retrotransposon class coordinates (LTR, LINE, and SINE) were extracted from the UCSC repeat masker file. Promoters were categorized by CpG site counts in the 501-bp regions: low (0–12 CpG sites; 17,378 promoters), medium (13–34; 7,133), and high (35+; 7,887) for marmosets; low (0–12 CpG sites; 13,036 promoters), medium (13–34; 6,498), and high (35+; 7,023) for cynomolgus monkeys. CpG islands (unmasked CpGs) were sourced from the UCSC table browser. ATAC peaks were identified as outlined below. CpG site annotation used the first coordinate of the CpG site (C nucleotide on the top strand). Bedtools (version 2.31.0) was used to calculate the methylation rate of each annotation region.

The R package KaryoploteR was employed to visualize annotations and methylation levels across chromosomes[2]. To depict average annotation densities (genes and CpG islands) and DNA methylation levels, 500-kbp windows were used in *KaryoploteR*. For retrotransposon densities, GC content, % of N, and mappability, 100-kbp windows were applied. ATAC peaks were depicted by bars.

The Homer makeTagDirectory (-format bismark -minCounts 1) and annotatePeaks.pl (-CpG -ratio -size 10000 -hist 100) were used to calculate the methylation rate within 10k-bp around the center of CpG islands, TSS, and ATAC peak regions. For the random regions of the genome, 5000 random

loci were selected for each chromosome, and the methylation rates for the 10-kbp regions around these loci were calculated. Methylation rates were visualized by ggplot2.

Classifying genomic tiles based on the pattern of *de novo* DNA methylation

Genomes were segmented into 500-kbp tiles. For marmoset and human analyses, in each tile, the average methylation rate was calculated for every seven groups (Group 1-7). Differences in average methylation rates between Group 3 and Group 1 (G3–G1) and Group 7 and Group 5 (G7–G5) were calculated to classify tiles (x = (G3–G1) − (G7–G5)). Each tile was categorized as "fast" (marmosets: x > 2, 1414 loci; humans: x > 2, 2187 loci), "intermediate" (marmosets: 2 > = x > = −11, 2777 loci; humans: 2 > = x > = −11, 1871 loci), and "slow" (marmosets: −11 > x; 1428 loci, humans: −11 > x; 1859 loci). For cynomolgus monkeys, in each tile, the average methylation rate was calculated for every five group (Group 1-5). Based on the difference (x = (G2–G1) − (G5–G3)), each tile was classified into "fast" (x > 19.5; 1425 loci), "intermediate" (19.5 > = x > = 0; 3016 loci), and "slow" (0 > x; 1267 loci).

CH methylation analysis

CH methylation rates and motif enrichment for methylated dinucleotides and trinucleotides in marmoset and cynomolgus prospermatogonia were calculated using Bismark pipeline reports. bismark_methylation_extractor was conducted with the following options: -o $outputdir –CX --gzip -bedGraph -buffer_size 40G --cytosine_report --genome_folder $refGenome.

Single-cell RNA-seq data preprocessing and read mapping

Our custom scRNA-seq libraries[3] were designed for analysis using Cell Ranger. Before processing, Read1 and Read2 were swapped by renaming the files. Datasets were mapped to the common marmoset genome (calJac4) with Cell Ranger, using NCBI Callithrix jacchus Annotation Release 105 for genome annotation. UMI count data were analyzed with Seurat version 5.1.0. A count matrix was generated using the CreateSeuratObject function. Normalization, gene assignment for cell cycle scores

(S-score and G2/M-score), and scaling were performed. Parameters for PCA and clustering analyses included variable features = 4,000, PCA dimensions = 1:30, and clustering resolution = 0.5. For average gene expression levels, feature counts per cell were normalized by dividing by total counts, multiplying by 10,000, and applying a natural-log transformation using Seurat LogNormalize function. The group-wise average expression levels were then analyzed.

Correlation analysis of gene body DNA methylation and transcription

Gene bodies were defined using calJac4 annotations (n = 34,196 genes), excluding promoter regions (TSS ±2 kb). Mean CG methylation across each gene body was calculated per group (G1–G7) with bedtools intersect and combined with marmoset scRNA-seq data. Genes shorter than the defined promoter regions or lacking scBS-seq read coverage in a given group were excluded. Within each group, genes were evenly divided into three expression categories—“high,” “mid,” and “low” (~8,600 genes per group). Correlations with DNA methylation levels were subsequently assessed. Normalized log2 mean expression values across G1–G7 were high = 0.88, mid = 0.06, low = 0.00. Statistical differences were evaluated using a two-sided unpaired Wilcoxon test.

scATAC-seq library construction

Testicular cells (1×10^6) were transferred to a 1.5-mL microcentrifuge tube and centrifuged at 300 ×*g* for 5 min. After carefully removing the supernatant without disturbing the pellet, 200 µl of chilled lysis buffer [10 mM Tris-HCl (pH 7.4), 10 mM NaCl, 3 mM $MgCl_2$, 0.1% Tween-20, 0.1% NP-40, 0.01% digitonin, and 1% BSA] was added and gently mixed by pipetting five times. Then, cells were incubated for 30 sec. Lysed cells were treated with wash buffer [10 mM Tris-HCl (pH 7.4), 10 mM NaCl, 3 mM $MgCl_2$, 0.1% Tween-20, and 1% BSA] and mixed by gently inverting the tube five times. The cells were centrifuged at 300 ×*g* for 5 min, and the supernatant was removed. The pellets were resuspended in diluted Nuclei Buffer (10× Genomics, 2000207) and passed through a 40-µm filter to obtain a uniform single-cell suspension. Lysed cells were counted and examined under a microscope

to verify successful permeabilization. To recover 10,000 cells, 15,000 single cells were loaded into one channel of the Chromium Next GEM Chip H (10× Genomics, 1000162).

Library construction was carried out using the Chromium Next GEM Single Cell ATAC Library Kits v1.1 (10× Genomics, 1000176) following the User Guideline, Revision D (10× Genomics, CG000209). Sequencing was performed on a NovaSeq 6000, and the sequence reads were mapped to the calJac4 reference genome using Cell Ranger ATAC. Downstream analysis was conducted with the ArchR package.

scATAC-seq data processing

Single-cell ATAC-seq data were analyzed within a Docker container to ensure reproducibility (hattyoriiiiiii/single-cell-analysis:1.0.0). The calJac4 genome sequence and corresponding gene annotation files were downloaded to create a reference for Cell Ranger ATAC. A custom reference folder was generated using the “cellranger-atac mkref” command as per 10× Genomics guidelines. Single-cell ATAC-seq reads were processed with “cellranger-atac count” using default parameters (10× Genomics, v.2.0.0).

The fragment files from the “cellranger-atac count” output were analyzed using the ArchR package[4], which integrates chromVAR, Seurat, and MACS2. A custom BSgenome package for calJac4 was created following Bioconductor documentation. Metadata and configuration were defined in a seed file (BSgenome, Cjacchus, UCSC.calJac4-seed). To exclude doublets and low-quality cells, TSS enrichment and unique fragment counts were evaluated. Cells with TSS enrichment of >4 and unique fragments of >1,000 were retained, eliminating empty droplets. A total of 16,530 (newborn) and 11,137 (3-month-old) barcodes passed this initial filtering step, with newborn and 3-month libraries analyzed separately. Doublet removal was performed using the addDoubletScores() function (filterRatio = 1), yielding 13,798 (newborn) and 9,897 (3-month-old high-quality cells).

Dimensional reduction was performed using an iterative latent semantic indexing (LSI) approach on genome-wide 500-bp tiles to reduce experimental noise. Batch effects were corrected in LSI space using Harmony (v1.0). Clusters within the LSI subspace were identified using the Louvain algorithm

via the addCluster() function. To visualize the high-dimensional dataset in 2D, the addUMAP() function in ArchR was used.

To examine the relationship between chromatin accessibility and DNA methylation acquisition, genes were assigned to three categories: "fast" (11,767 genes), "intermediate" (7,019 genes), and "slow" (6,799 genes) according to the acquisition pattern of gene body methylation. To assign to three categories, we applied the same condition used for genomic tile classification. ATAC-seq signals of germ cells for each category were visualized using deepTools computeMatrix and plotProfile.

Annotation of clusters, peak identification, and pairwise test

Cluster annotation was performed for the newborn (Supplementary Fig. 3) and 3-month-old (Supplementary Fig. 4) samples based on visualized accessibility. Chromatin accessibility in each cluster was visualized by extracting reads from the BAM file of the cellranger output. Fragments derived from potential doublets (>40,000 unique fragments) were removed during extraction. Extracted fragments (bam files) were converted to signal tracks (bigwig file) and displayed in the genome browser to examine accessibility at representative marker genes.

Peaks in each cluster were identified using the pseudobulk method. The addGroupCoverages() and addReproduciblePeakSet() functions in ArchR were used with default settings (501-bp fixed-width peaks) and genomeSize = 2.9e9 for addReproduciblePeakSet(), which applies MACS2 for peak identification. A q-value cutoff of <0.1 was employed. Code availability: The R and shell scripts used in the ATAC analyses can be obtained from GitHub (https://github.com/Hattyoriiiiiii/scatac-caljac-watanabe).

References

1 Konkel, M. K. *et al.* Discovery of a new repeat family in the Callithrix jacchus genome. *Genome Res* **26**, 649-659 (2016). https://doi.org/10.1101/gr.199075.115

2 Gel, B. & Serra, E. karyoploteR: an R/Bioconductor package to plot customizable genomes

displaying arbitrary data. *Bioinformatics* **33**, 3088-3090 (2017). https://doi.org/10.1093/bioinformatics/btx346

3 Kubiura-Ichimaru, M. *et al.* mRNA-based generation of marmoset PGCLCs capable of differentiation into gonocyte-like cells. *Stem Cell Reports* **18**, 1987-2002 (2023). https://doi.org/10.1016/j.stemcr.2023.08.006

4 Granja, J. M. *et al.* ArchR is a scalable software package for integrative single-cell chromatin accessibility analysis. *Nat Genet* **53**, 403-411 (2021). https://doi.org/10.1038/s41588-021-00790-6

Supplementary Figure. 1

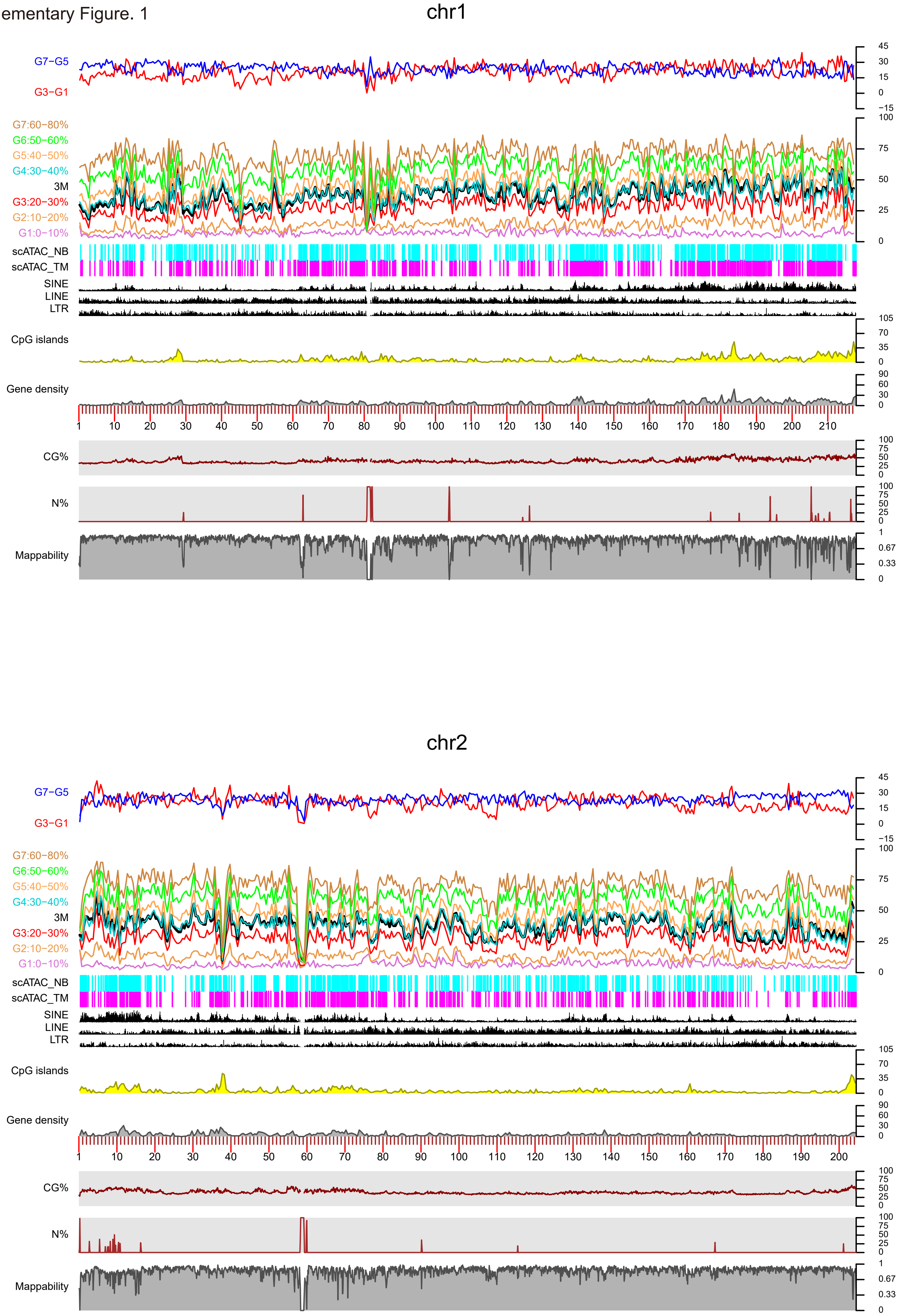

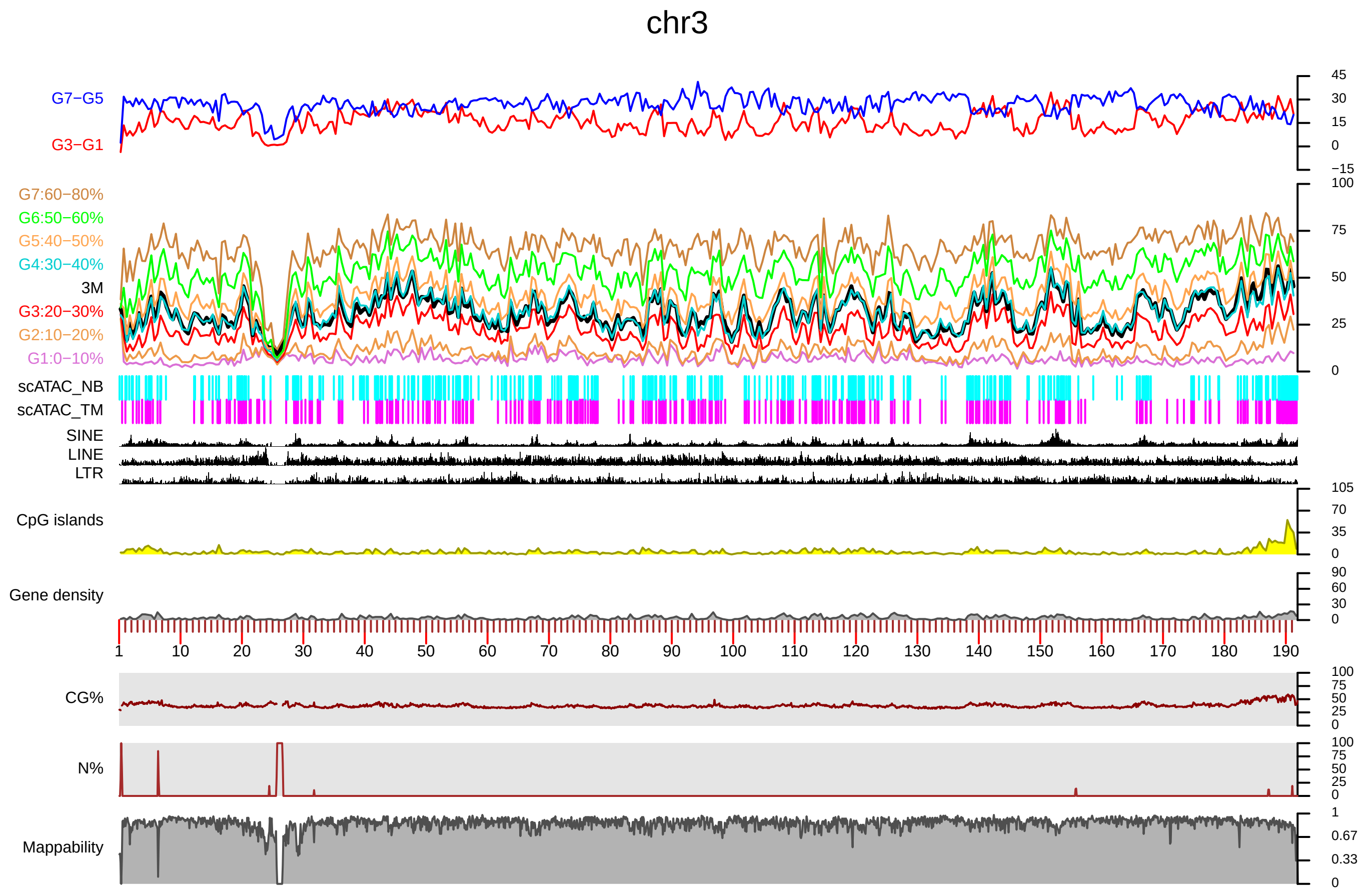
chr3
G7−G5
G3−G1
G7:60−80%
G6:50−60%
G5:40−50%
G4:30−40%
3M
G3:20−30%
G2:10−20%
G1:0−10%
scATAC_NB
scATAC_TM
SINE
LINE
LTR
CpG islands
Gene density
CG%
N%
Mappability

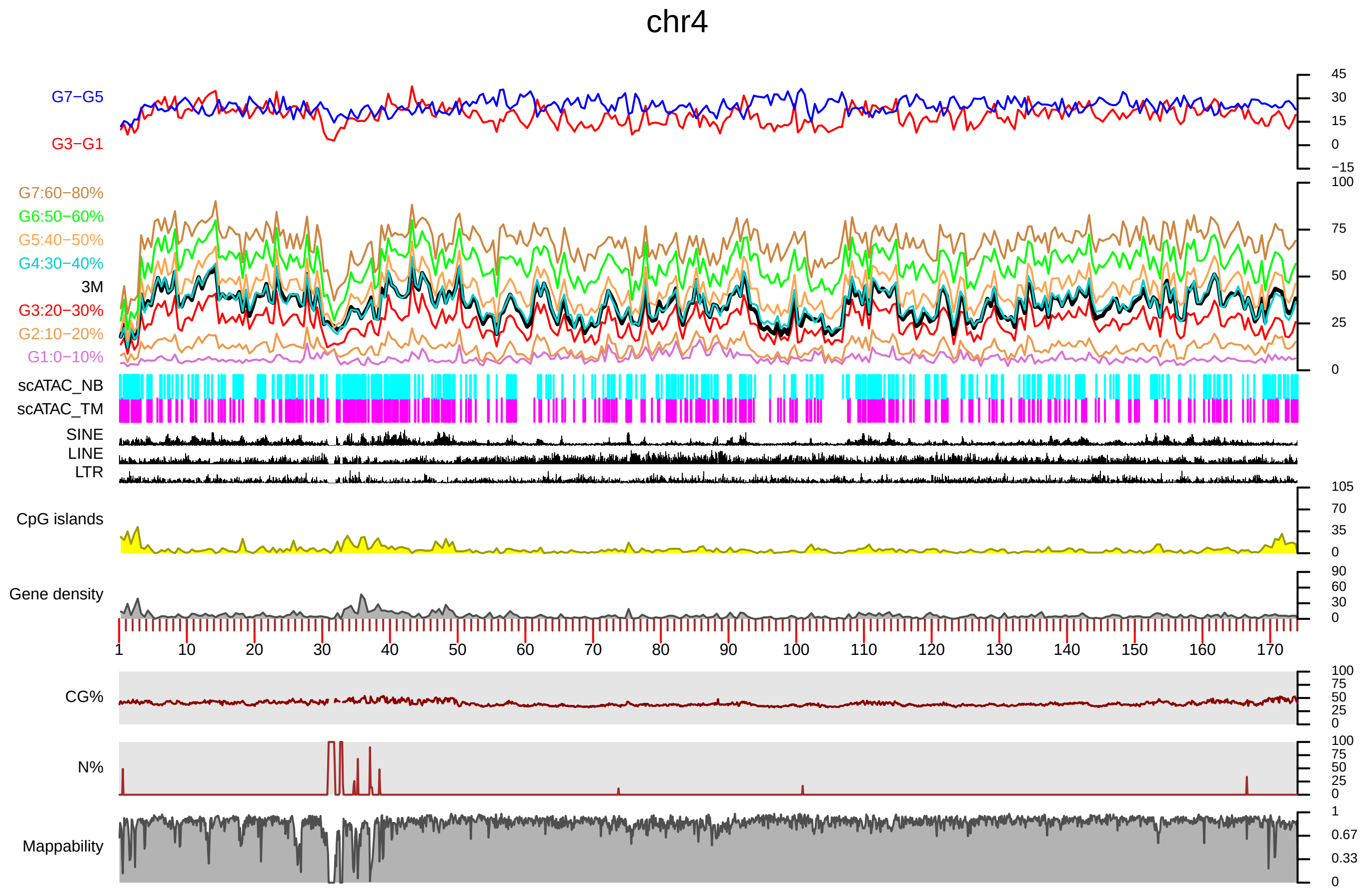
chr4
G7−G5
G3−G1
G7:60−80%
G6:50−60%
G5:40−50%
G4:30−40%
3M
G3:20−30%
G2:10−20%
G1:0−10%
scATAC_NB
scATAC_TM
SINE
LINE
LTR
CpG islands
Gene density
CG%
N%
Mappability

chr5

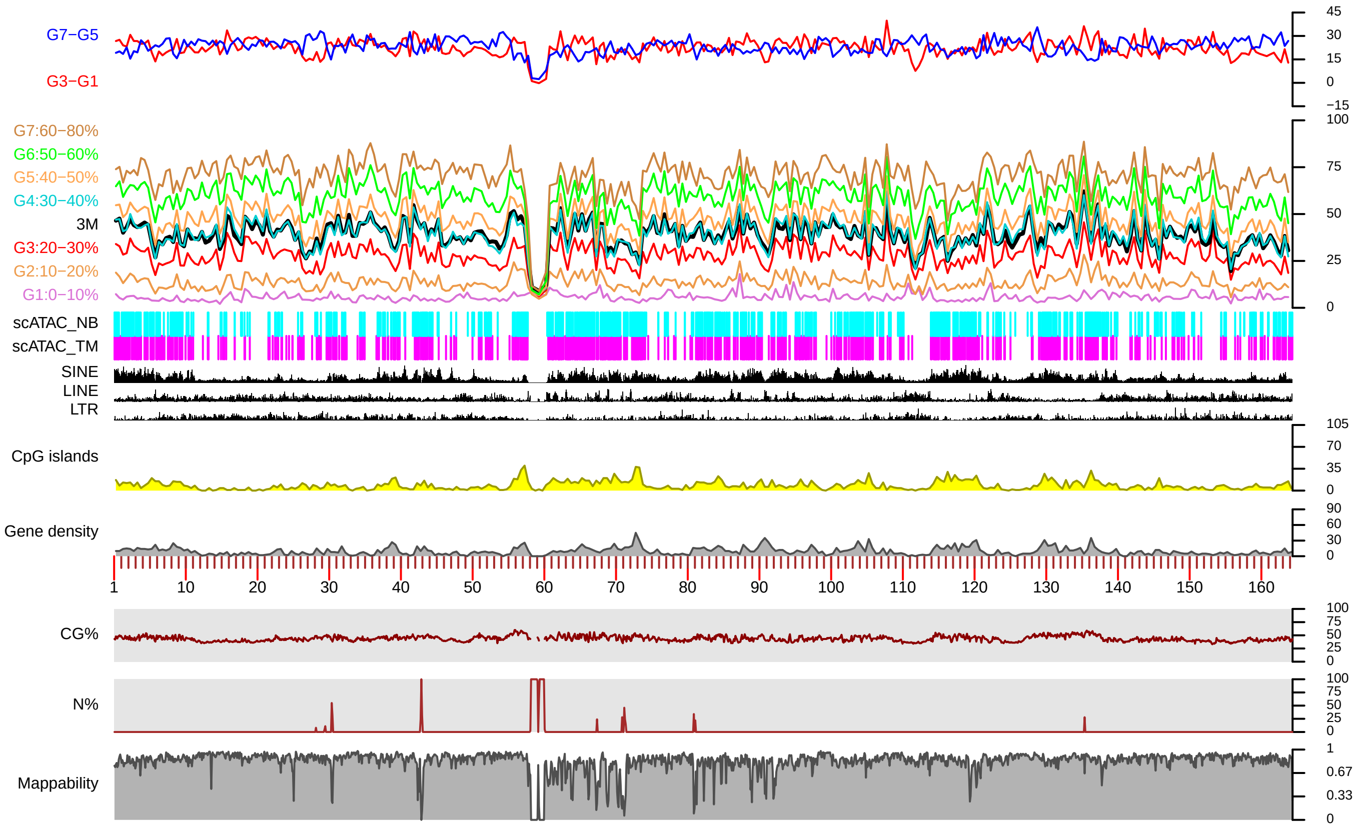

G7−G5
G3−G1
G7:60−80%
G6:50−60%
G5:40−50%
G4:30−40%
3M
G3:20−30%
G2:10−20%
G1:0−10%
scATAC_NB
scATAC_TM
SINE
LINE
LTR
CpG islands
Gene density
CG%
N%
Mappability

chr6

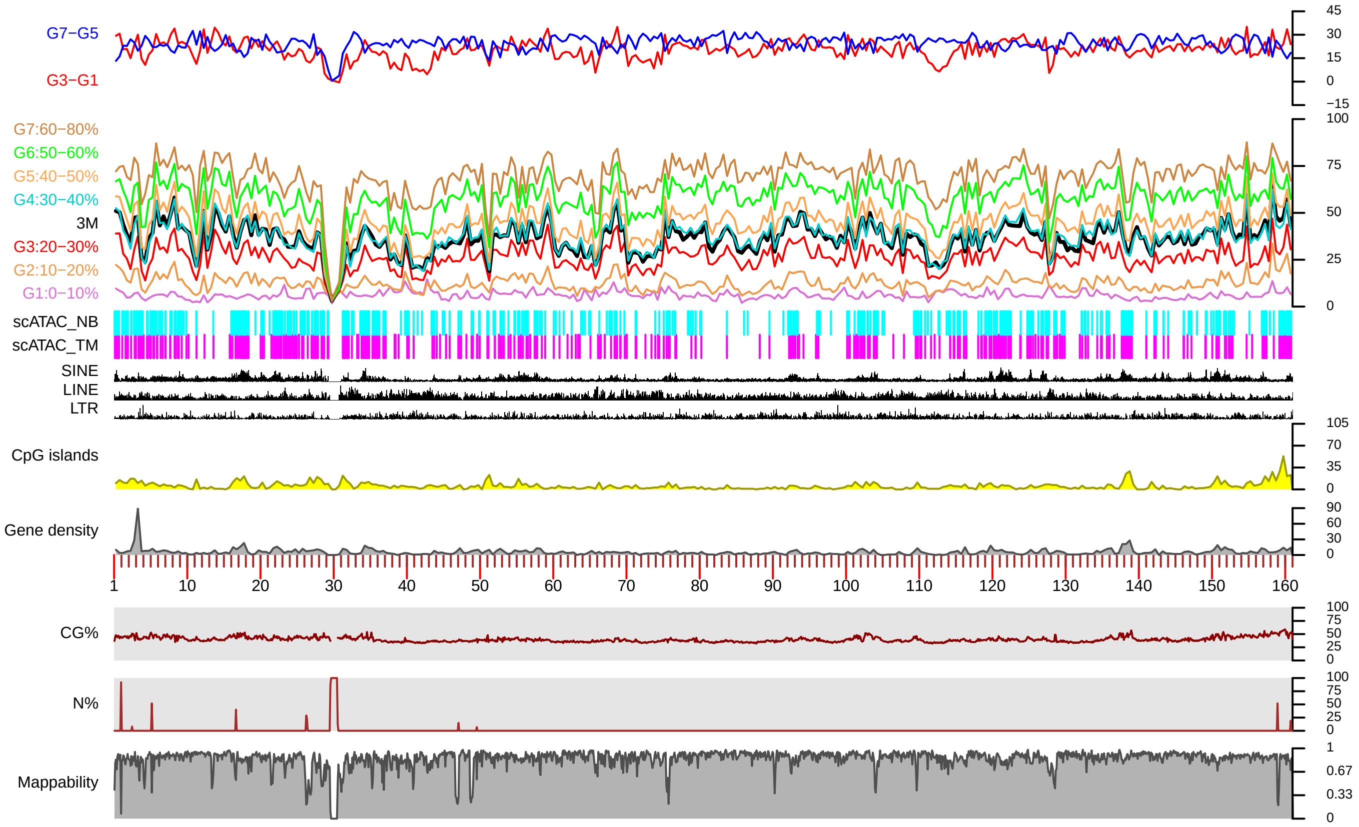

G7−G5
G3−G1
G7:60−80%
G6:50−60%
G5:40−50%
G4:30−40%
3M
G3:20−30%
G2:10−20%
G1:0−10%
scATAC_NB
scATAC_TM
SINE
LINE
LTR
CpG islands
Gene density
CG%
N%
Mappability

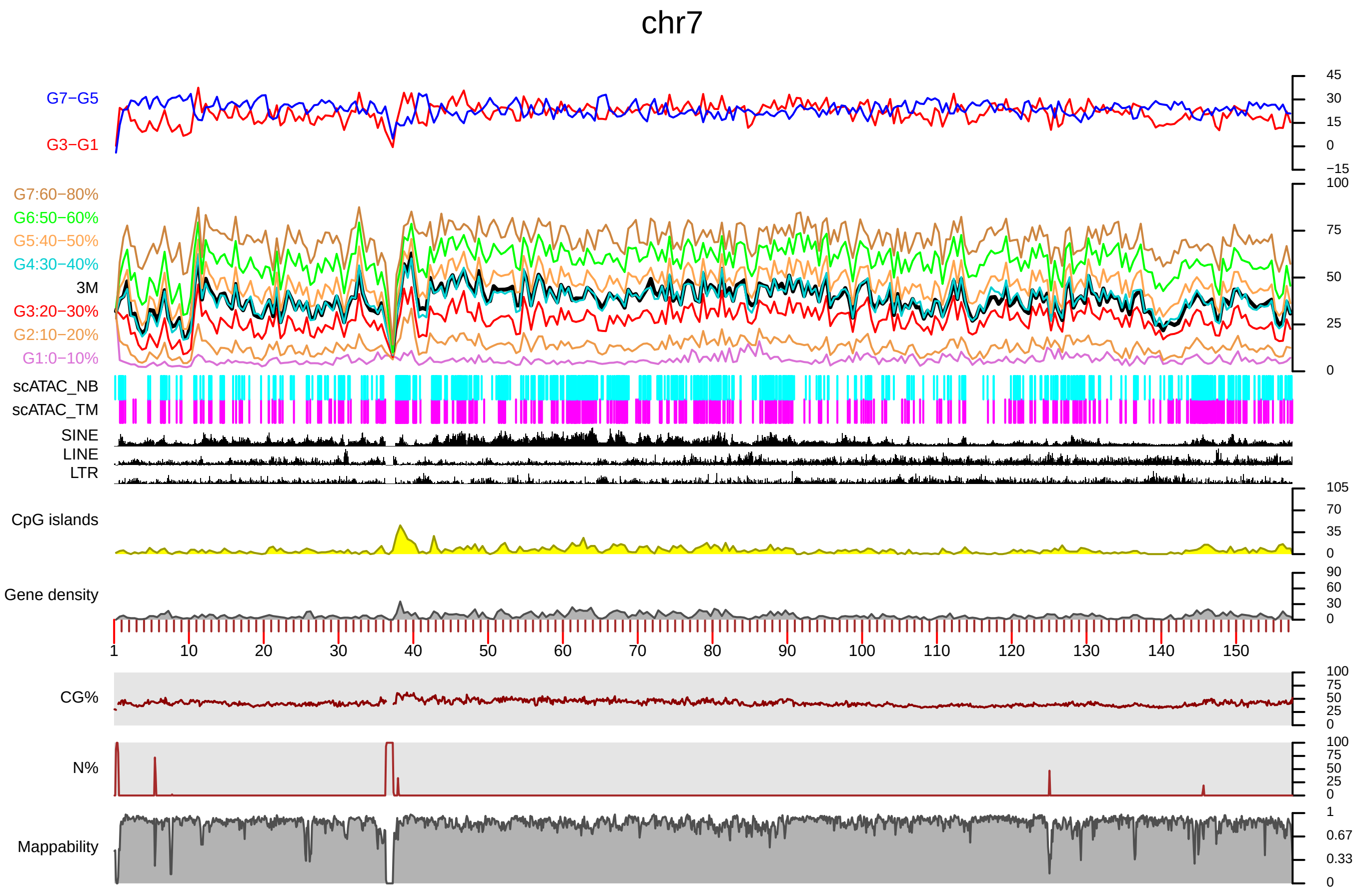
chr7
G7−G5
G3−G1
G7:60−80%
G6:50−60%
G5:40−50%
G4:30−40%
3M
G3:20−30%
G2:10−20%
G1:0−10%
scATAC_NB
scATAC_TM
SINE
LINE
LTR
CpG islands
Gene density
CG%
N%
Mappability

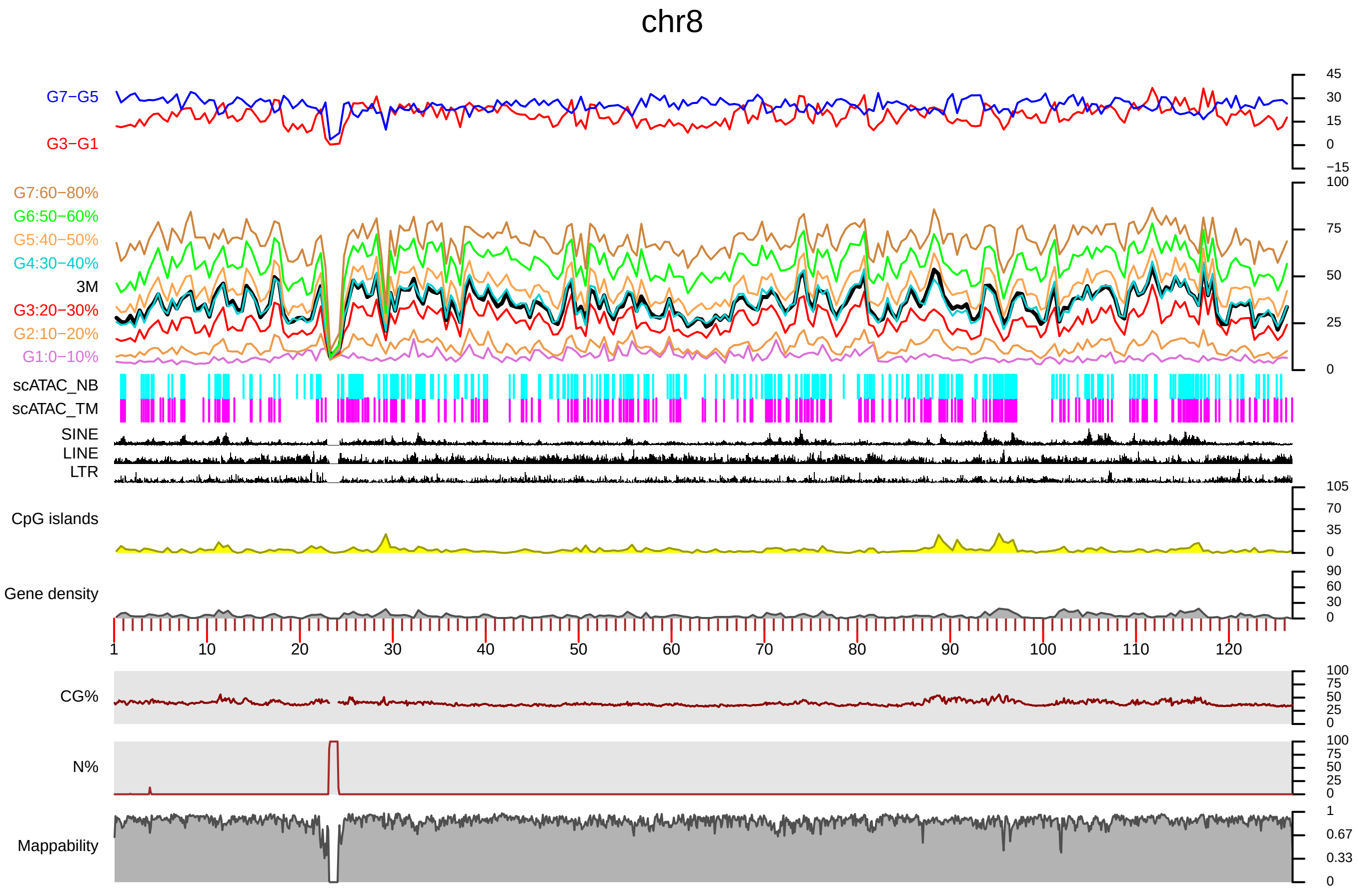
chr8
G7−G5
G3−G1
G7:60−80%
G6:50−60%
G5:40−50%
G4:30−40%
3M
G3:20−30%
G2:10−20%
G1:0−10%
scATAC_NB
scATAC_TM
SINE
LINE
LTR
CpG islands
Gene density
CG%
N%
Mappability

chr9

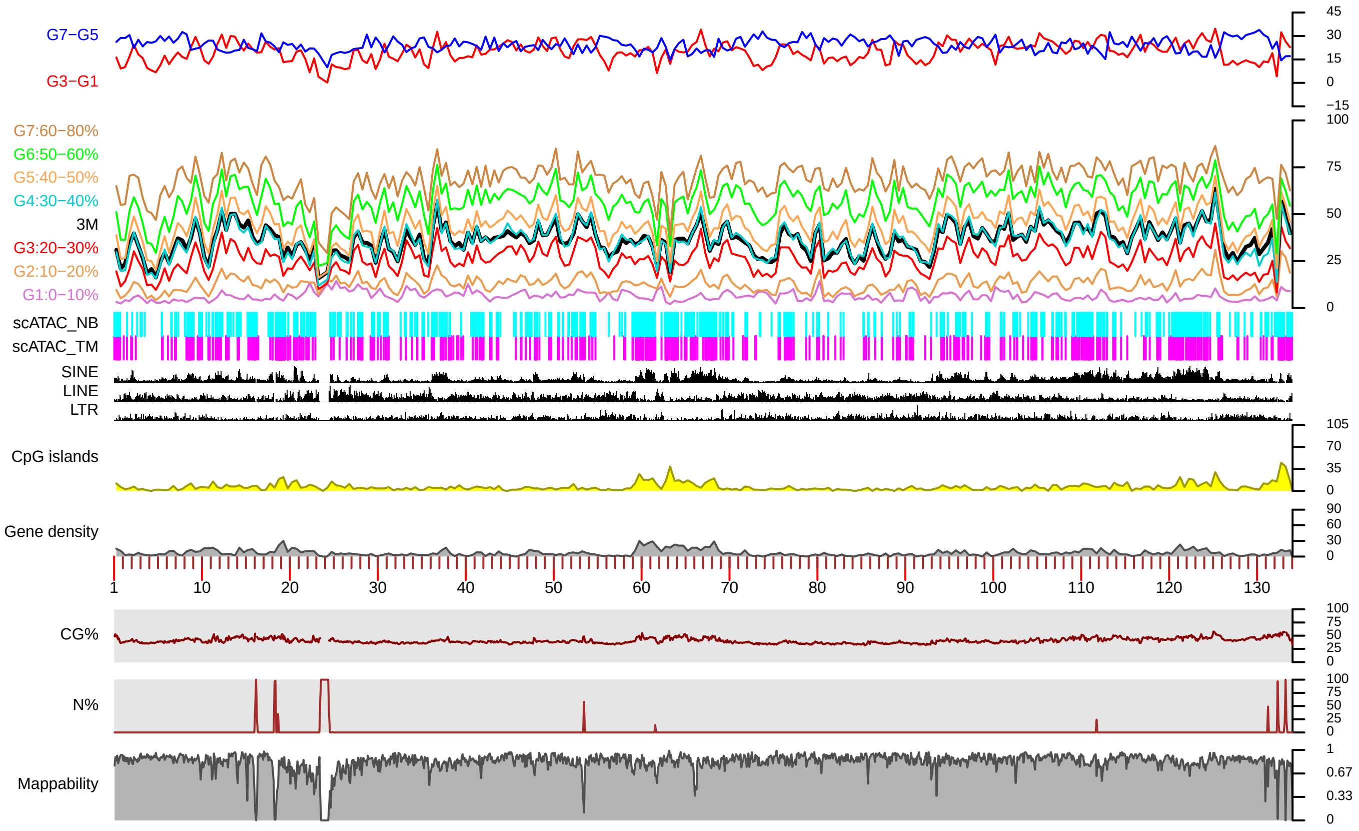
G7−G5
G3−G1
G7:60−80%
G6:50−60%
G5:40−50%
G4:30−40%
3M
G3:20−30%
G2:10−20%
G1:0−10%
scATAC_NB
scATAC_TM
SINE
LINE
LTR
CpG islands
Gene density
CG%
N%
Mappability

chr10

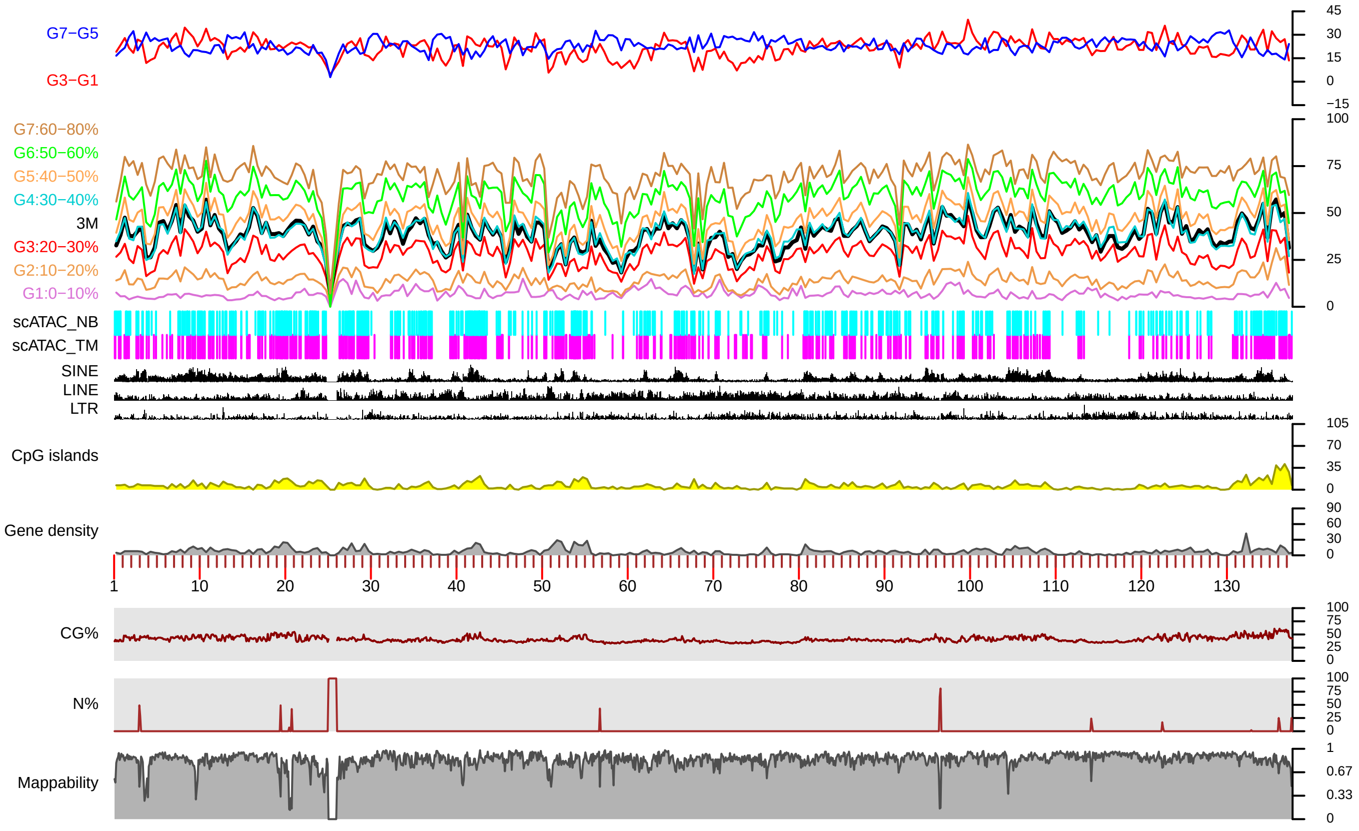
G7−G5
G3−G1
G7:60−80%
G6:50−60%
G5:40−50%
G4:30−40%
3M
G3:20−30%
G2:10−20%
G1:0−10%
scATAC_NB
scATAC_TM
SINE
LINE
LTR
CpG islands
Gene density
CG%
N%
Mappability

chr11

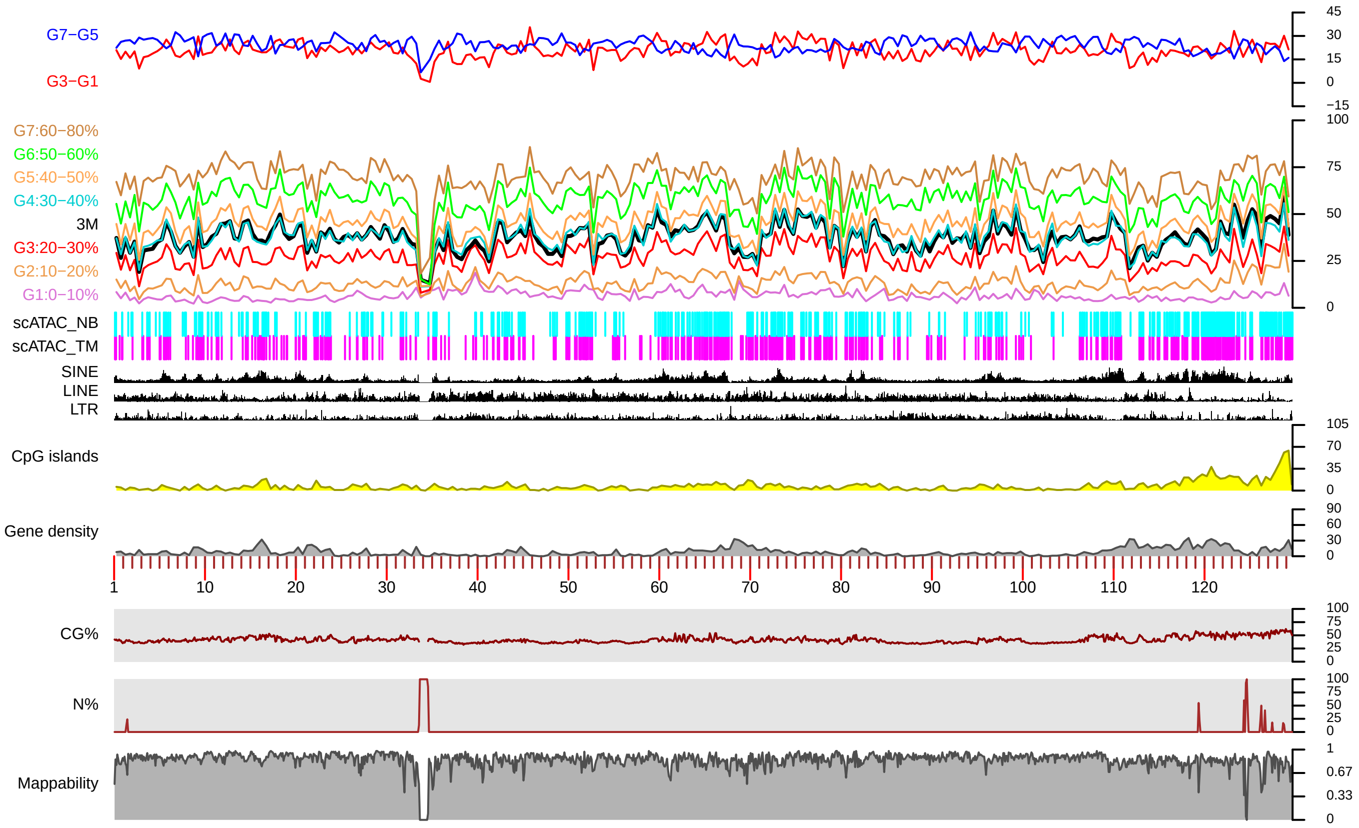
G7−G5
G3−G1
G7:60−80%
G6:50−60%
G5:40−50%
G4:30−40%
3M
G3:20−30%
G2:10−20%
G1:0−10%
scATAC_NB
scATAC_TM
SINE
LINE
LTR
CpG islands
Gene density
CG%
N%
Mappability
45
30
15
0
−15
100
75
50
25
0
105
70
35
0
90
60
30
0
1
10
20
30
40
50
60
70
80
90
100
110
120
0.67
0.33

chr12

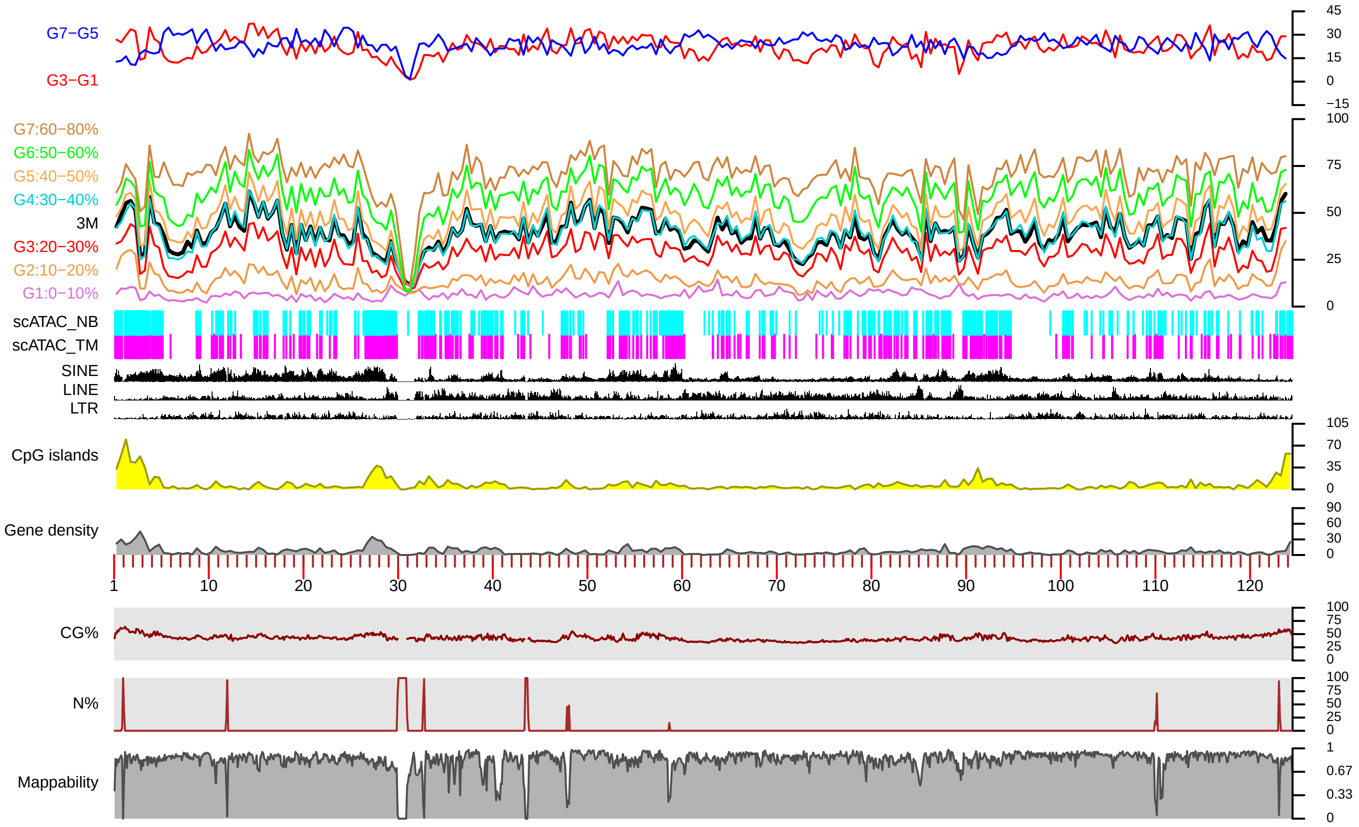
G7−G5
G3−G1
G7:60−80%
G6:50−60%
G5:40−50%
G4:30−40%
3M
G3:20−30%
G2:10−20%
G1:0−10%
scATAC_NB
scATAC_TM
SINE
LINE
LTR
CpG islands
Gene density
CG%
N%
Mappability
45
30
15
0
−15
100
75
50
25
0
105
70
35
0
90
60
30
0
1
10
20
30
40
50
60
70
80
90
100
110
120
0.67
0.33

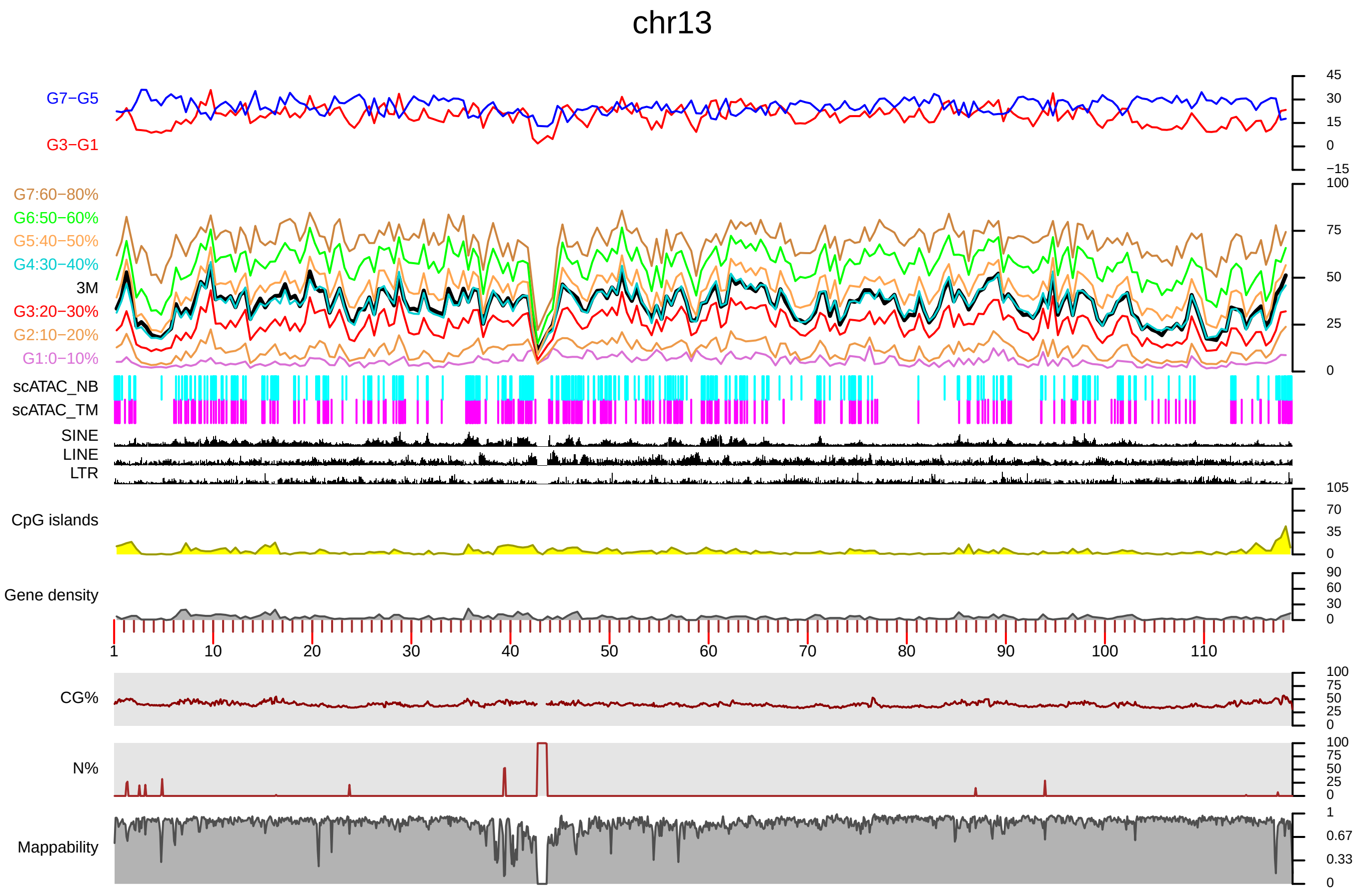
chr13
G7−G5
G3−G1
G7:60−80%
G6:50−60%
G5:40−50%
G4:30−40%
3M
G3:20−30%
G2:10−20%
G1:0−10%
scATAC_NB
scATAC_TM
SINE
LINE
LTR
CpG islands
Gene density
CG%
N%
Mappability

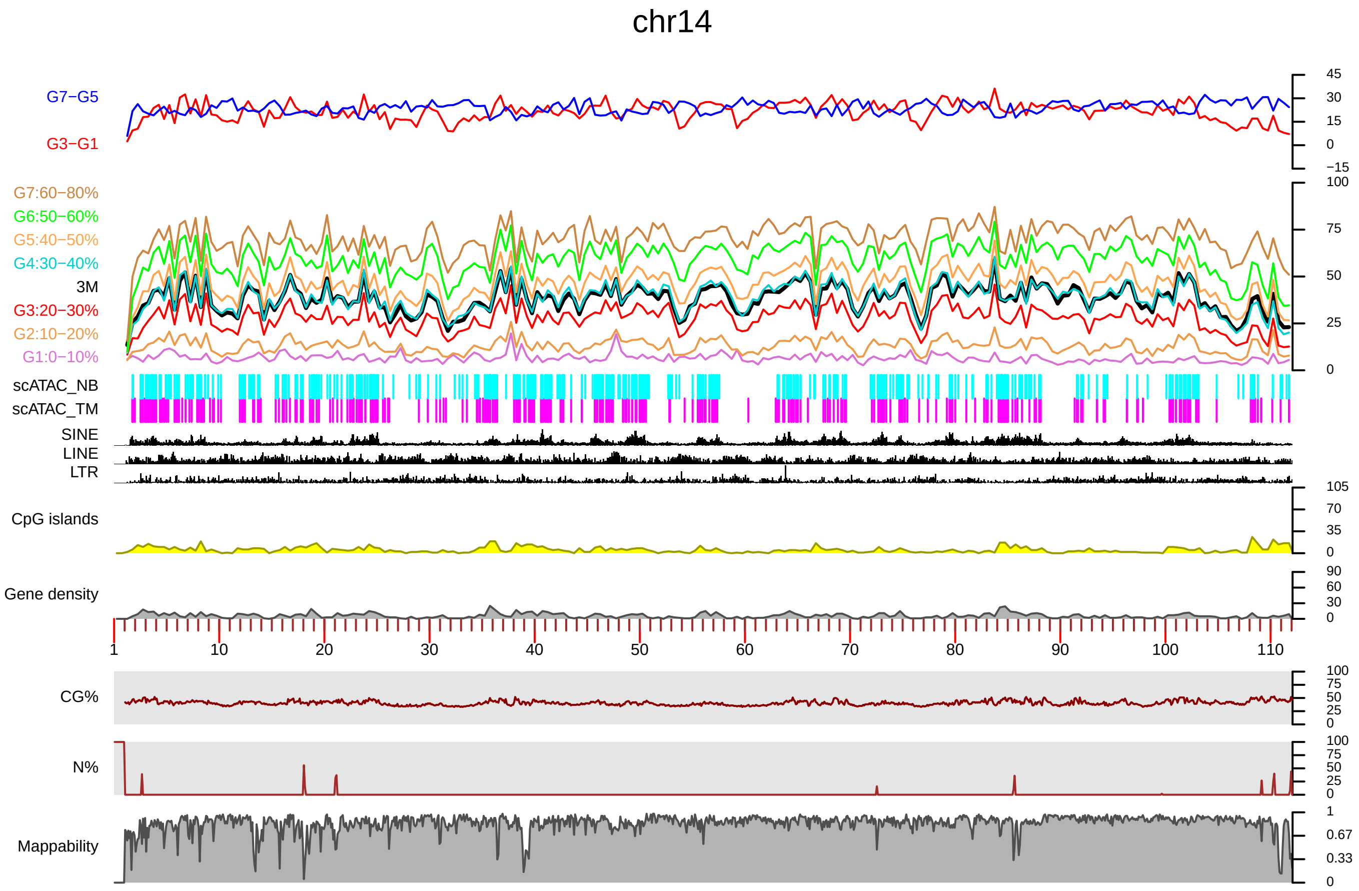
chr14
G7−G5
G3−G1
G7:60−80%
G6:50−60%
G5:40−50%
G4:30−40%
3M
G3:20−30%
G2:10−20%
G1:0−10%
scATAC_NB
scATAC_TM
SINE
LINE
LTR
CpG islands
Gene density
CG%
N%
Mappability

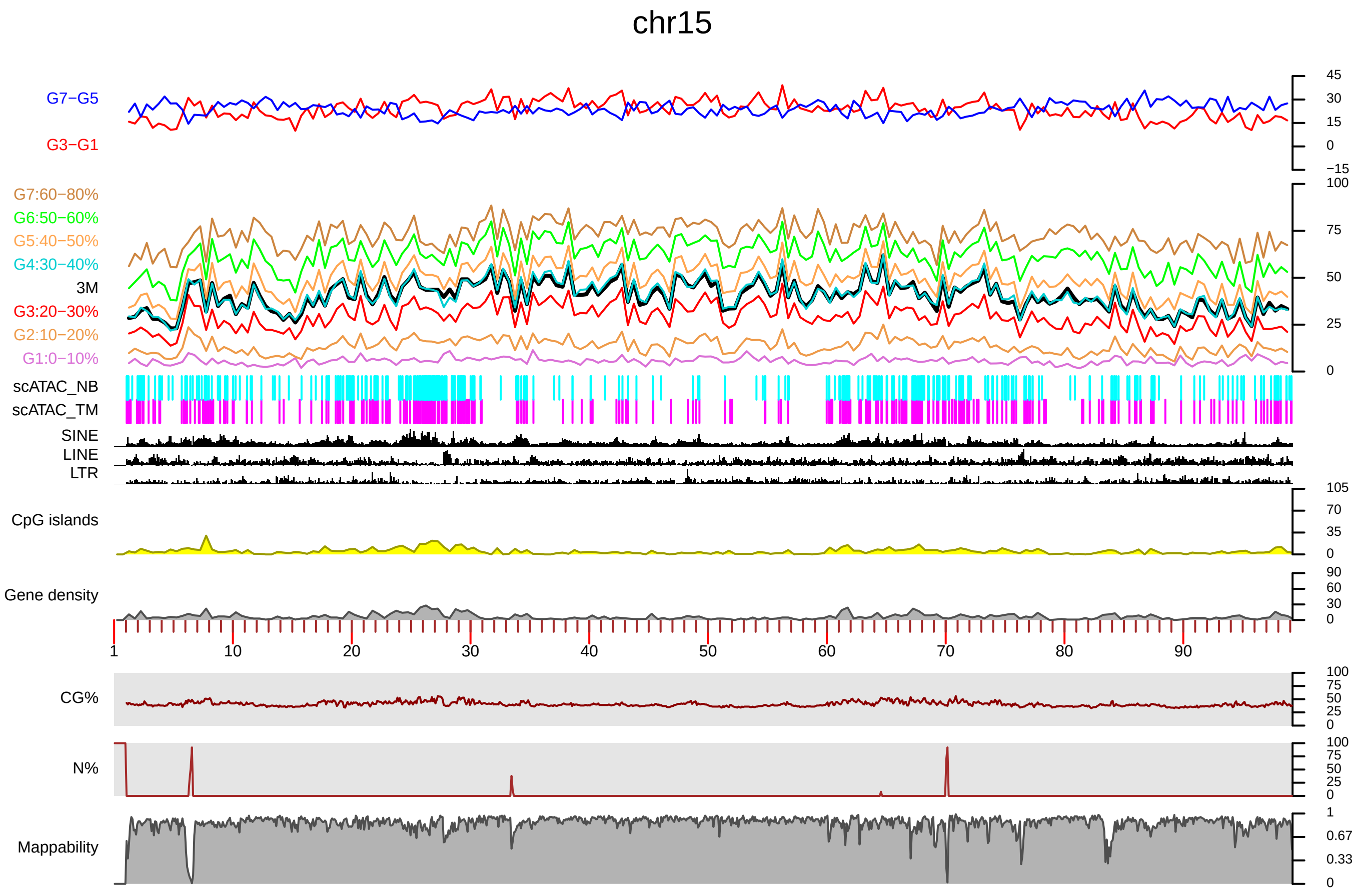
chr15
G7−G5
G3−G1
45
30
15
0
−15
G7:60−80%
G6:50−60%
G5:40−50%
G4:30−40%
3M
G3:20−30%
G2:10−20%
G1:0−10%
100
75
50
25
0
scATAC_NB
scATAC_TM
SINE
LINE
LTR
CpG islands
105
70
35
0
Gene density
90
60
30
0
1
10
20
30
40
50
60
70
80
90
CG%
100
75
50
25
0
N%
100
75
50
25
0
Mappability
1
0.67
0.33
0

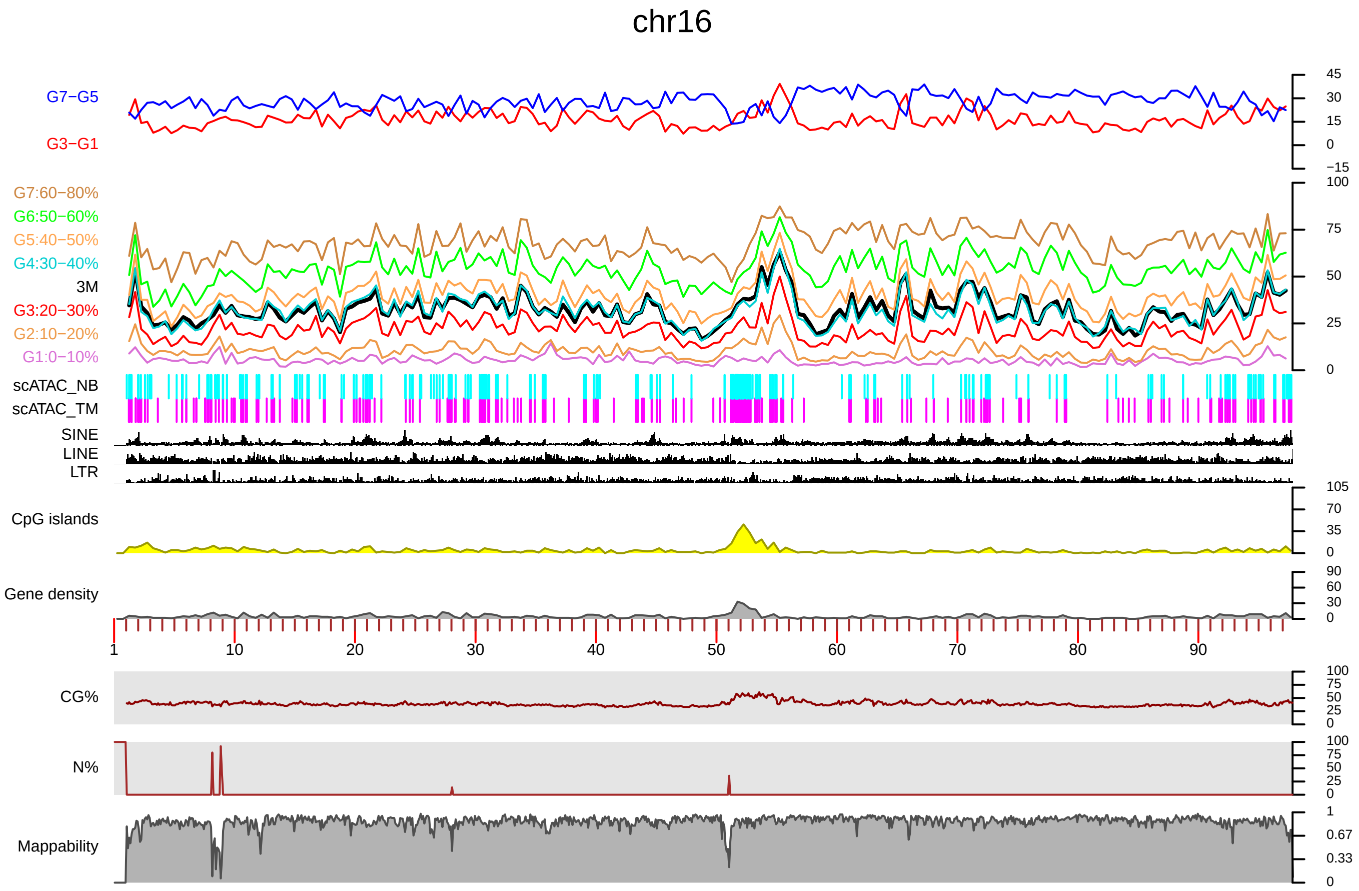
chr16
G7−G5
G3−G1
45
30
15
0
−15
G7:60−80%
G6:50−60%
G5:40−50%
G4:30−40%
3M
G3:20−30%
G2:10−20%
G1:0−10%
100
75
50
25
0
scATAC_NB
scATAC_TM
SINE
LINE
LTR
CpG islands
105
70
35
0
Gene density
90
60
30
0
1
10
20
30
40
50
60
70
80
90
CG%
100
75
50
25
0
N%
100
75
50
25
0
Mappability
1
0.67
0.33
0

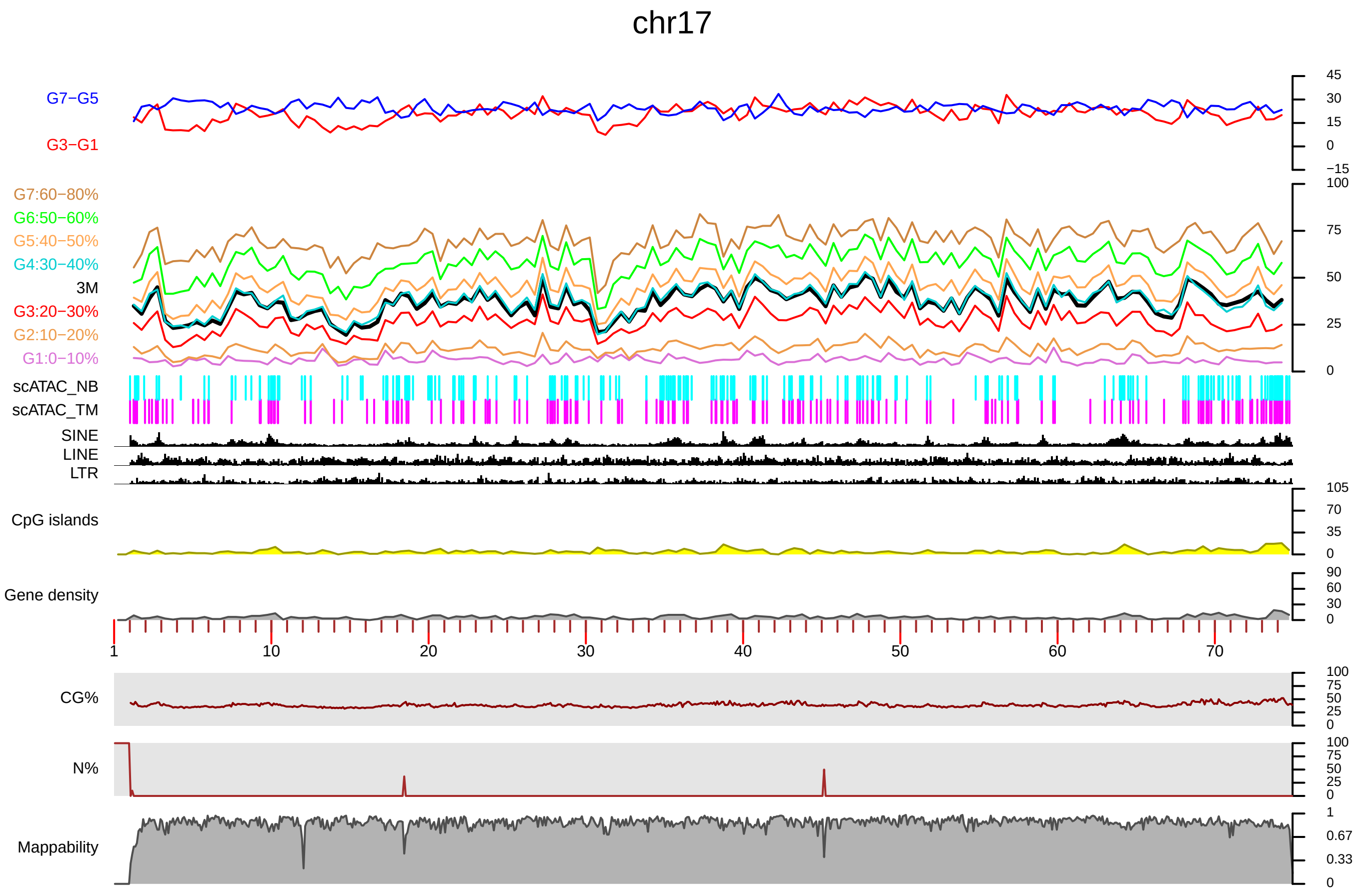
chr17
G7−G5
G3−G1
G7:60−80%
G6:50−60%
G5:40−50%
G4:30−40%
3M
G3:20−30%
G2:10−20%
G1:0−10%
scATAC_NB
scATAC_TM
SINE
LINE
LTR
CpG islands
Gene density
CG%
N%
Mappability

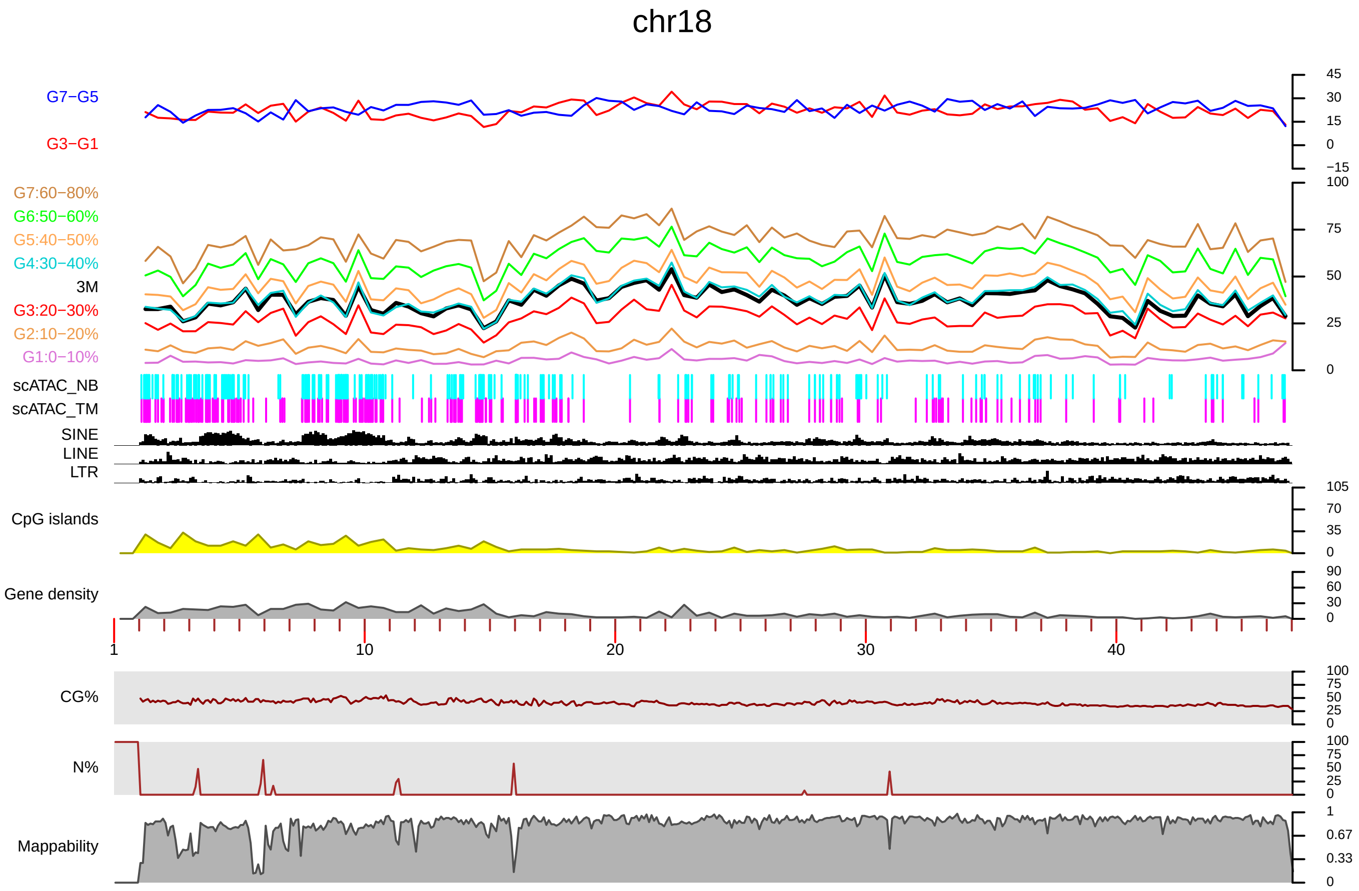
chr18
G7−G5
G3−G1
G7:60−80%
G6:50−60%
G5:40−50%
G4:30−40%
3M
G3:20−30%
G2:10−20%
G1:0−10%
scATAC_NB
scATAC_TM
SINE
LINE
LTR
CpG islands
Gene density
CG%
N%
Mappability

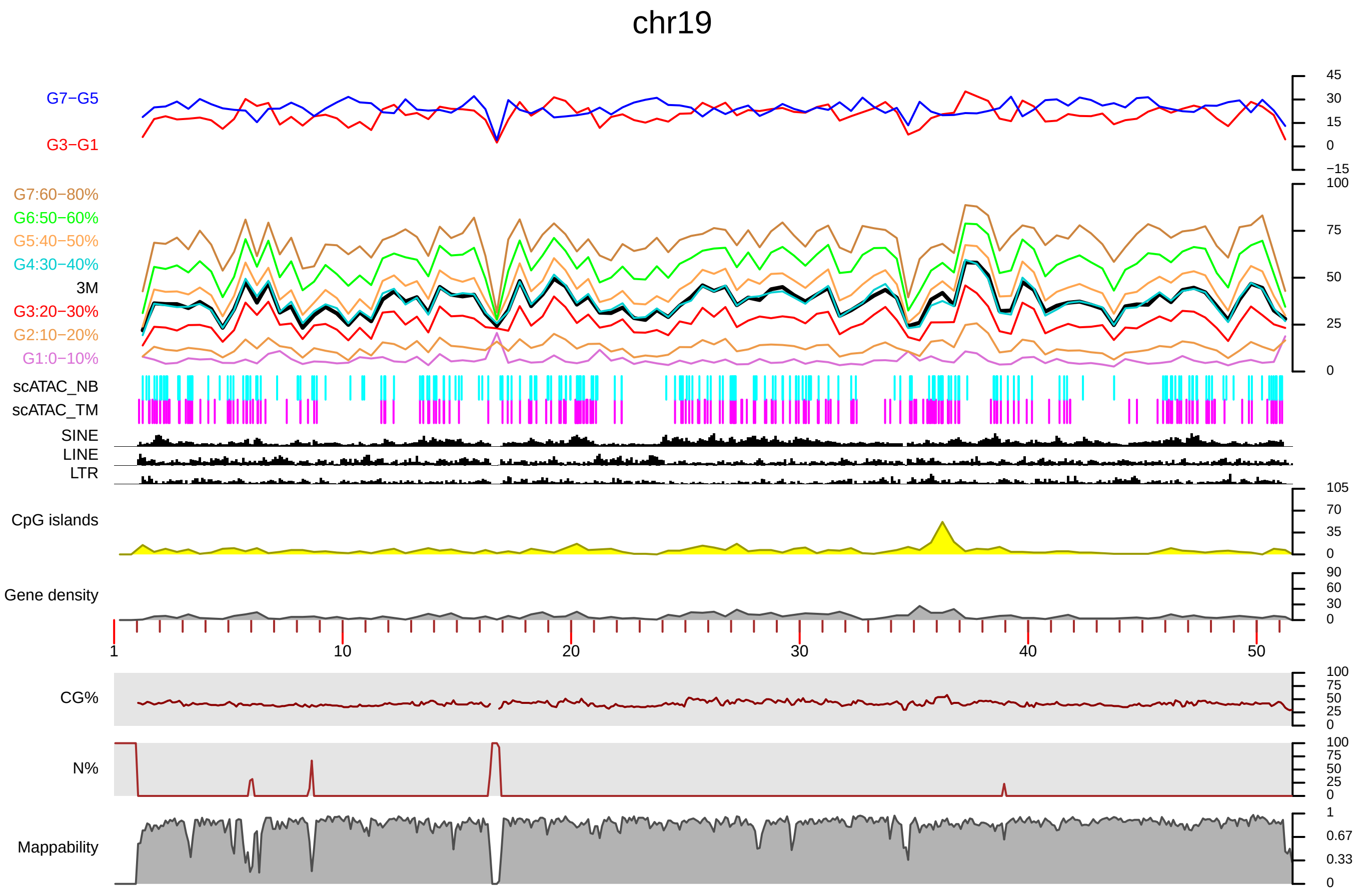
chr19
G7−G5
G3−G1
G7:60−80%
G6:50−60%
G5:40−50%
G4:30−40%
3M
G3:20−30%
G2:10−20%
G1:0−10%
scATAC_NB
scATAC_TM
SINE
LINE
LTR
CpG islands
Gene density
CG%
N%
Mappability
45
30
15
0
−15
100
75
50
25
0
105
70
35
0
90
60
30
0
1
10
20
30
40
50
100
75
50
25
0
100
75
50
25
0
1
0.67
0.33
0

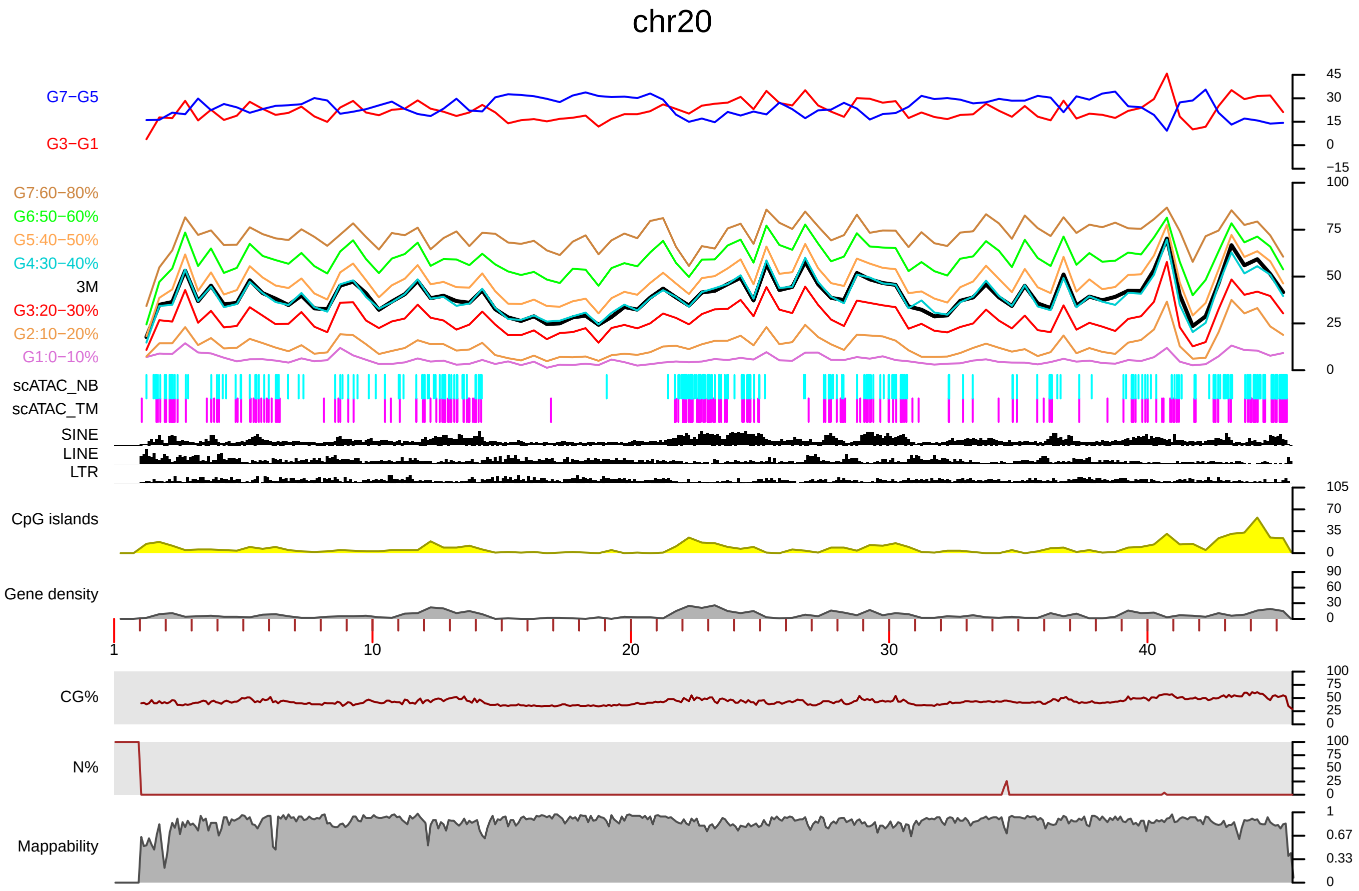
chr20
G7−G5
G3−G1
G7:60−80%
G6:50−60%
G5:40−50%
G4:30−40%
3M
G3:20−30%
G2:10−20%
G1:0−10%
scATAC_NB
scATAC_TM
SINE
LINE
LTR
CpG islands
Gene density
CG%
N%
Mappability
45
30
15
0
−15
100
75
50
25
0
105
70
35
0
90
60
30
0
1
10
20
30
40
100
75
50
25
0
100
75
50
25
0
1
0.67
0.33
0

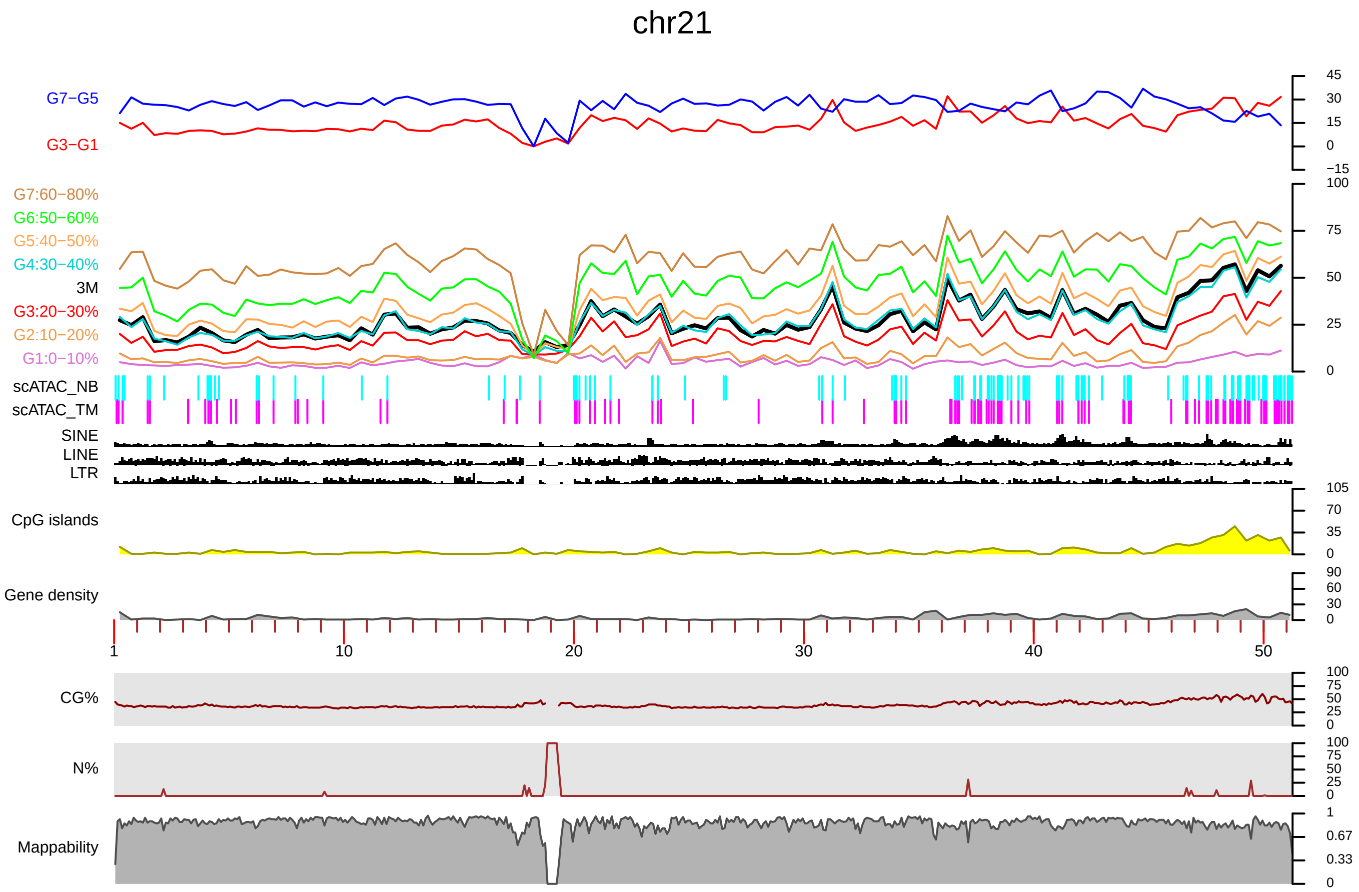
chr21
G7−G5
G3−G1
G7:60−80%
G6:50−60%
G5:40−50%
G4:30−40%
3M
G3:20−30%
G2:10−20%
G1:0−10%
scATAC_NB
scATAC_TM
SINE
LINE
LTR
CpG islands
Gene density
CG%
N%
Mappability
45
30
15
0
−15
100
75
50
25
0
105
70
35
0
90
60
30
0
1
10
20
30
40
50
100
75
50
25
0
1
0.67
0.33
0

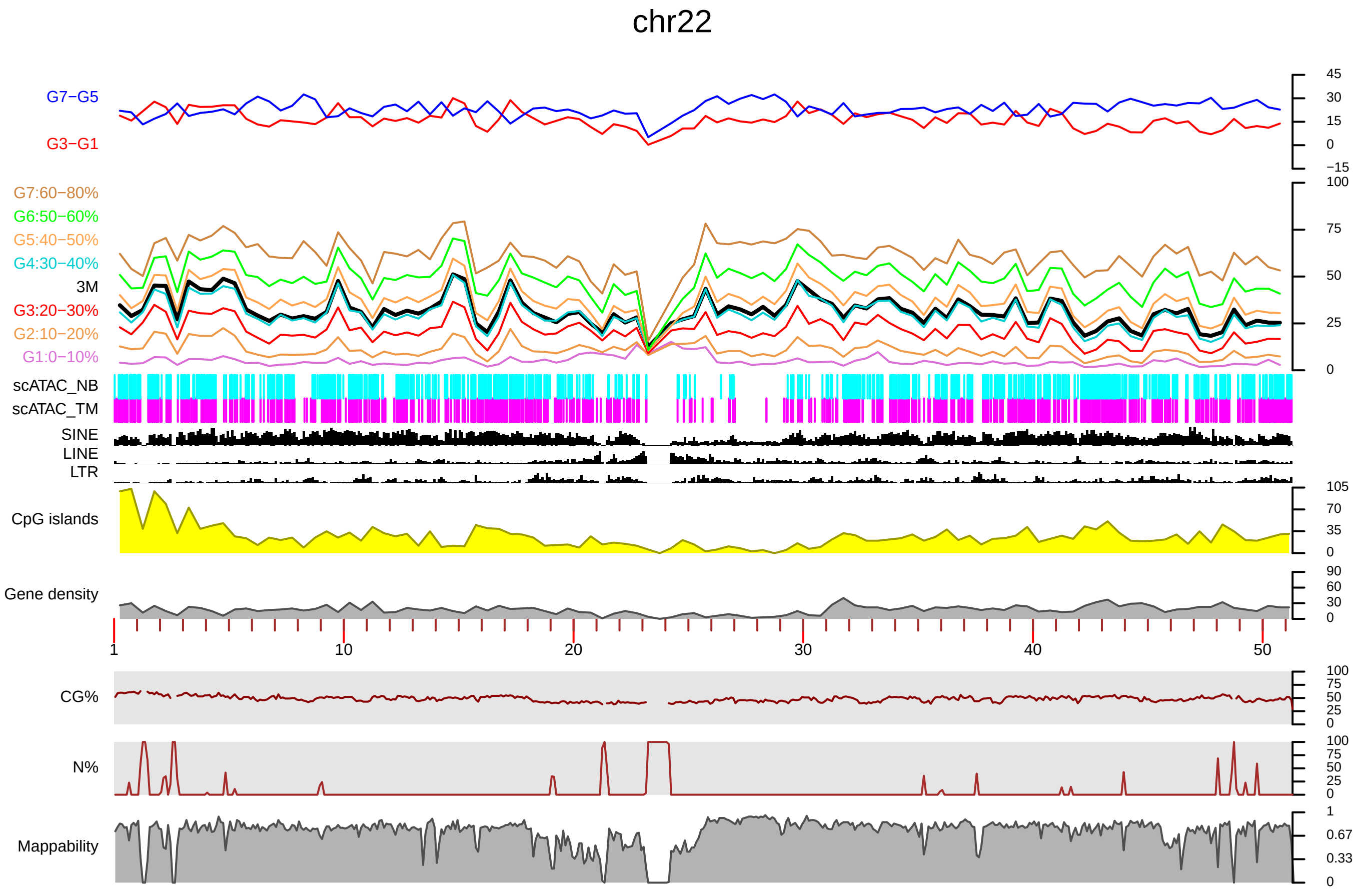
chr22
G7−G5
G3−G1
G7:60−80%
G6:50−60%
G5:40−50%
G4:30−40%
3M
G3:20−30%
G2:10−20%
G1:0−10%
scATAC_NB
scATAC_TM
SINE
LINE
LTR
CpG islands
Gene density
CG%
N%
Mappability
45
30
15
0
−15
100
75
50
25
0
105
70
35
0
90
60
30
0
1
10
20
30
40
50
100
75
50
25
0
1
0.67
0.33
0

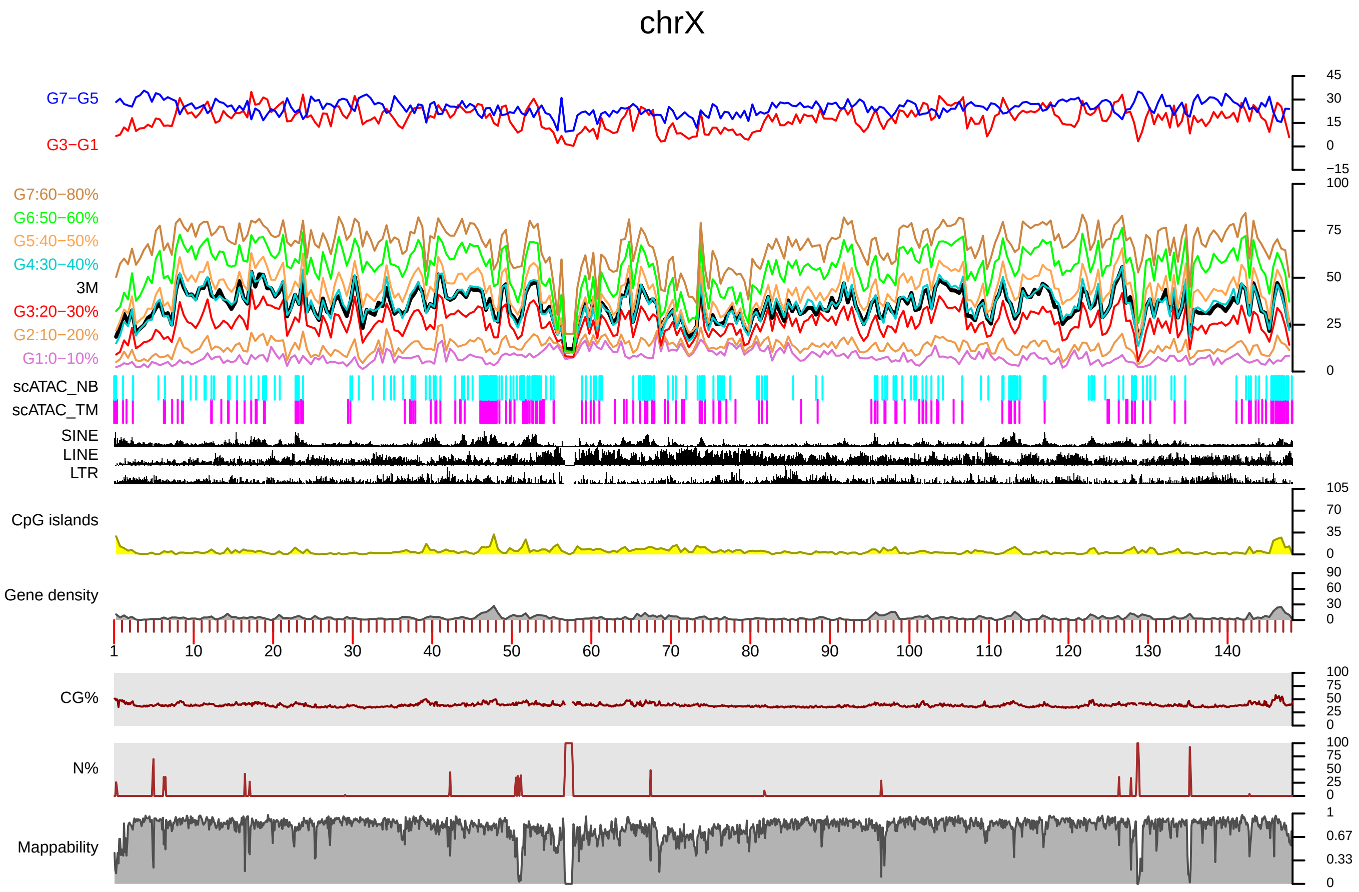
chrX
G7−G5
G3−G1
G7:60−80%
G6:50−60%
G5:40−50%
G4:30−40%
3M
G3:20−30%
G2:10−20%
G1:0−10%
scATAC_NB
scATAC_TM
SINE
LINE
LTR
CpG islands
Gene density
CG%
N%
Mappability
45
30
15
0
−15
100
75
50
25
0
105
70
35
0
90
60
30
0
1
10
20
30
40
50
60
70
80
90
100
110
120
130
140
1
0.67
0.33
0

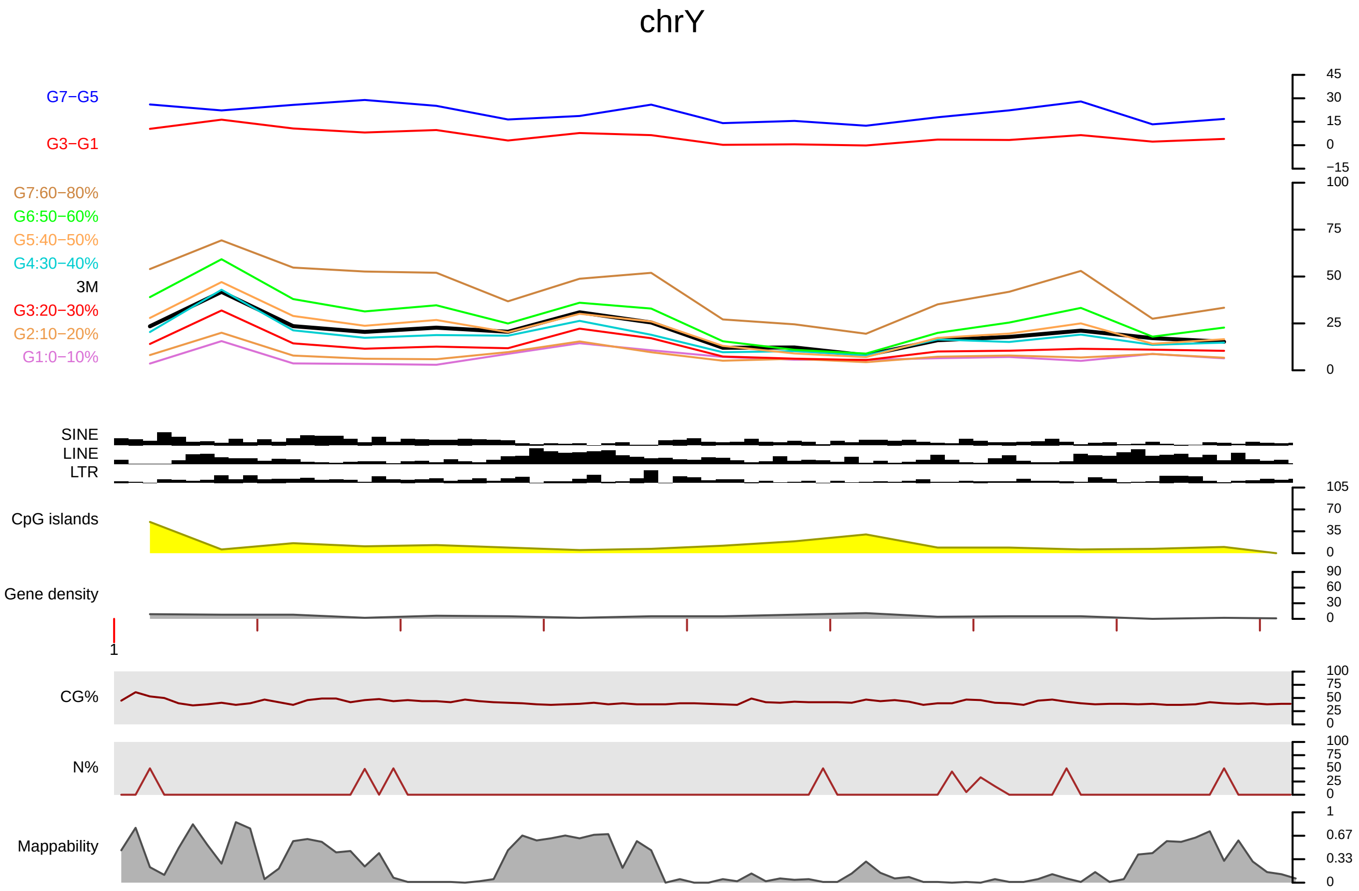
chrY
G7−G5
G3−G1
G7:60−80%
G6:50−60%
G5:40−50%
G4:30−40%
3M
G3:20−30%
G2:10−20%
G1:0−10%
SINE
LINE
LTR
CpG islands
Gene density
CG%
N%
Mappability
45
30
15
0
−15
100
75
50
25
0
105
70
35
0
90
60
30
0
1
1
0.67
0.33
0

Supplementary Figure 2

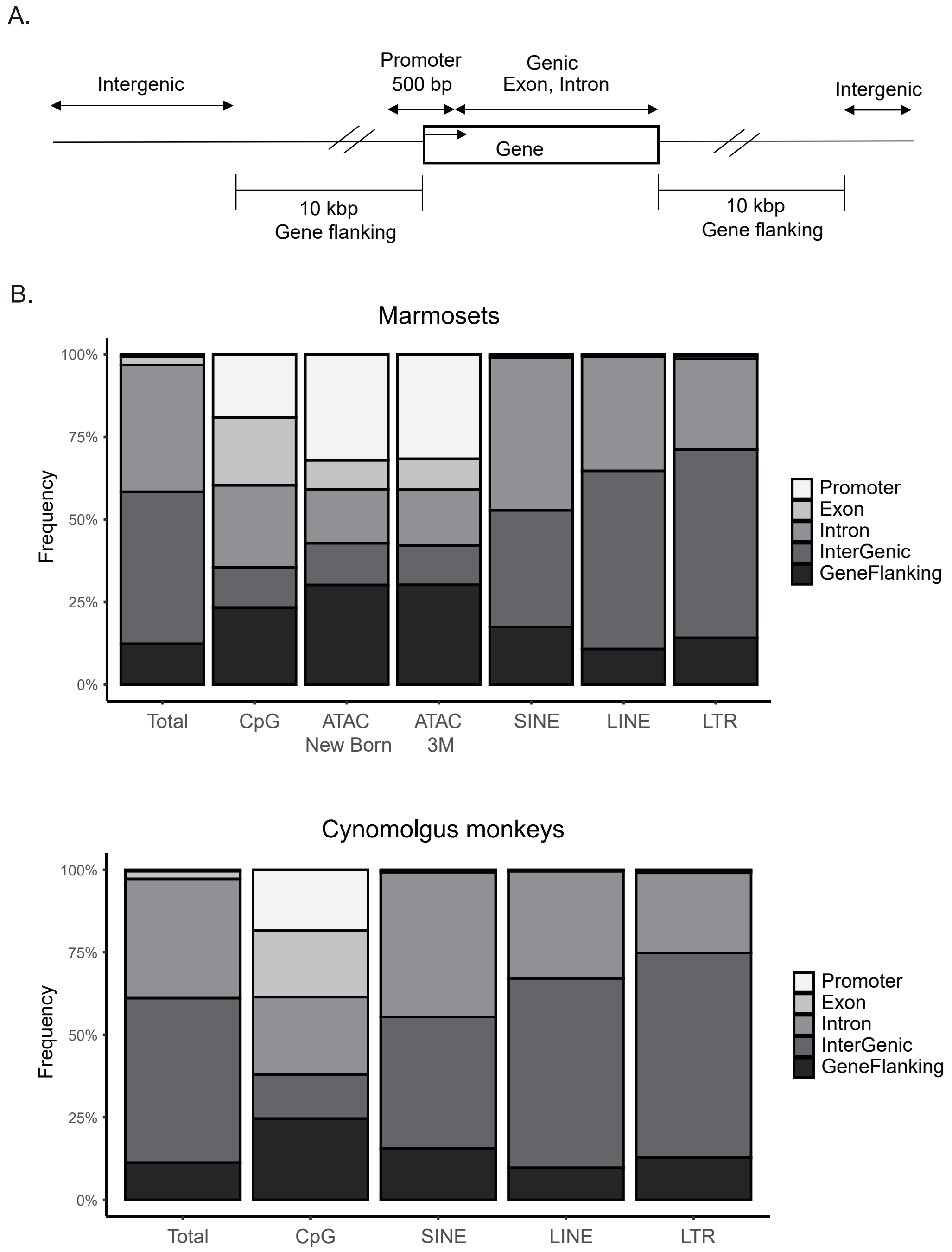

Supplementary Figure 3

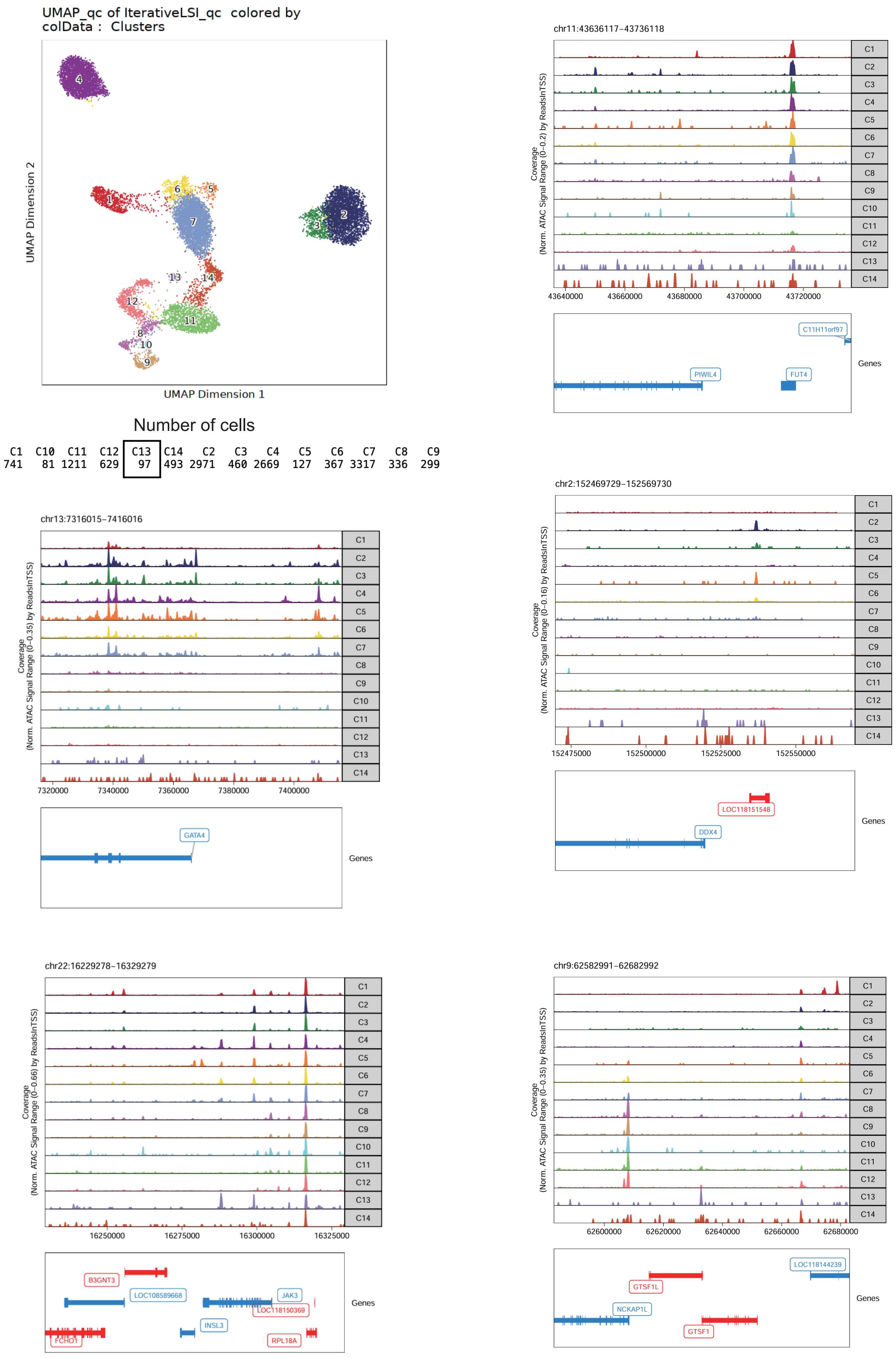

Supplementary Figure 4

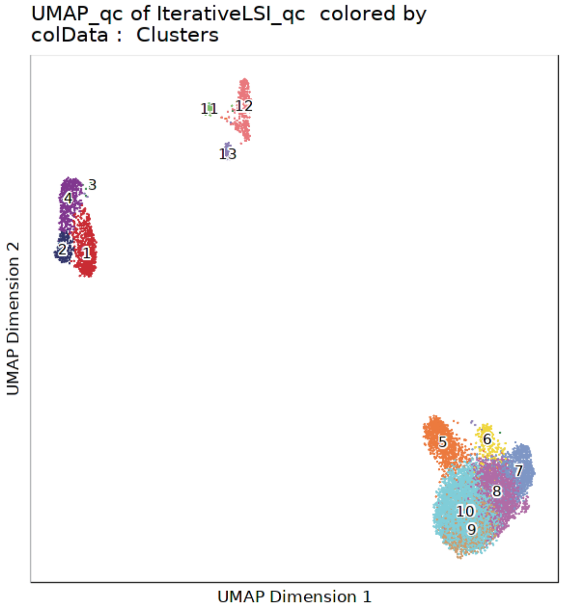

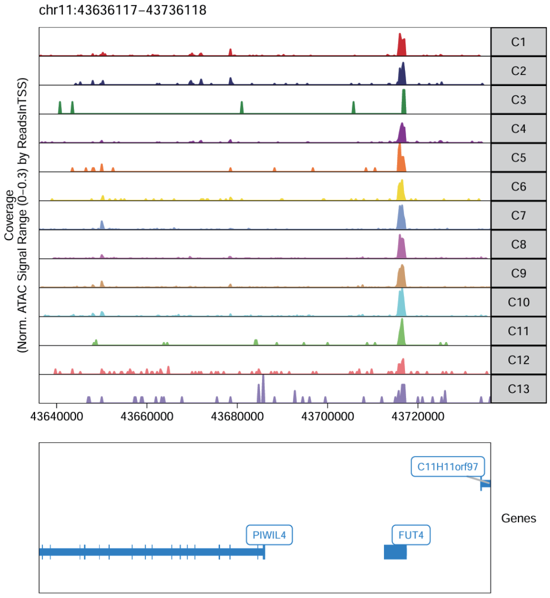

Number of cells

C1	C10	C11	C12	C13	C2	C3	C4	C5	C6	C7	C8	C9
667	3715	103	313	92	222	27	472	830	236	876	1641	703

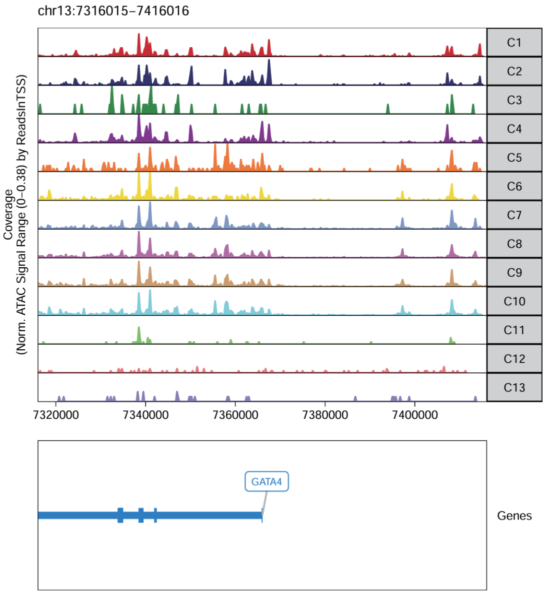

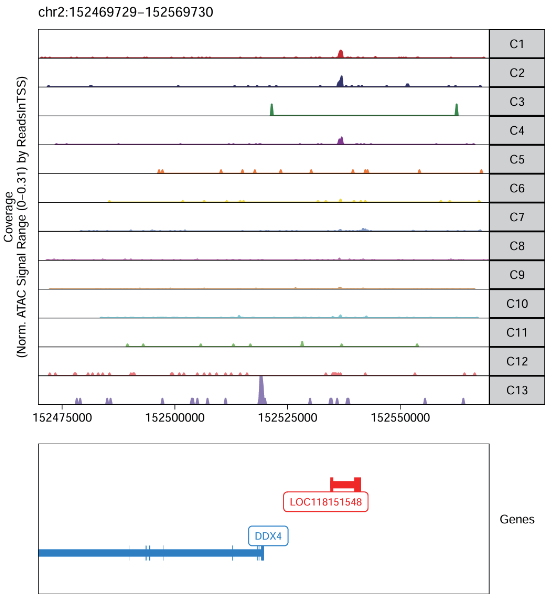

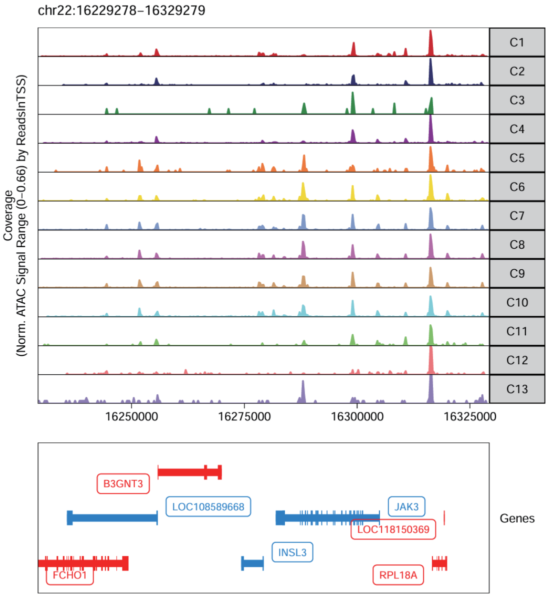

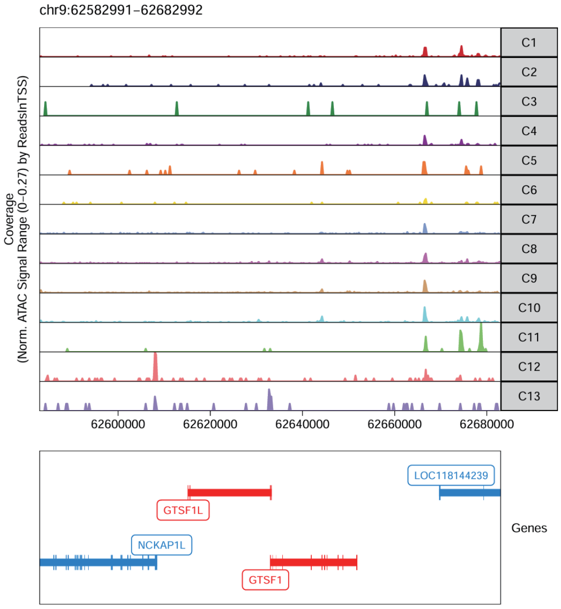

Supplementary Figure 5

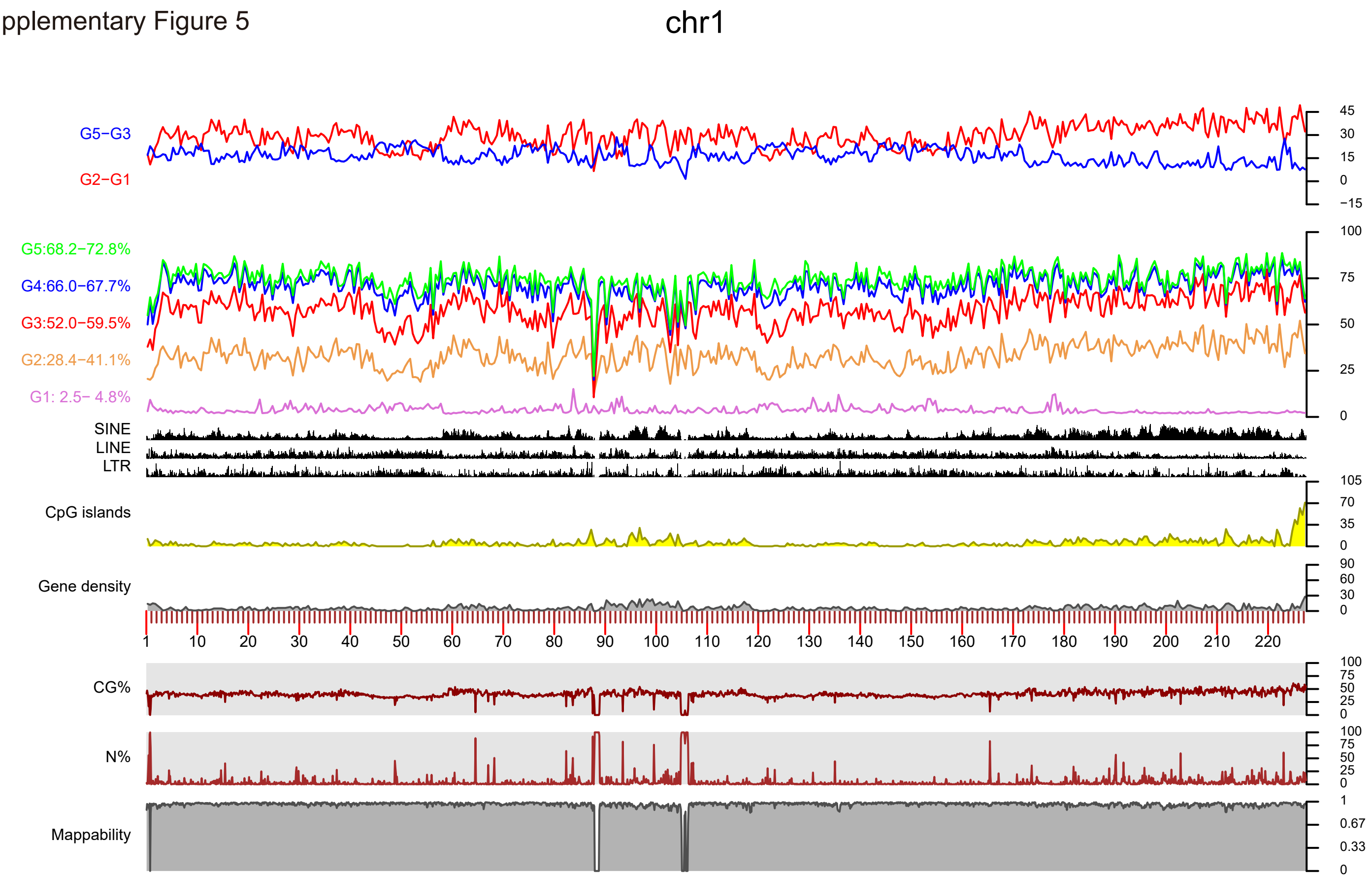

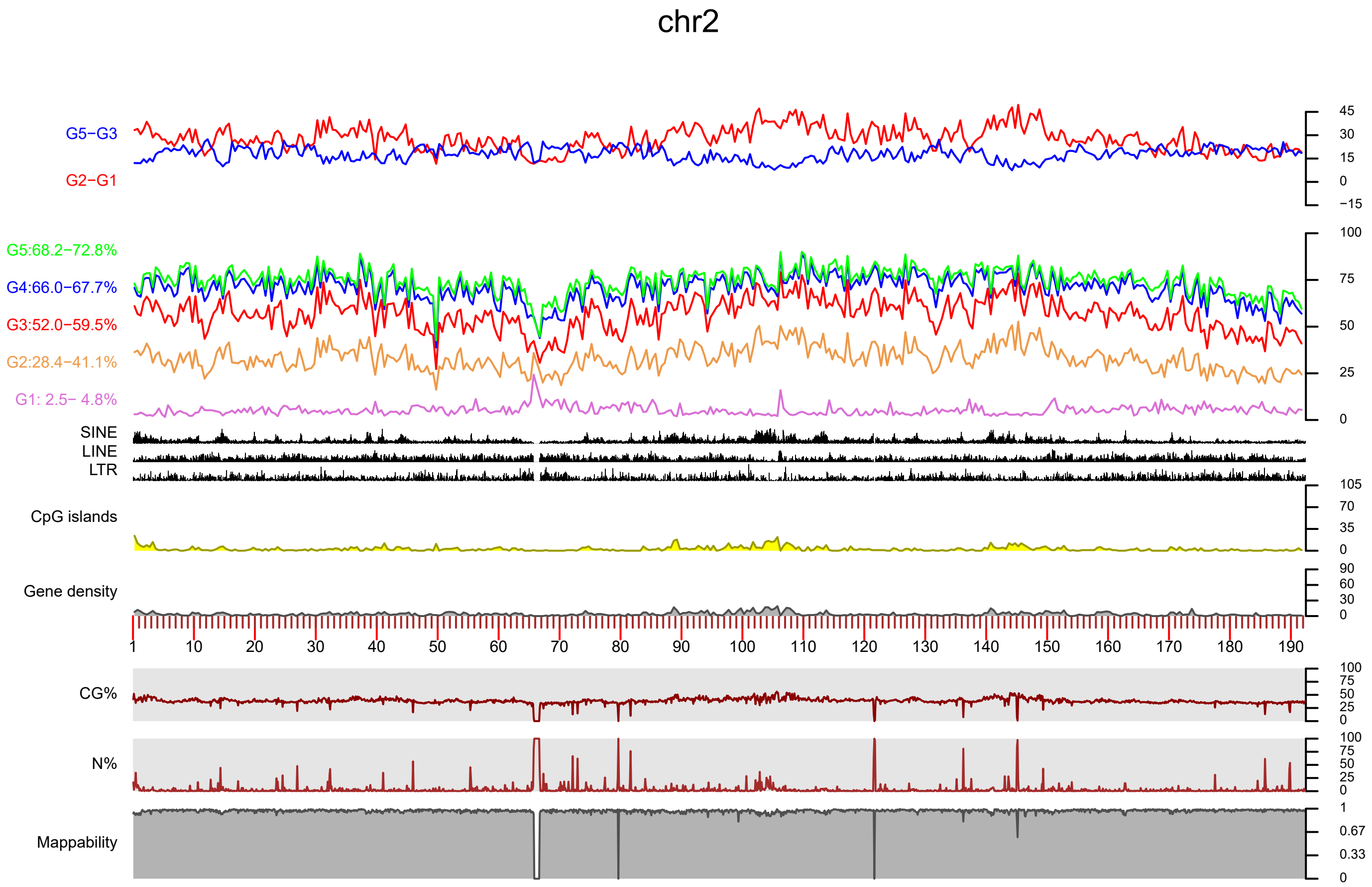

chr3

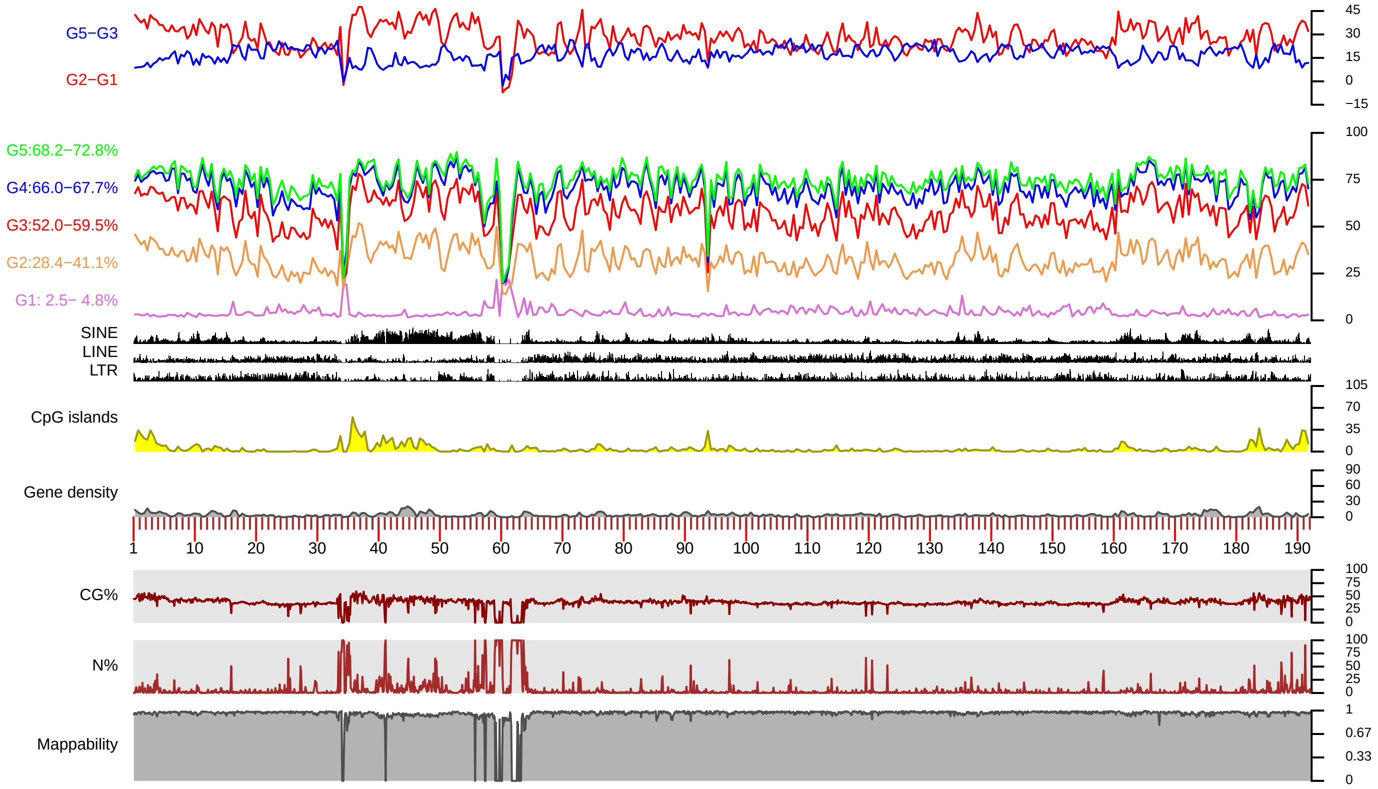
G5−G3
G2−G1
G5:68.2−72.8%
G4:66.0−67.7%
G3:52.0−59.5%
G2:28.4−41.1%
G1: 2.5− 4.8%
SINE
LINE
LTR
CpG islands
Gene density
CG%
N%
Mappability

chr4

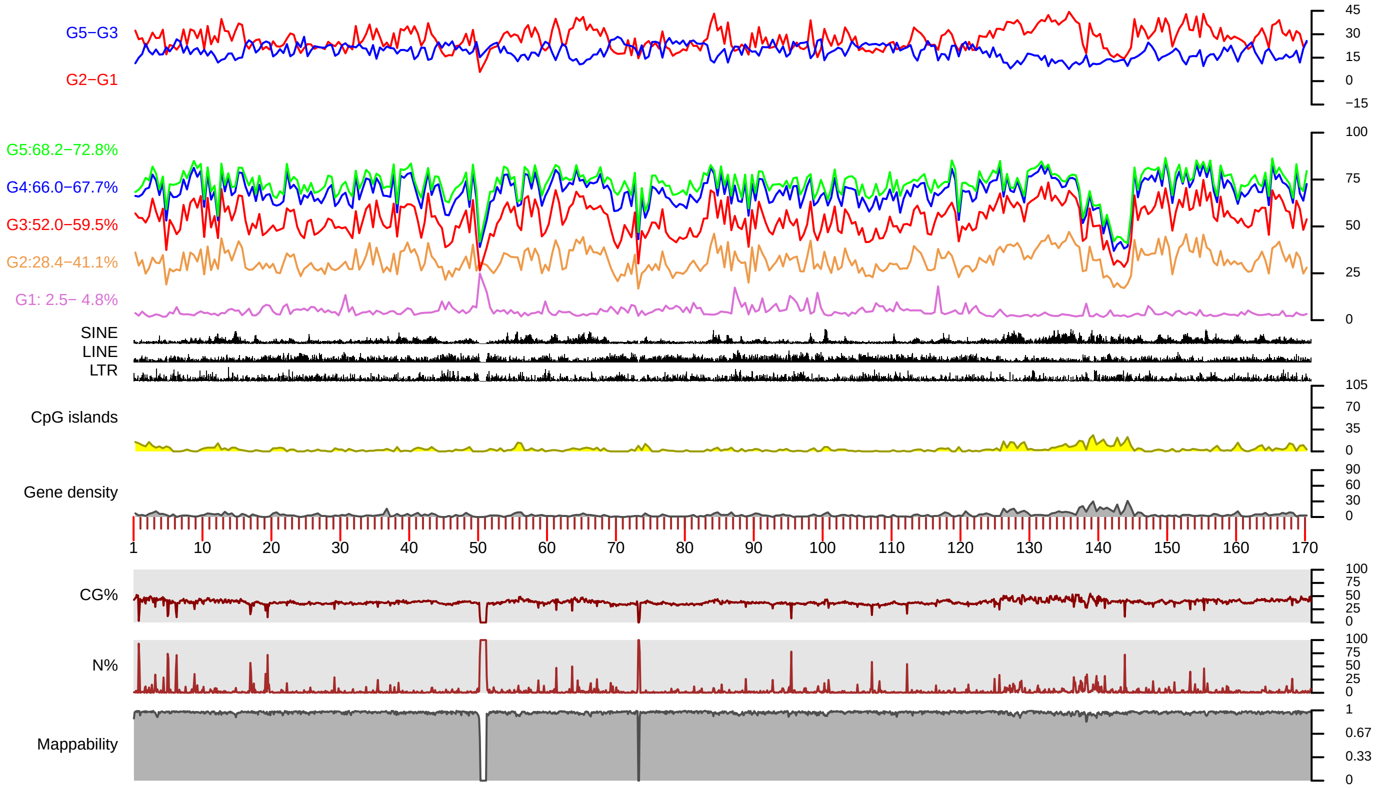
G5−G3
G2−G1
G5:68.2−72.8%
G4:66.0−67.7%
G3:52.0−59.5%
G2:28.4−41.1%
G1: 2.5− 4.8%
SINE
LINE
LTR
CpG islands
Gene density
CG%
N%
Mappability

chr5

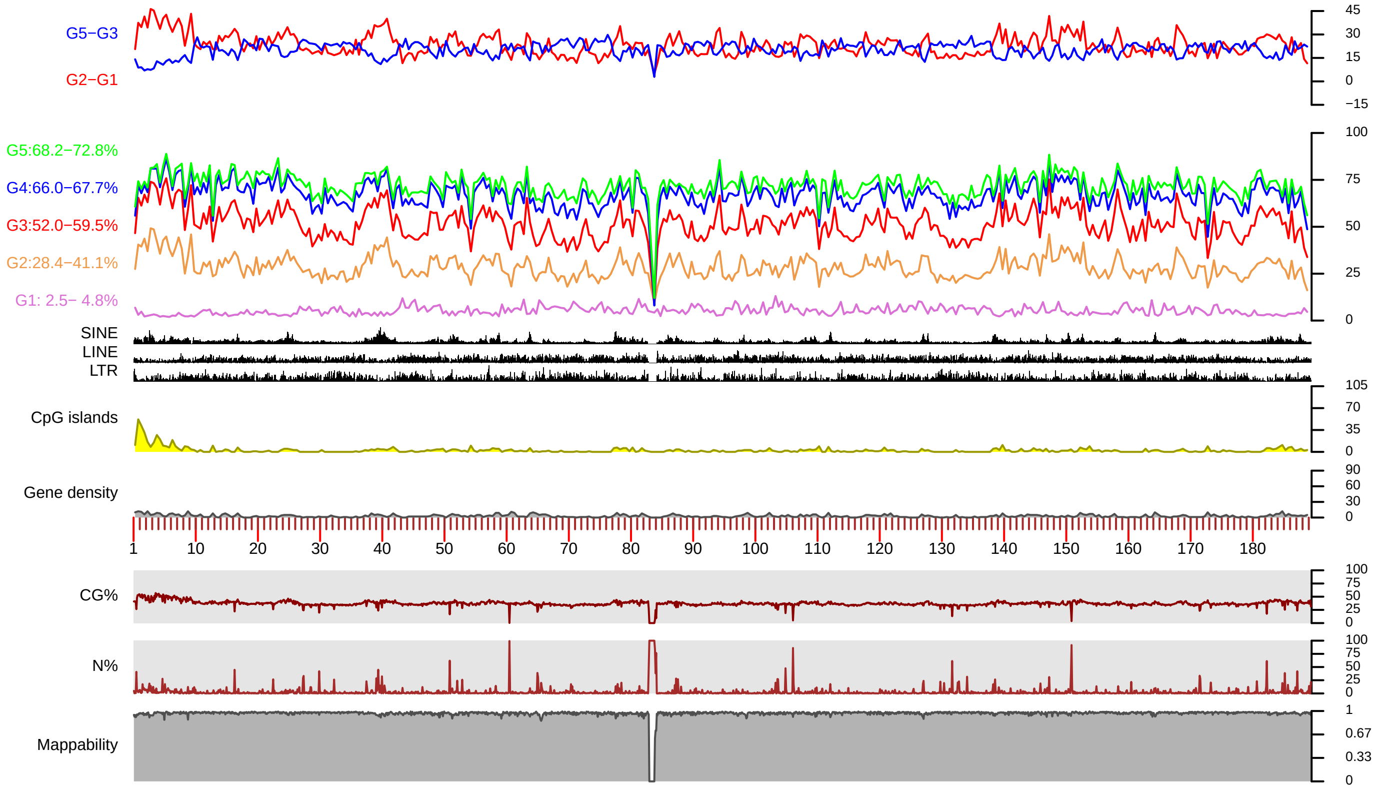

G5−G3
G2−G1
G5:68.2−72.8%
G4:66.0−67.7%
G3:52.0−59.5%
G2:28.4−41.1%
G1: 2.5− 4.8%
SINE
LINE
LTR
CpG islands
Gene density
CG%
N%
Mappability

chr6

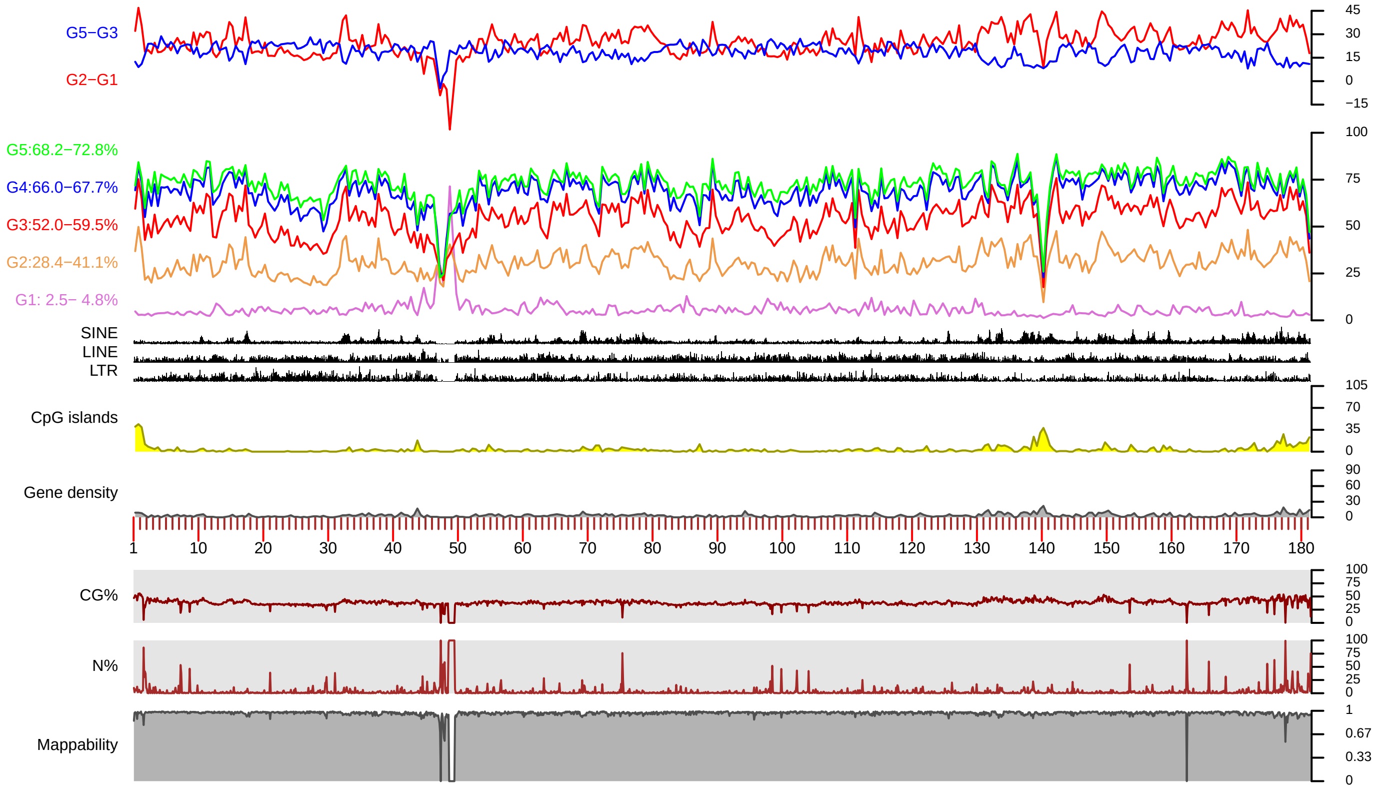

G5−G3
G2−G1
G5:68.2−72.8%
G4:66.0−67.7%
G3:52.0−59.5%
G2:28.4−41.1%
G1: 2.5− 4.8%
SINE
LINE
LTR
CpG islands
Gene density
CG%
N%
Mappability

chr7

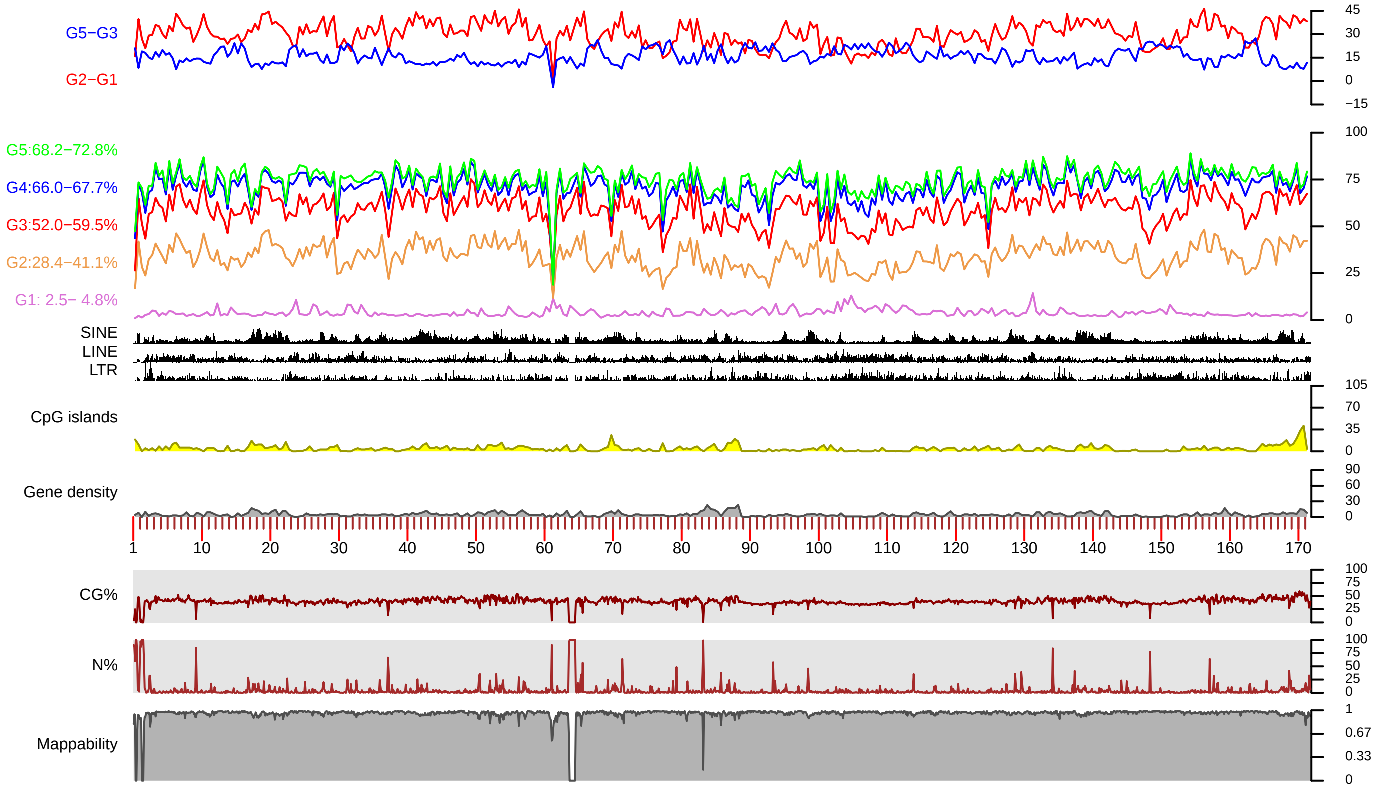
G5−G3
G2−G1
G5:68.2−72.8%
G4:66.0−67.7%
G3:52.0−59.5%
G2:28.4−41.1%
G1: 2.5− 4.8%
SINE
LINE
LTR
CpG islands
Gene density
CG%
N%
Mappability

chr8

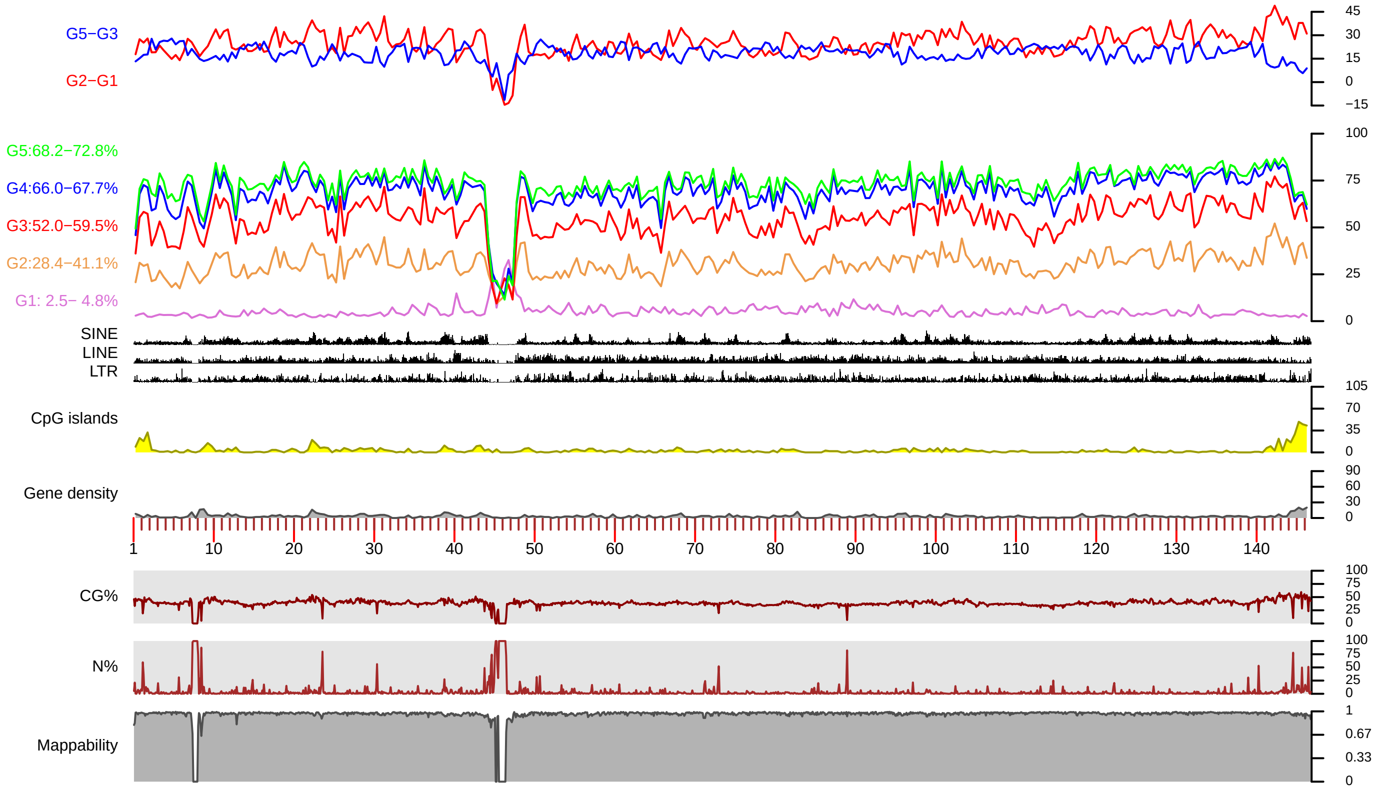
G5−G3
G2−G1
G5:68.2−72.8%
G4:66.0−67.7%
G3:52.0−59.5%
G2:28.4−41.1%
G1: 2.5− 4.8%
SINE
LINE
LTR
CpG islands
Gene density
CG%
N%
Mappability

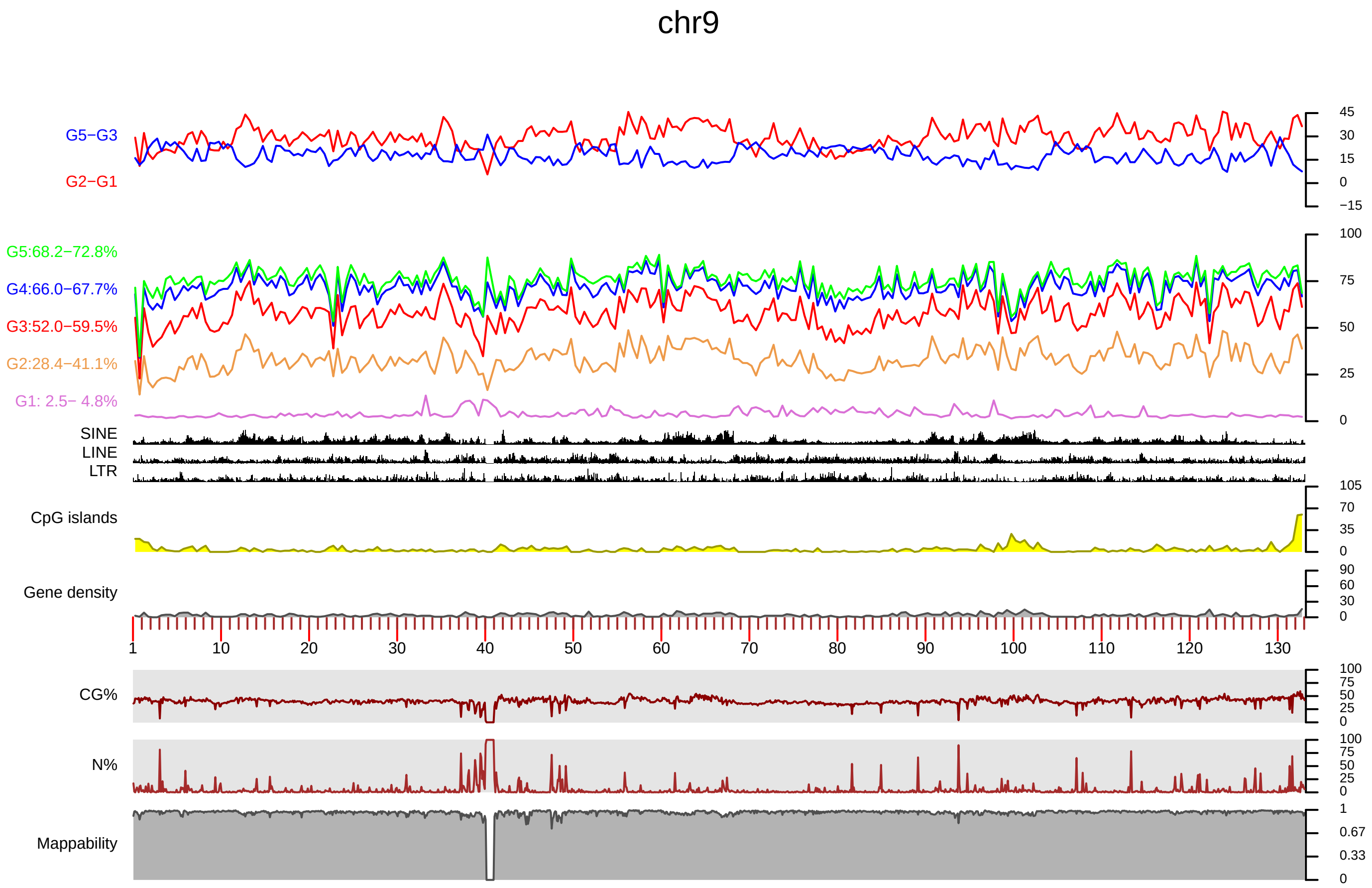
chr9
G5−G3
G2−G1
45
30
15
0
−15
G5:68.2−72.8%
G4:66.0−67.7%
G3:52.0−59.5%
G2:28.4−41.1%
G1: 2.5− 4.8%
100
75
50
25
0
SINE
LINE
LTR
CpG islands
105
70
35
0
Gene density
90
60
30
0
1
10
20
30
40
50
60
70
80
90
100
110
120
130
CG%
100
75
50
25
0
N%
100
75
50
25
0
Mappability
1
0.67
0.33
0

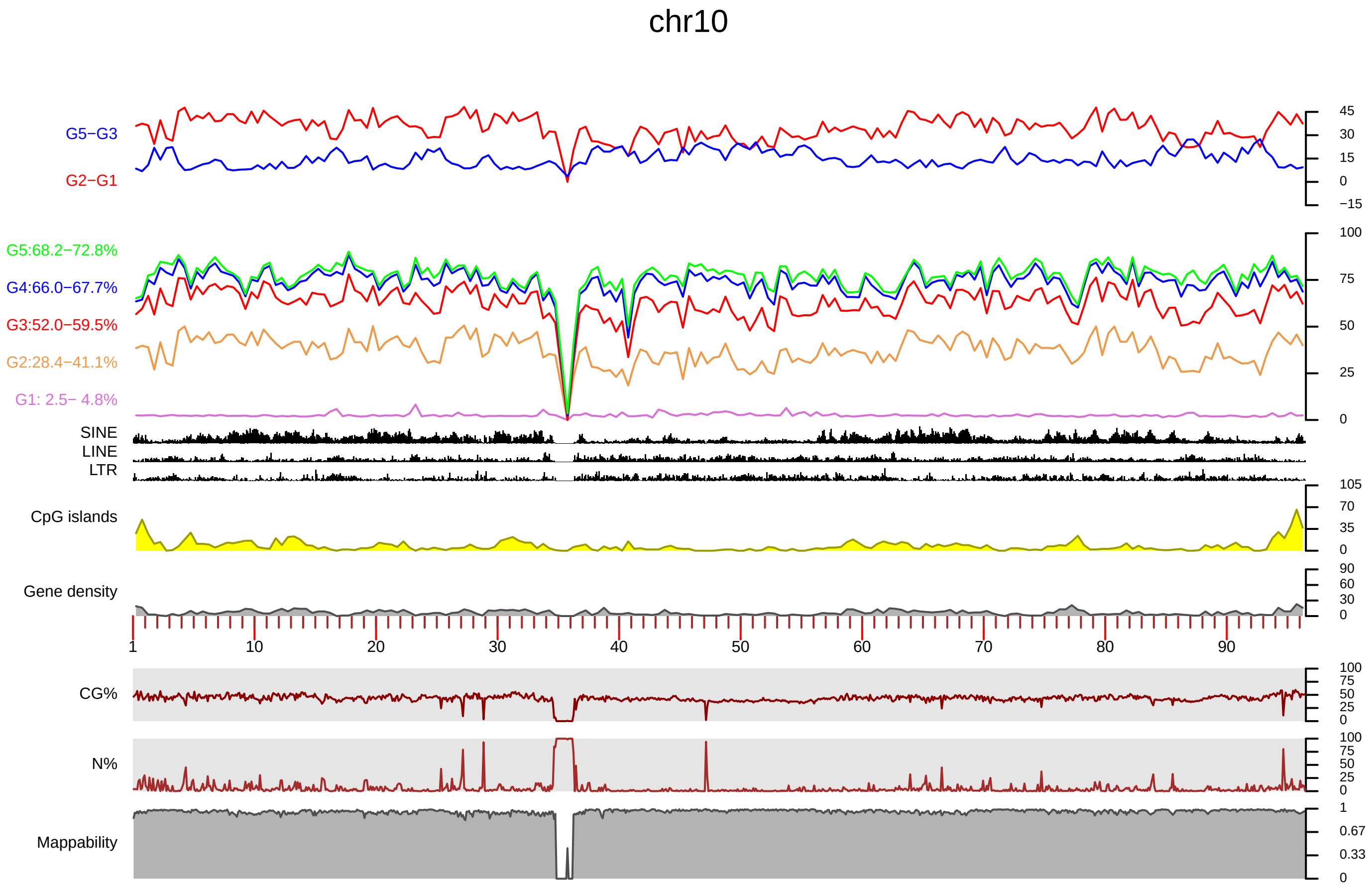
chr10
G5−G3
G2−G1
45
30
15
0
−15
G5:68.2−72.8%
G4:66.0−67.7%
G3:52.0−59.5%
G2:28.4−41.1%
G1: 2.5− 4.8%
100
75
50
25
0
SINE
LINE
LTR
CpG islands
105
70
35
0
Gene density
90
60
30
0
1
10
20
30
40
50
60
70
80
90
CG%
100
75
50
25
0
N%
100
75
50
25
0
Mappability
1
0.67
0.33
0

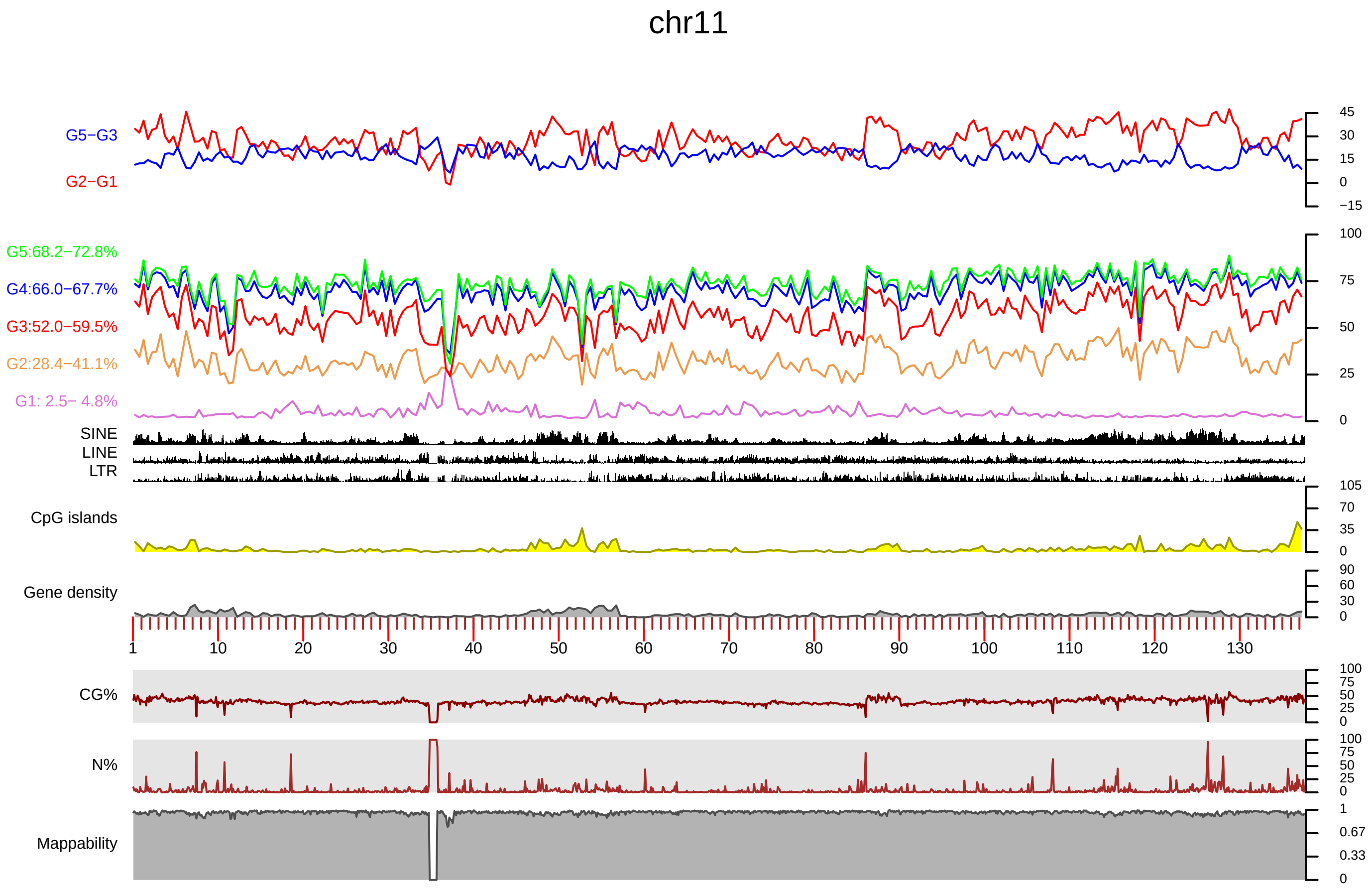
chr11
G5−G3
G2−G1
45
30
15
0
−15
G5:68.2−72.8%
G4:66.0−67.7%
G3:52.0−59.5%
G2:28.4−41.1%
G1: 2.5− 4.8%
100
75
50
25
0
SINE
LINE
LTR
CpG islands
105
70
35
0
Gene density
90
60
30
0
1
10
20
30
40
50
60
70
80
90
100
110
120
130
CG%
100
75
50
25
0
N%
100
75
50
25
0
Mappability
1
0.67
0.33
0

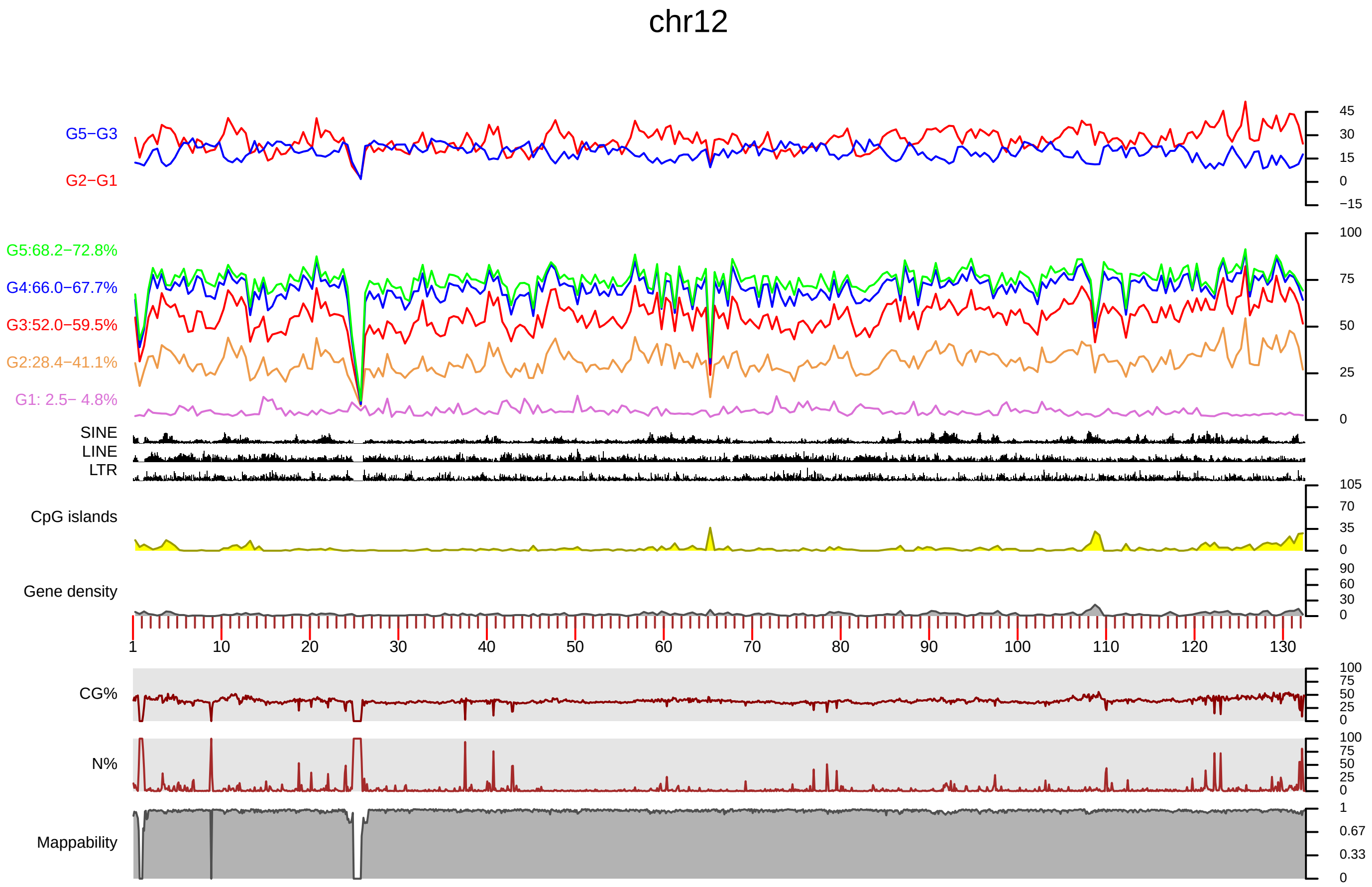
chr12
G5−G3
G2−G1
45
30
15
0
−15
G5:68.2−72.8%
G4:66.0−67.7%
G3:52.0−59.5%
G2:28.4−41.1%
G1: 2.5− 4.8%
100
75
50
25
0
SINE
LINE
LTR
CpG islands
105
70
35
0
Gene density
90
60
30
0
1
10
20
30
40
50
60
70
80
90
100
110
120
130
CG%
100
75
50
25
0
N%
100
75
50
25
0
Mappability
1
0.67
0.33
0

chr13

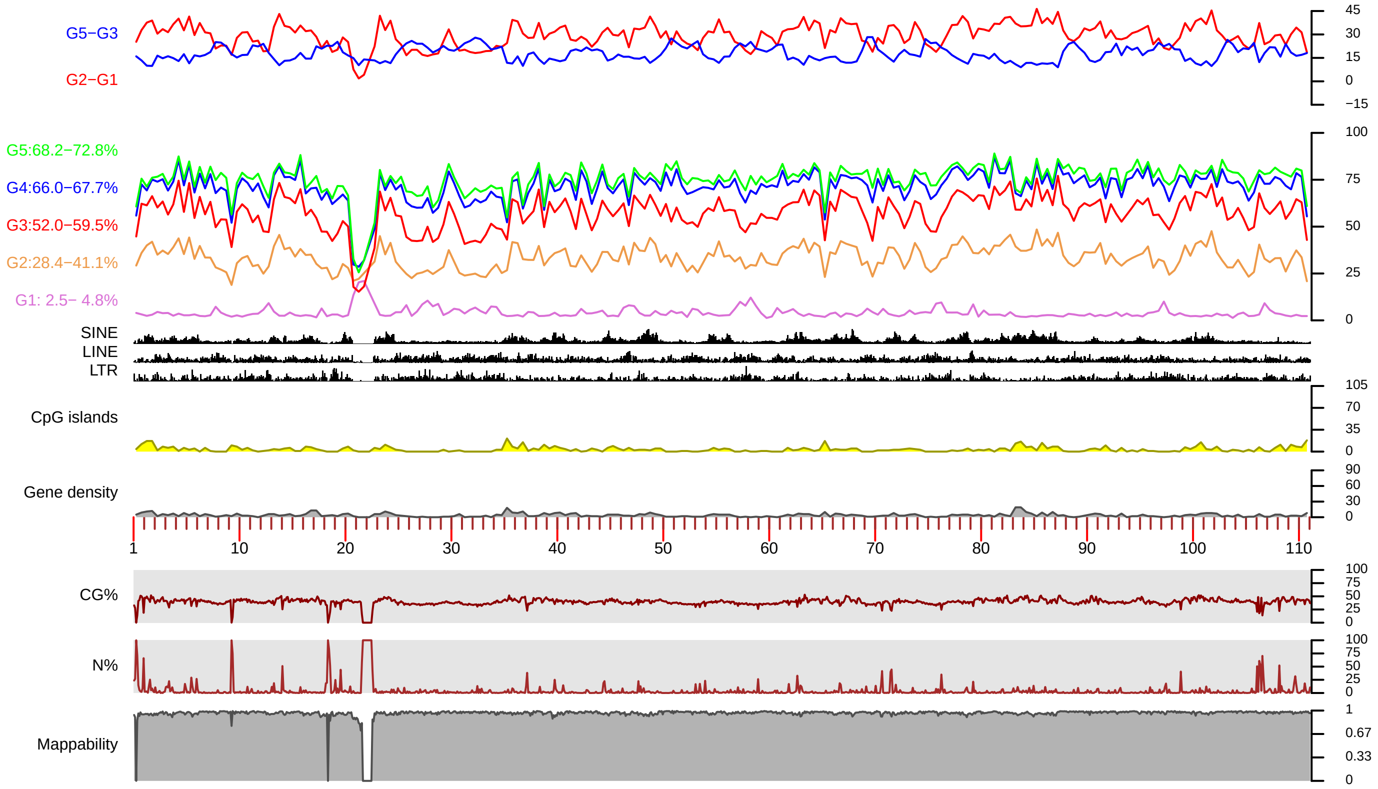

G5−G3
G2−G1
45
30
15
0
−15
G5:68.2−72.8%
G4:66.0−67.7%
G3:52.0−59.5%
G2:28.4−41.1%
G1: 2.5− 4.8%
100
75
50
25
0
SINE
LINE
LTR
CpG islands
105
70
35
0
Gene density
90
60
30
0
1
10
20
30
40
50
60
70
80
90
100
110
CG%
100
75
50
25
0
N%
100
75
50
25
0
Mappability
1
0.67
0.33
0

chr14

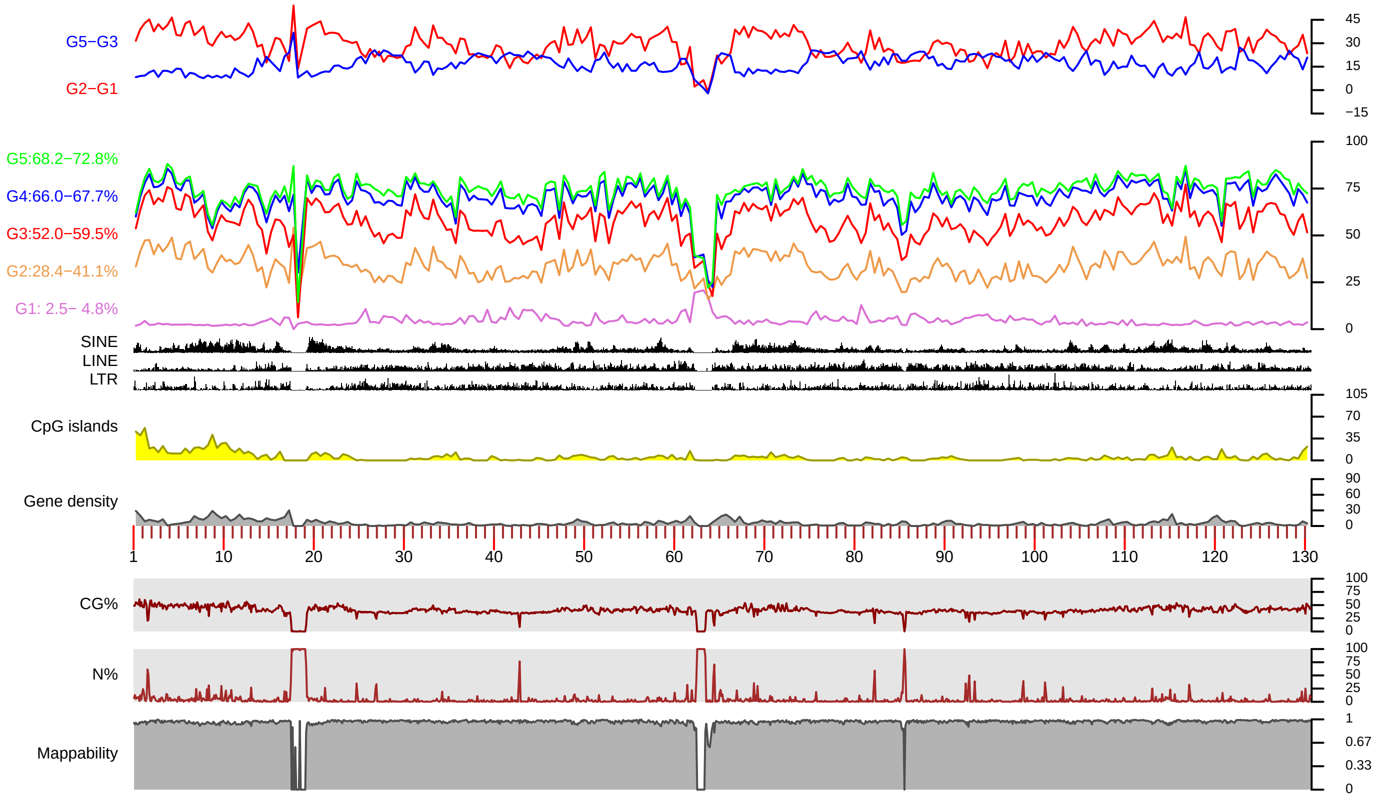

G5−G3
G2−G1
45
30
15
0
−15
G5:68.2−72.8%
G4:66.0−67.7%
G3:52.0−59.5%
G2:28.4−41.1%
G1: 2.5− 4.8%
100
75
50
25
0
SINE
LINE
LTR
CpG islands
105
70
35
0
Gene density
90
60
30
0
1
10
20
30
40
50
60
70
80
90
100
110
120
130
CG%
100
75
50
25
0
N%
100
75
50
25
0
Mappability
1
0.67
0.33
0

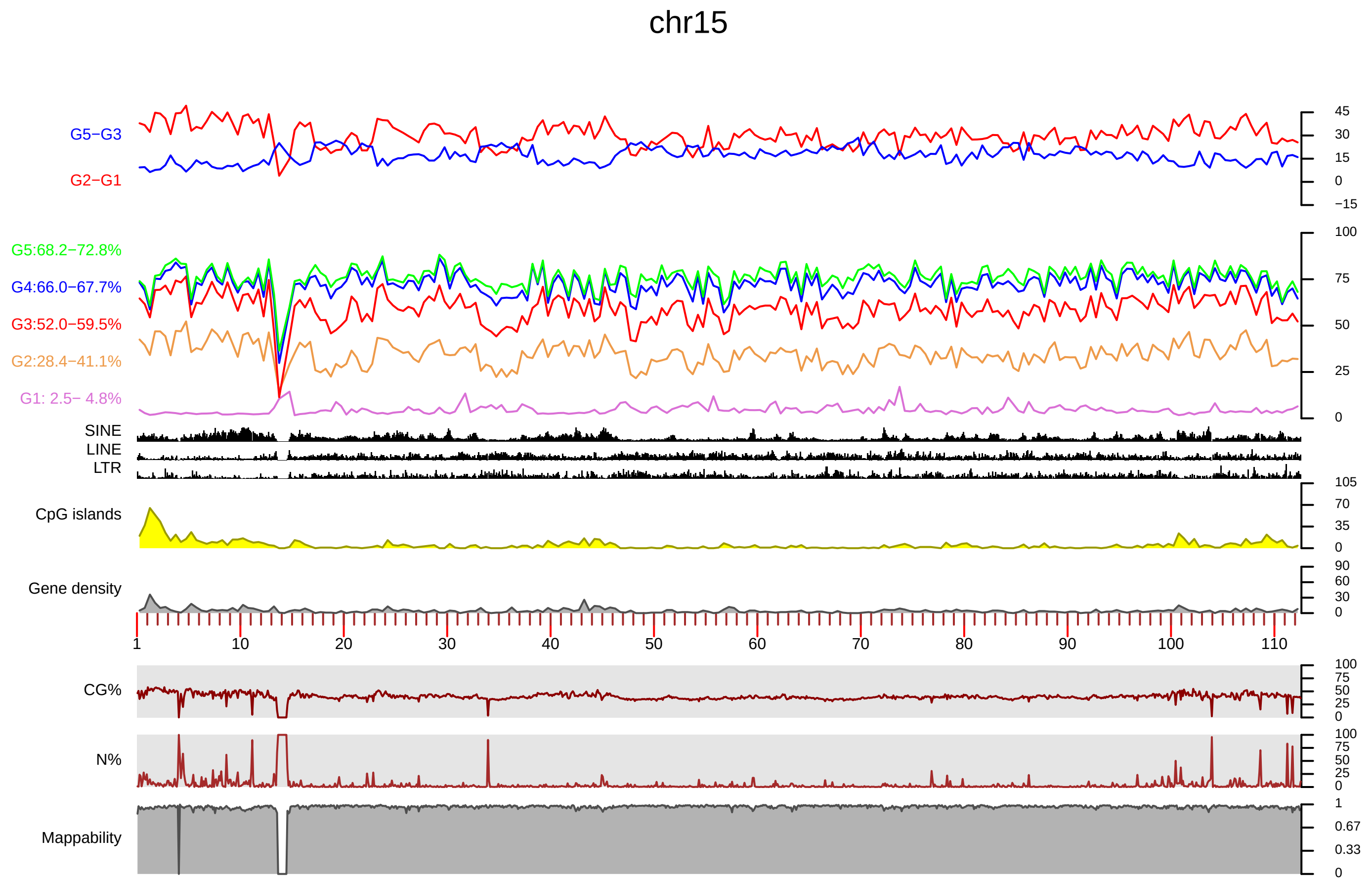
chr15
G5−G3
G2−G1
G5:68.2−72.8%
G4:66.0−67.7%
G3:52.0−59.5%
G2:28.4−41.1%
G1: 2.5− 4.8%
SINE
LINE
LTR
CpG islands
Gene density
CG%
N%
Mappability

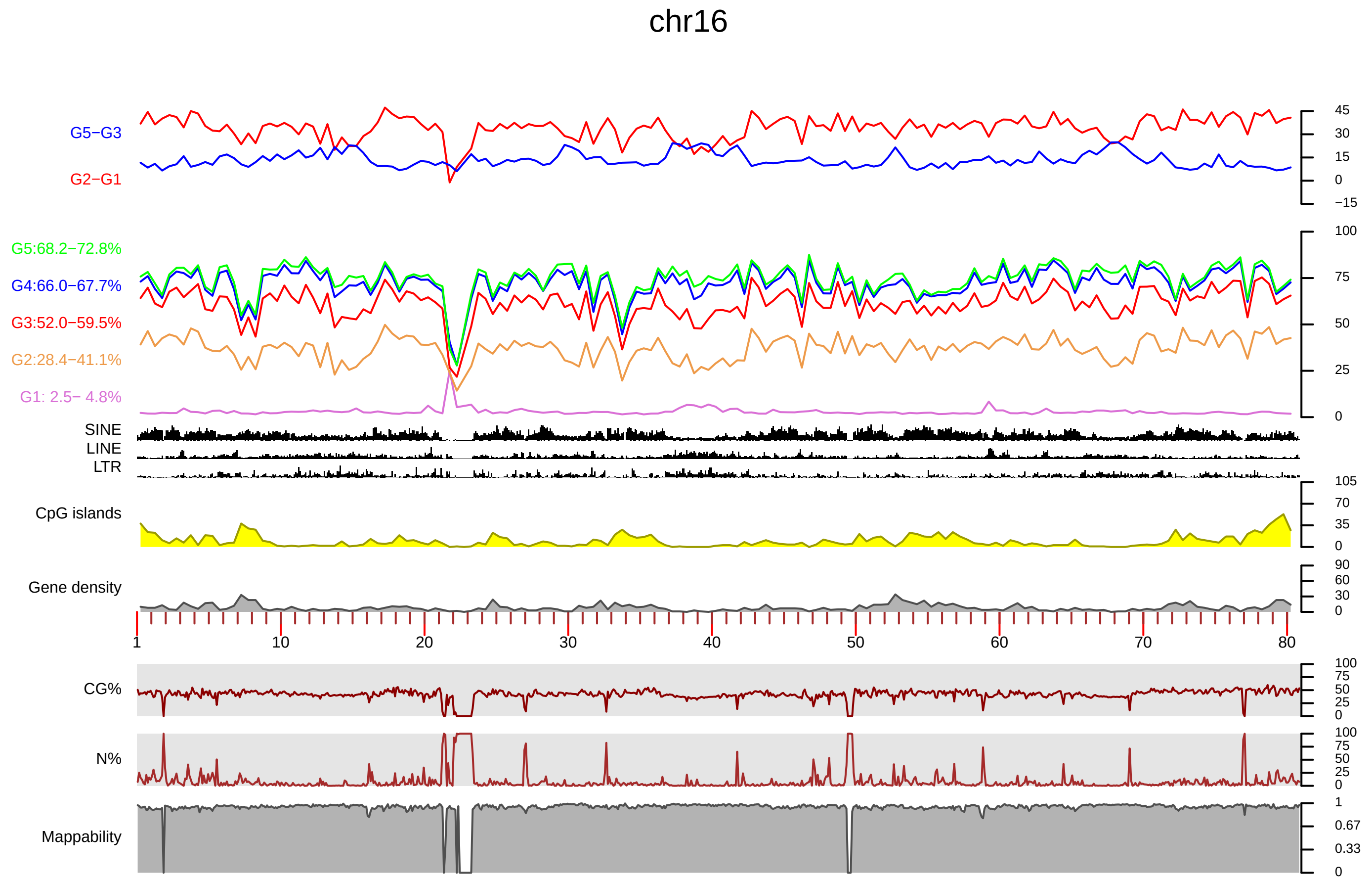
chr16
G5−G3
G2−G1
G5:68.2−72.8%
G4:66.0−67.7%
G3:52.0−59.5%
G2:28.4−41.1%
G1: 2.5− 4.8%
SINE
LINE
LTR
CpG islands
Gene density
CG%
N%
Mappability

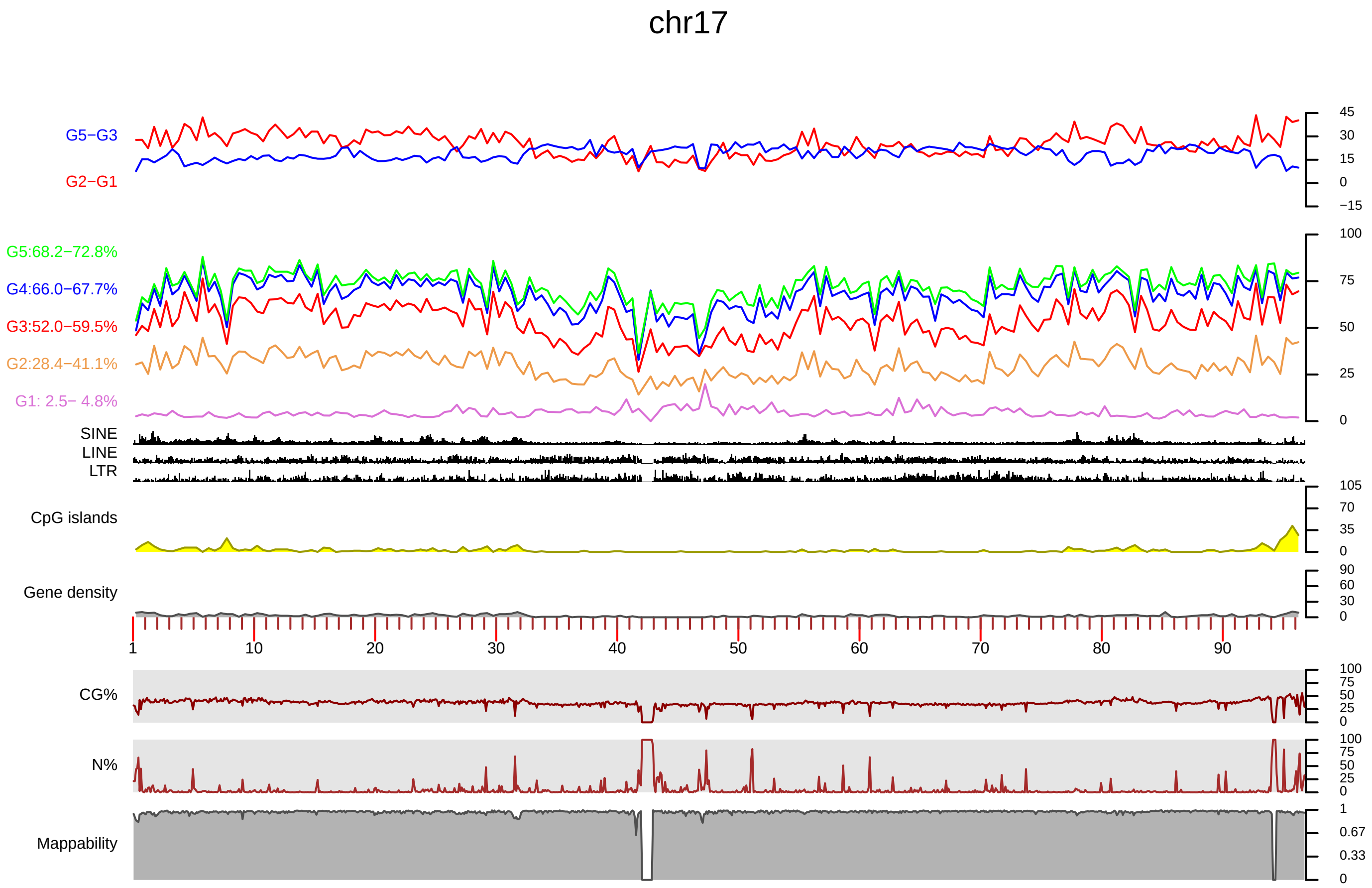
chr17
G5−G3
G2−G1
45
30
15
0
−15
G5:68.2−72.8%
G4:66.0−67.7%
G3:52.0−59.5%
G2:28.4−41.1%
G1: 2.5− 4.8%
100
75
50
25
0
SINE
LINE
LTR
CpG islands
105
70
35
0
Gene density
90
60
30
0
1
10
20
30
40
50
60
70
80
90
CG%
100
75
50
25
0
N%
100
75
50
25
0
Mappability
1
0.67
0.33
0

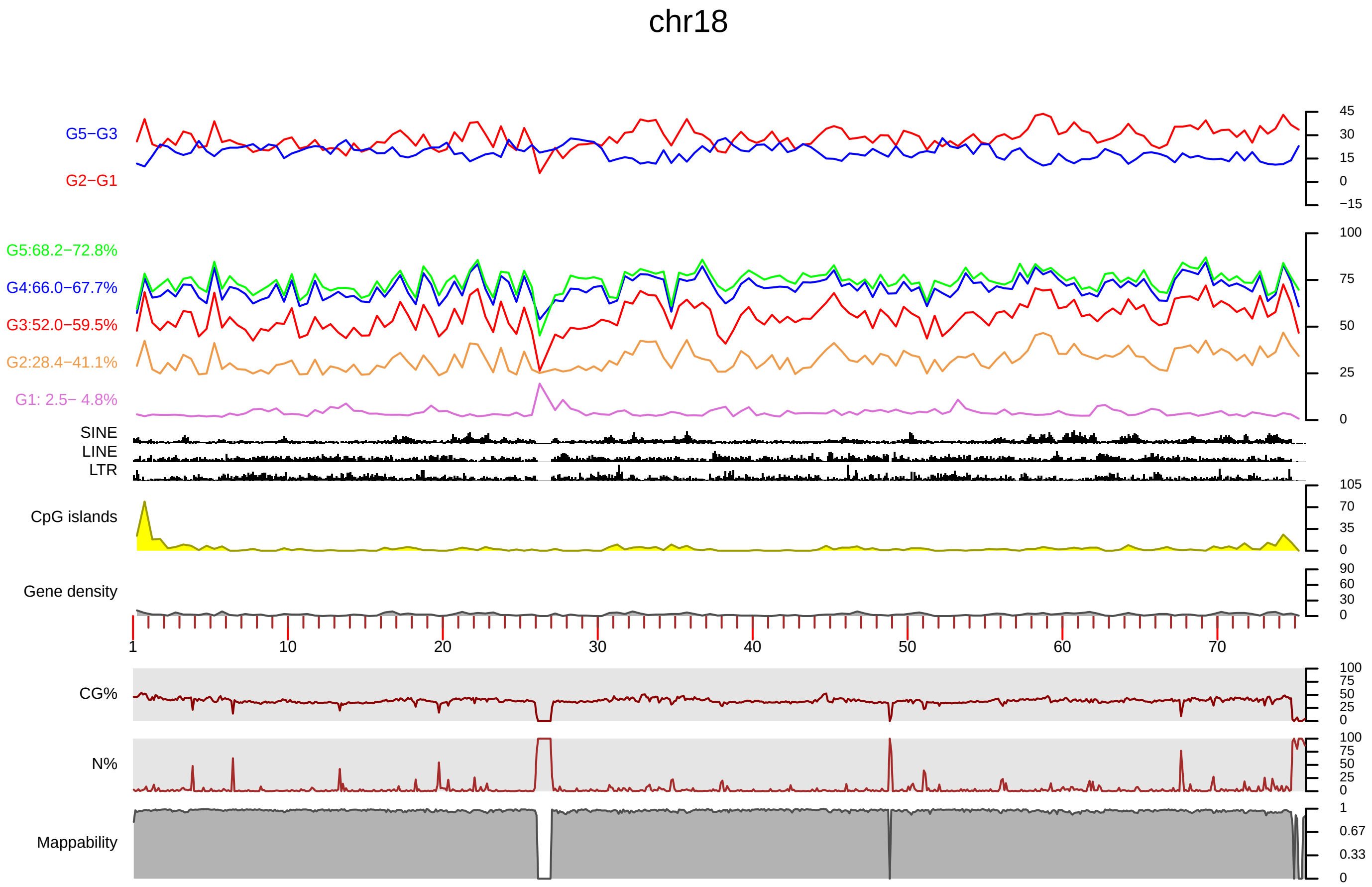
chr18
G5−G3
G2−G1
45
30
15
0
−15
G5:68.2−72.8%
G4:66.0−67.7%
G3:52.0−59.5%
G2:28.4−41.1%
G1: 2.5− 4.8%
100
75
50
25
0
SINE
LINE
LTR
CpG islands
105
70
35
0
Gene density
90
60
30
0
1
10
20
30
40
50
60
70
CG%
100
75
50
25
0
N%
100
75
50
25
0
Mappability
1
0.67
0.33
0

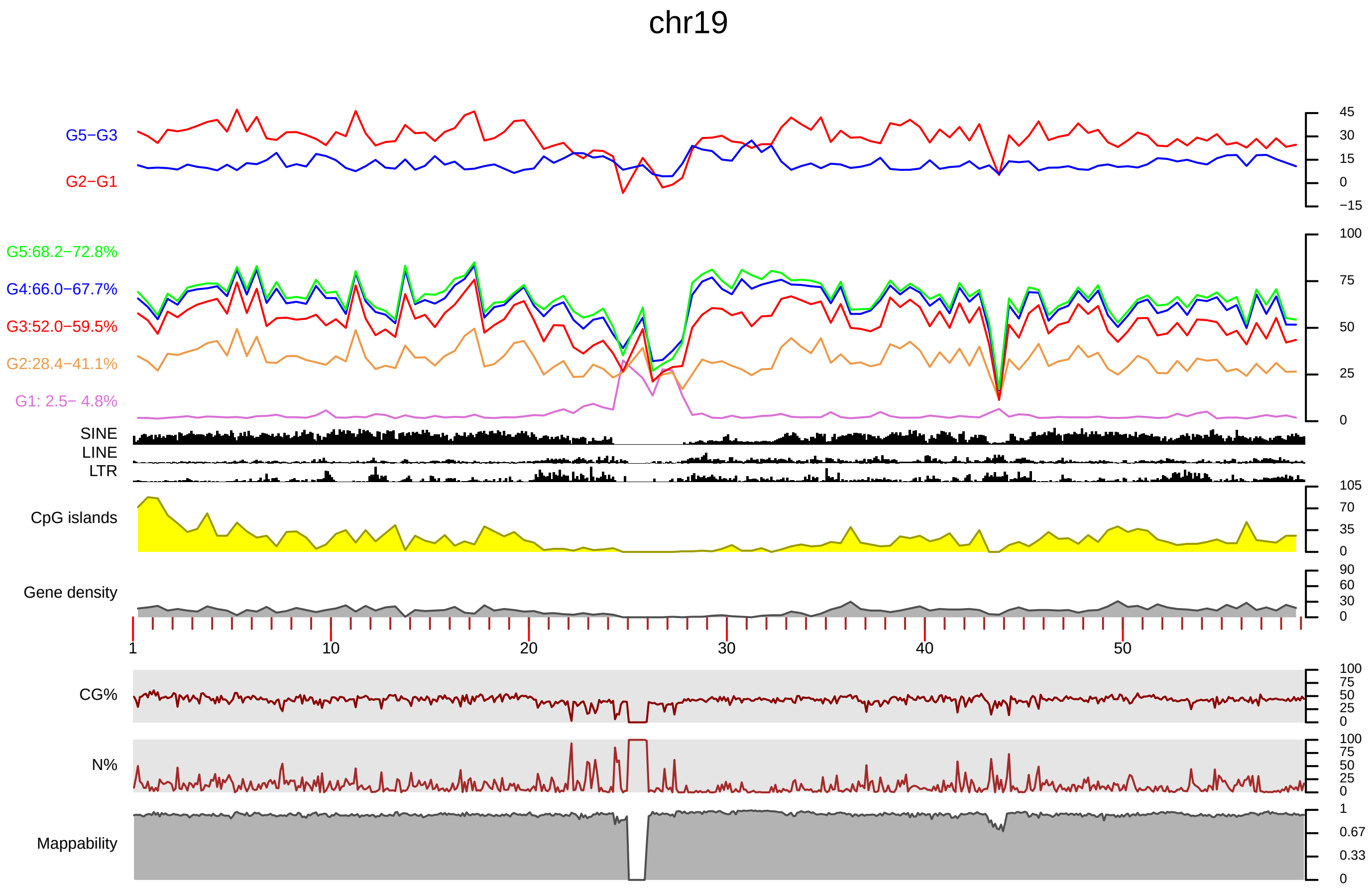
chr19
G5−G3
G2−G1
45
30
15
0
−15
G5:68.2−72.8%
G4:66.0−67.7%
G3:52.0−59.5%
G2:28.4−41.1%
G1: 2.5− 4.8%
100
75
50
25
0
SINE
LINE
LTR
CpG islands
105
70
35
0
Gene density
90
60
30
0
1
10
20
30
40
50
CG%
100
75
50
25
0
N%
100
75
50
25
0
Mappability
1
0.67
0.33
0

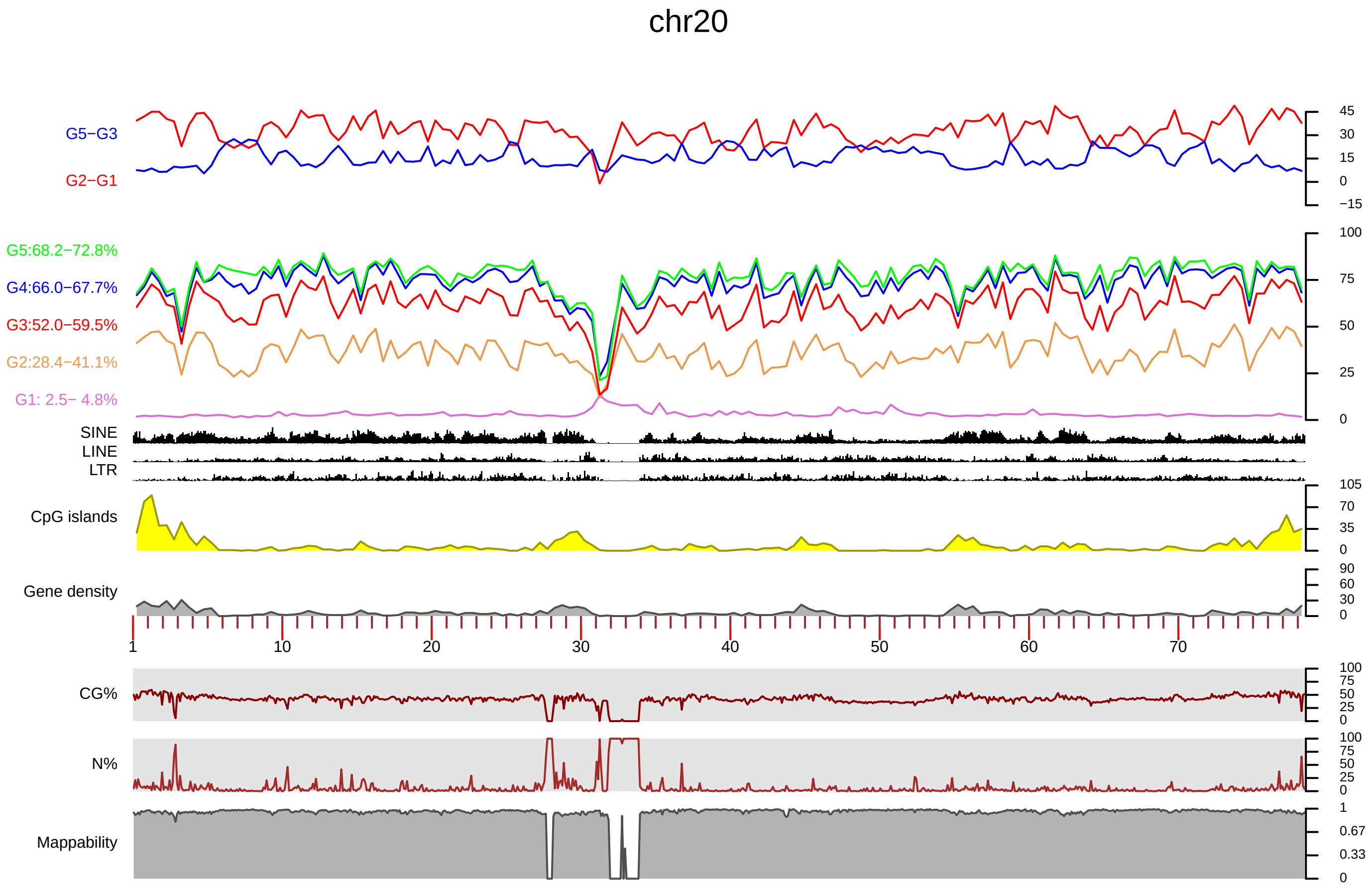
chr20
G5−G3
G2−G1
45
30
15
0
−15
G5:68.2−72.8%
G4:66.0−67.7%
G3:52.0−59.5%
G2:28.4−41.1%
G1: 2.5− 4.8%
100
75
50
25
0
SINE
LINE
LTR
CpG islands
105
70
35
0
Gene density
90
60
30
0
1
10
20
30
40
50
60
70
CG%
100
75
50
25
0
N%
100
75
50
25
0
Mappability
1
0.67
0.33
0

chrX

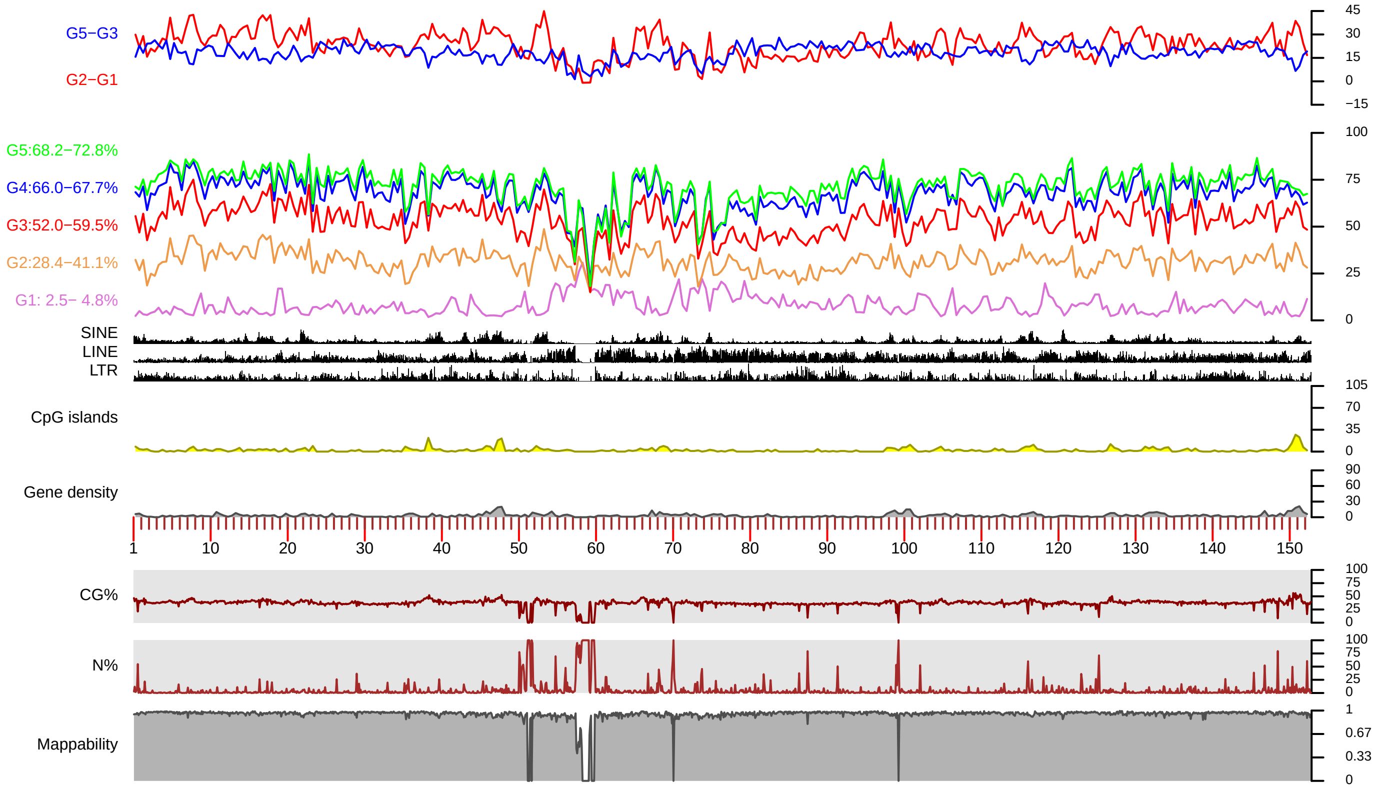
G5−G3
G2−G1
45
30
15
0
−15
G5:68.2−72.8%
G4:66.0−67.7%
G3:52.0−59.5%
G2:28.4−41.1%
G1: 2.5− 4.8%
100
75
50
25
0
SINE
LINE
LTR
CpG islands
105
70
35
0
Gene density
90
60
30
0
1
10
20
30
40
50
60
70
80
90
100
110
120
130
140
150
CG%
100
75
50
25
0
N%
100
75
50
25
0
Mappability
1
0.67
0.33
0

Supplementary Figure. 6

6 days after the removal

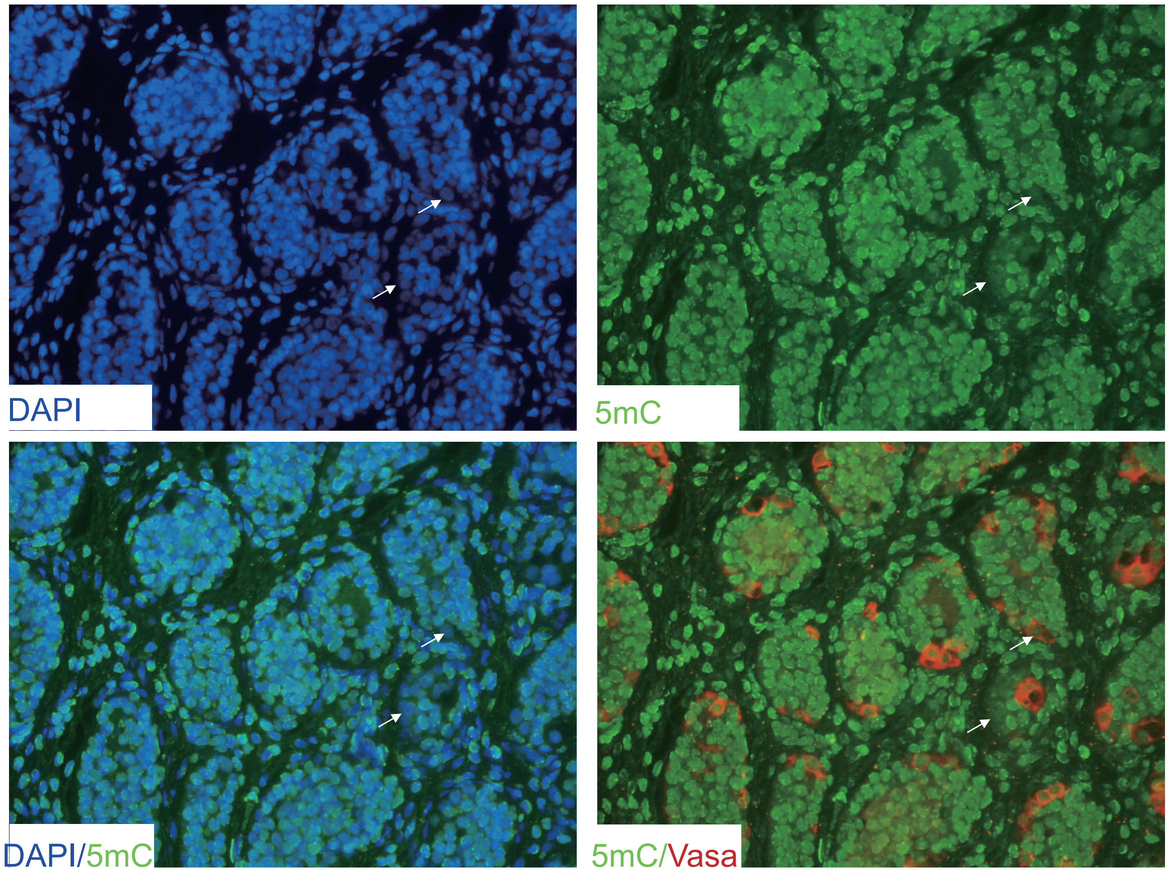

9 months after the removal

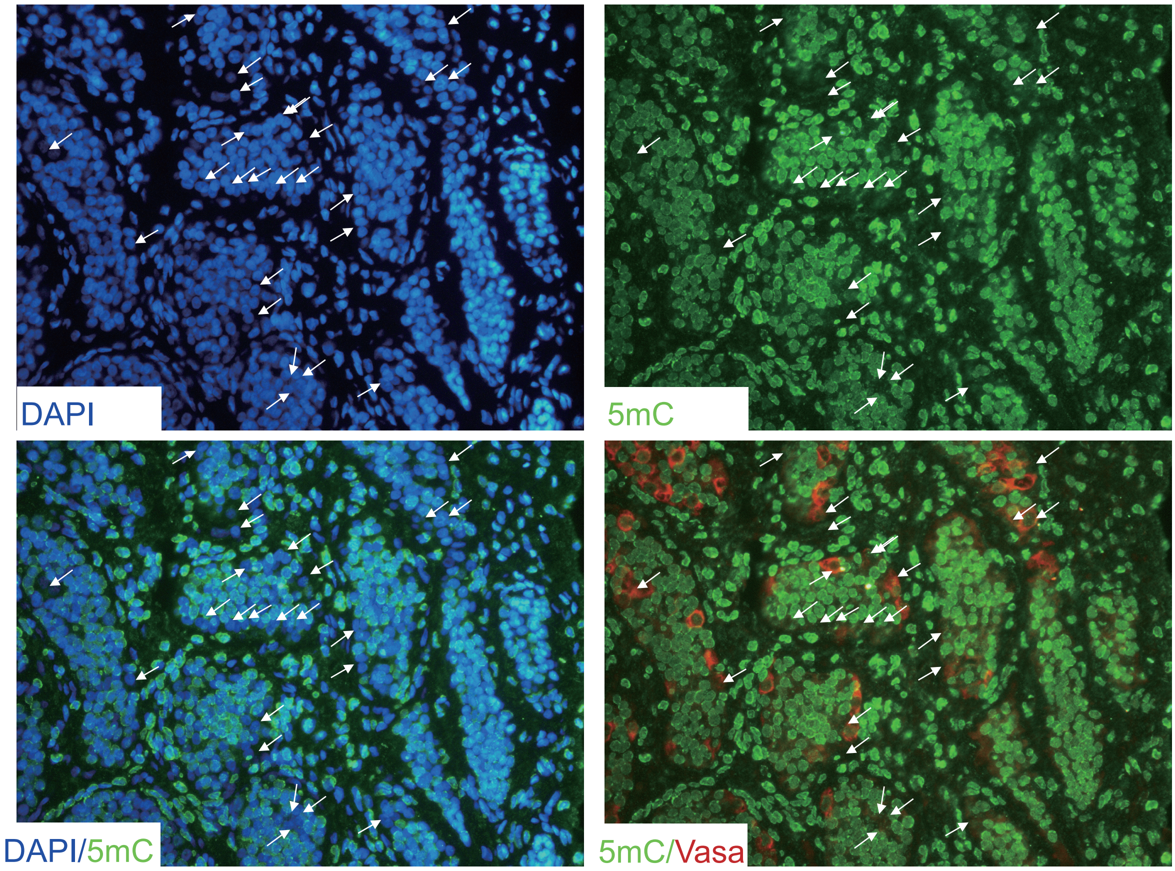

Supplementary Figure 7

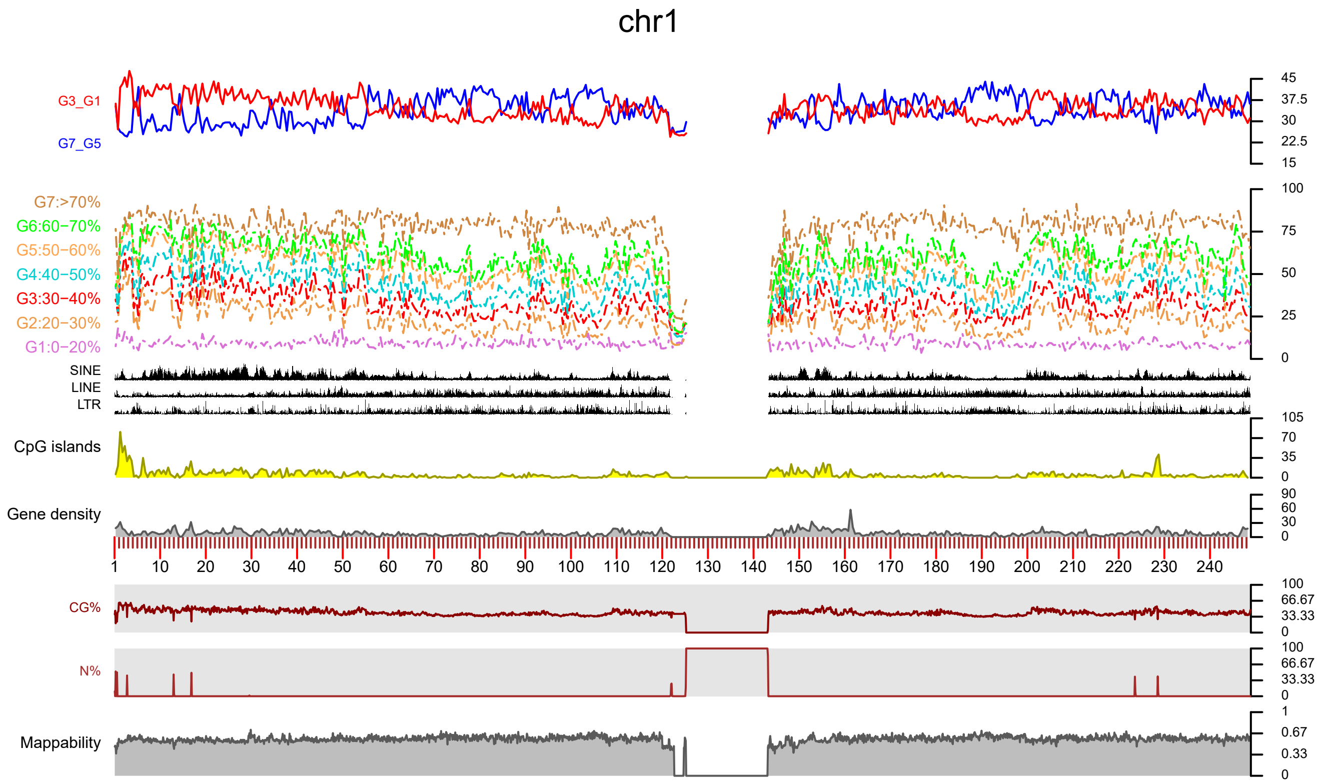

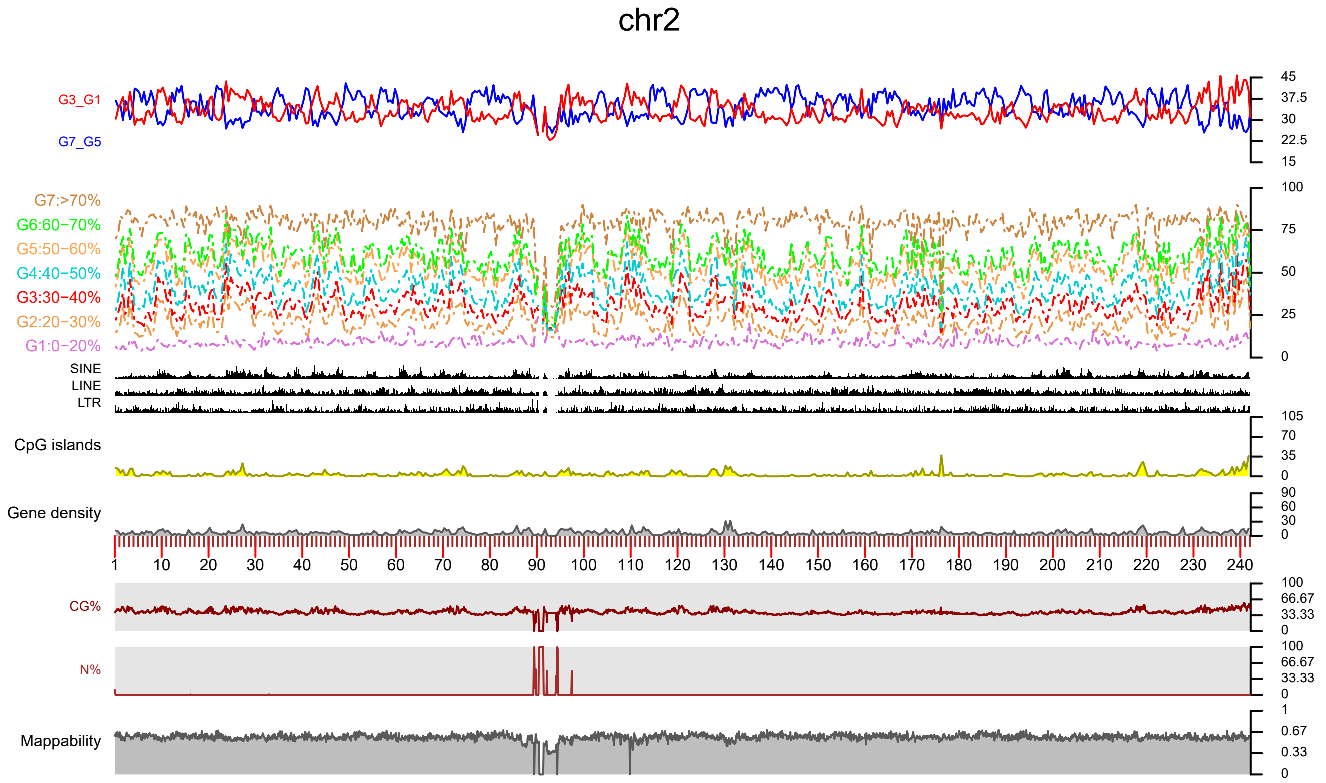

chr3

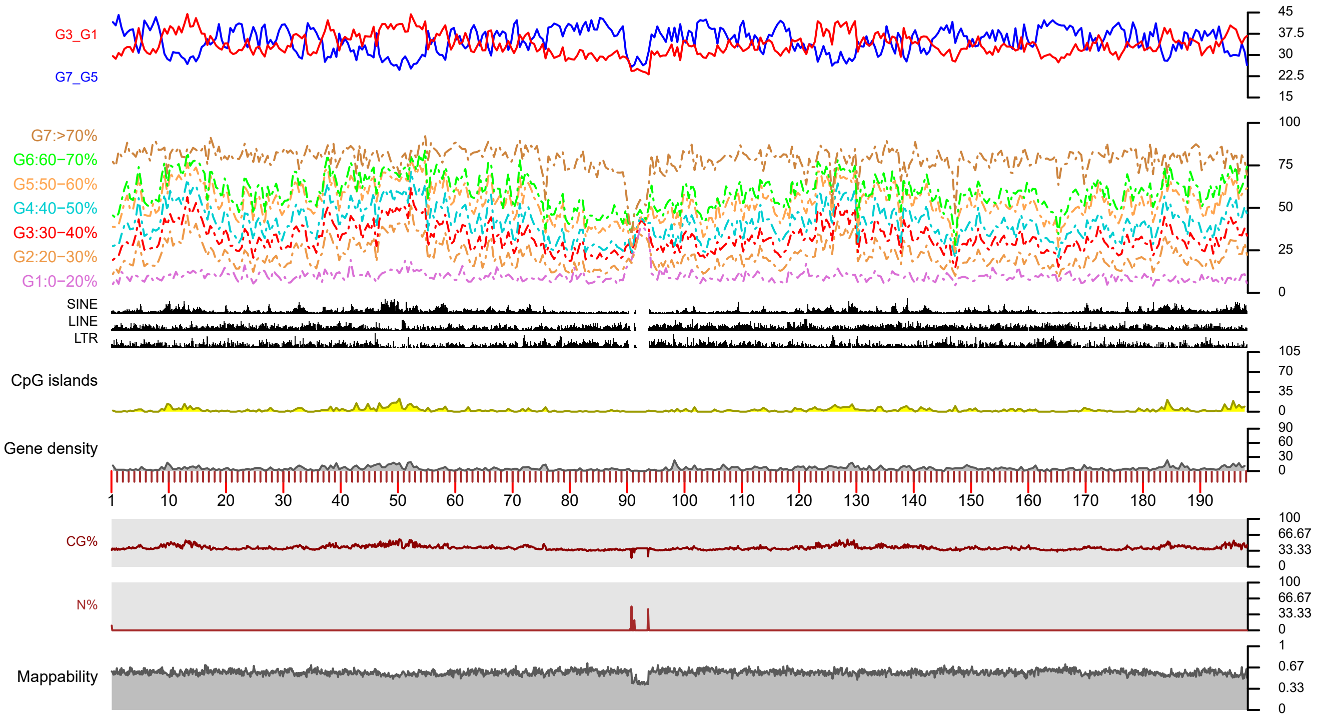
G3_G1
G7_G5
G7:>70%
G6:60−70%
G5:50−60%
G4:40−50%
G3:30−40%
G2:20−30%
G1:0−20%
SINE
LINE
LTR
CpG islands
Gene density
CG%
N%
Mappability

chr4

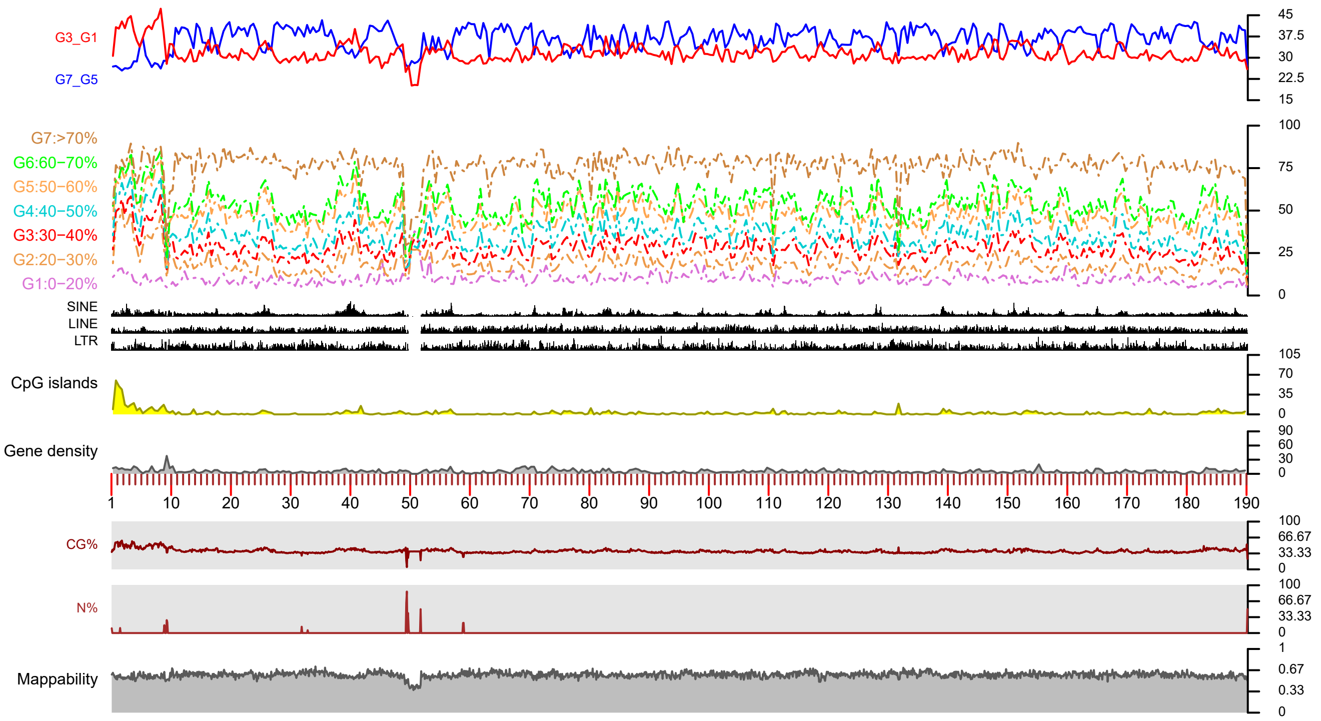
G3_G1
G7_G5
G7:>70%
G6:60−70%
G5:50−60%
G4:40−50%
G3:30−40%
G2:20−30%
G1:0−20%
SINE
LINE
LTR
CpG islands
Gene density
CG%
N%
Mappability

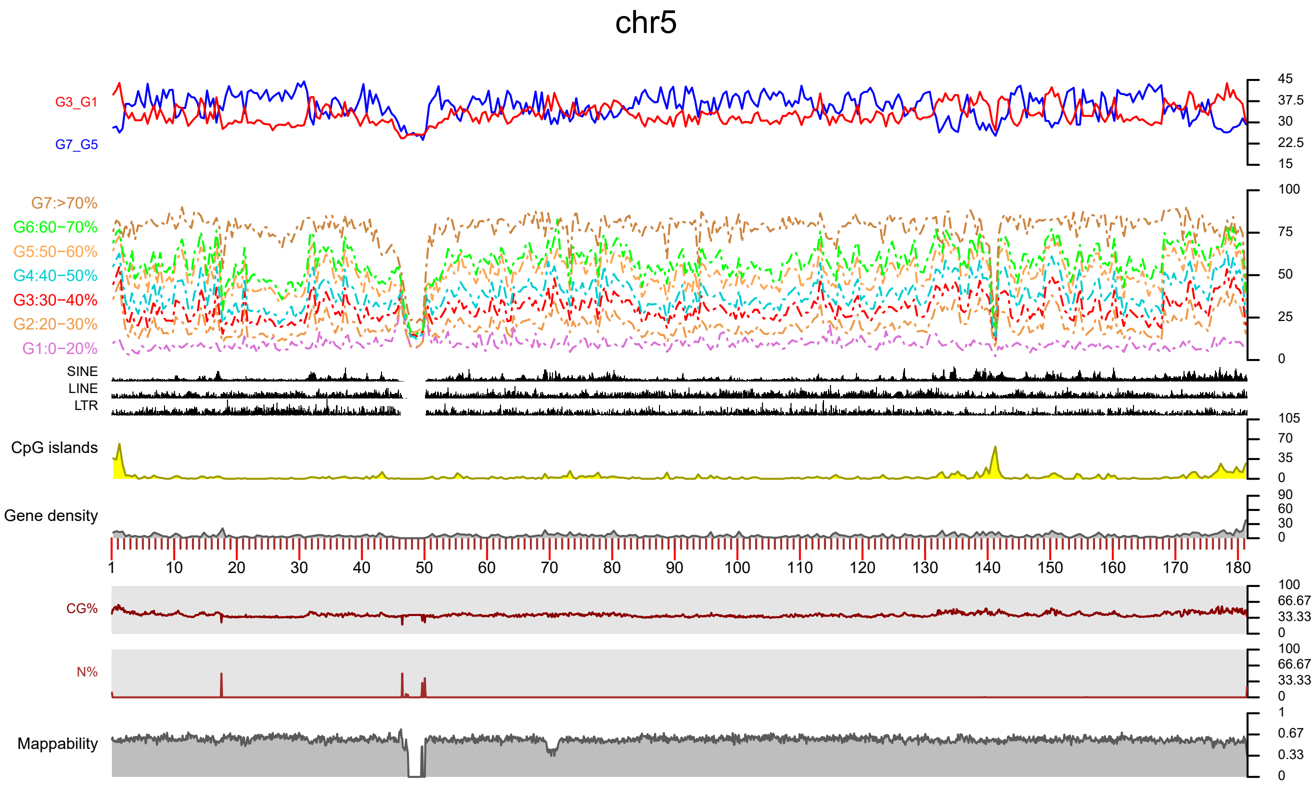
chr5
G3_G1
G7_G5
45
37.5
30
22.5
15
G7:>70%
G6:60−70%
G5:50−60%
G4:40−50%
G3:30−40%
G2:20−30%
G1:0−20%
100
75
50
25
0
SINE
LINE
LTR
CpG islands
105
70
35
0
Gene density
90
60
30
0
1 10 20 30 40 50 60 70 80 90 100 110 120 130 140 150 160 170 180
CG%
100
66.67
33.33
0
N%
100
66.67
33.33
0
Mappability
1
0.67
0.33
0

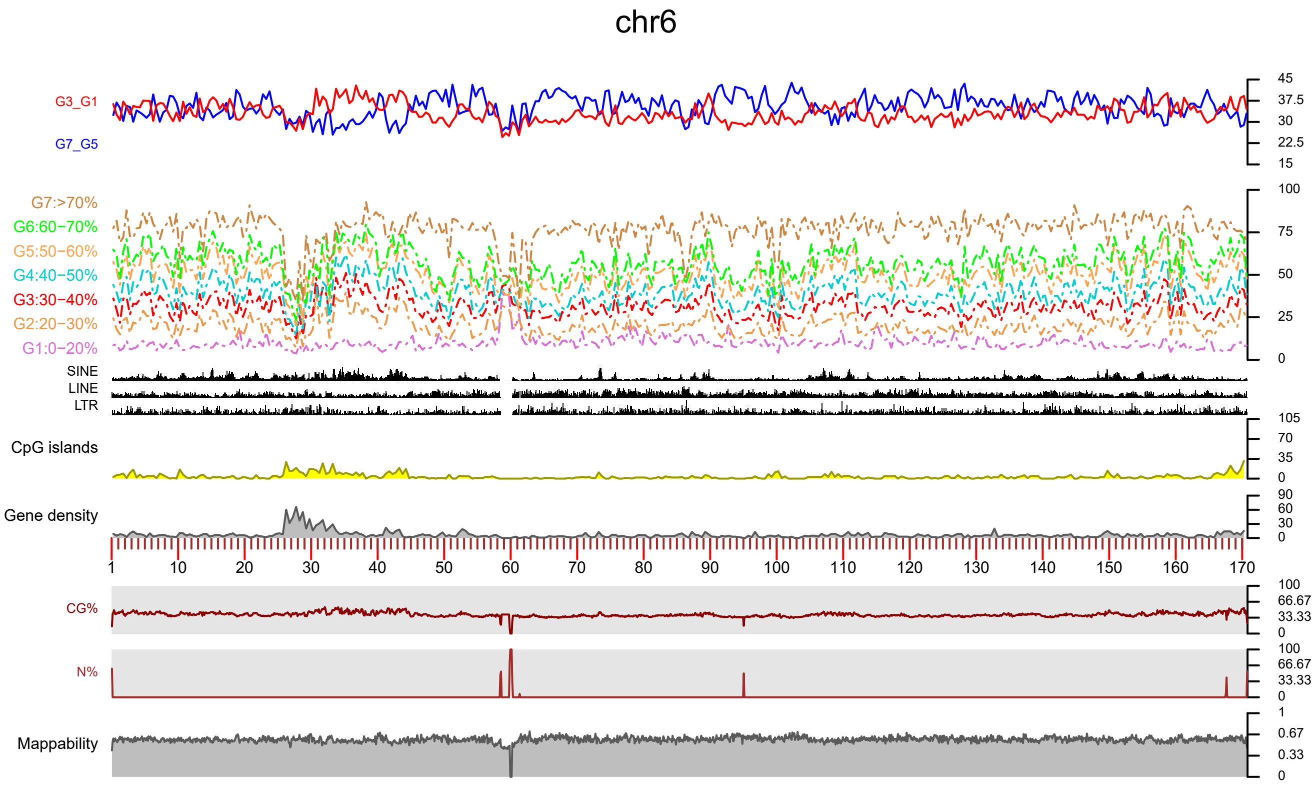
chr6
G3_G1
G7_G5
45
37.5
30
22.5
15
G7:>70%
G6:60−70%
G5:50−60%
G4:40−50%
G3:30−40%
G2:20−30%
G1:0−20%
100
75
50
25
0
SINE
LINE
LTR
CpG islands
105
70
35
0
Gene density
90
60
30
0
1 10 20 30 40 50 60 70 80 90 100 110 120 130 140 150 160 170
CG%
100
66.67
33.33
0
N%
100
66.67
33.33
0
Mappability
1
0.67
0.33
0

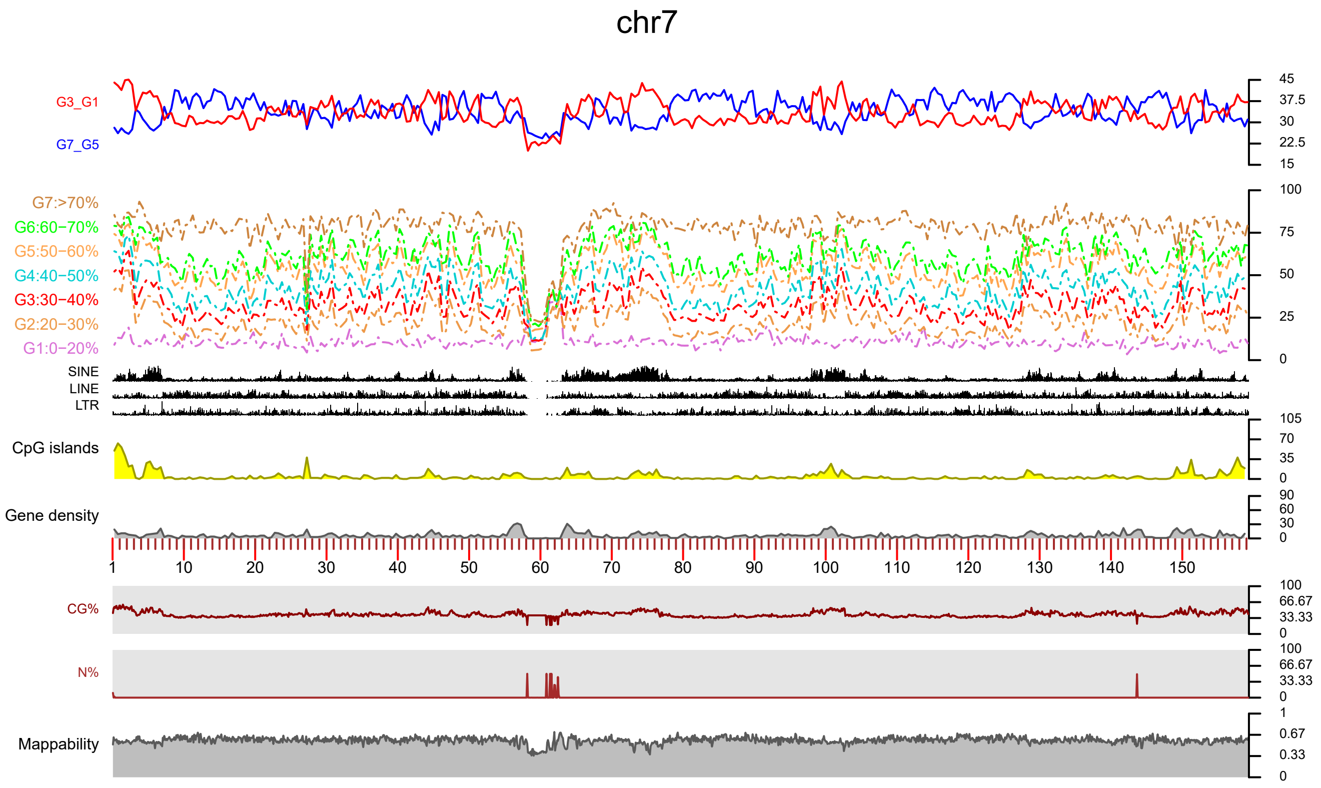
chr7
G3_G1
G7_G5
G7:>70%
G6:60–70%
G5:50–60%
G4:40–50%
G3:30–40%
G2:20–30%
G1:0–20%
SINE
LINE
LTR
CpG islands
Gene density
CG%
N%
Mappability

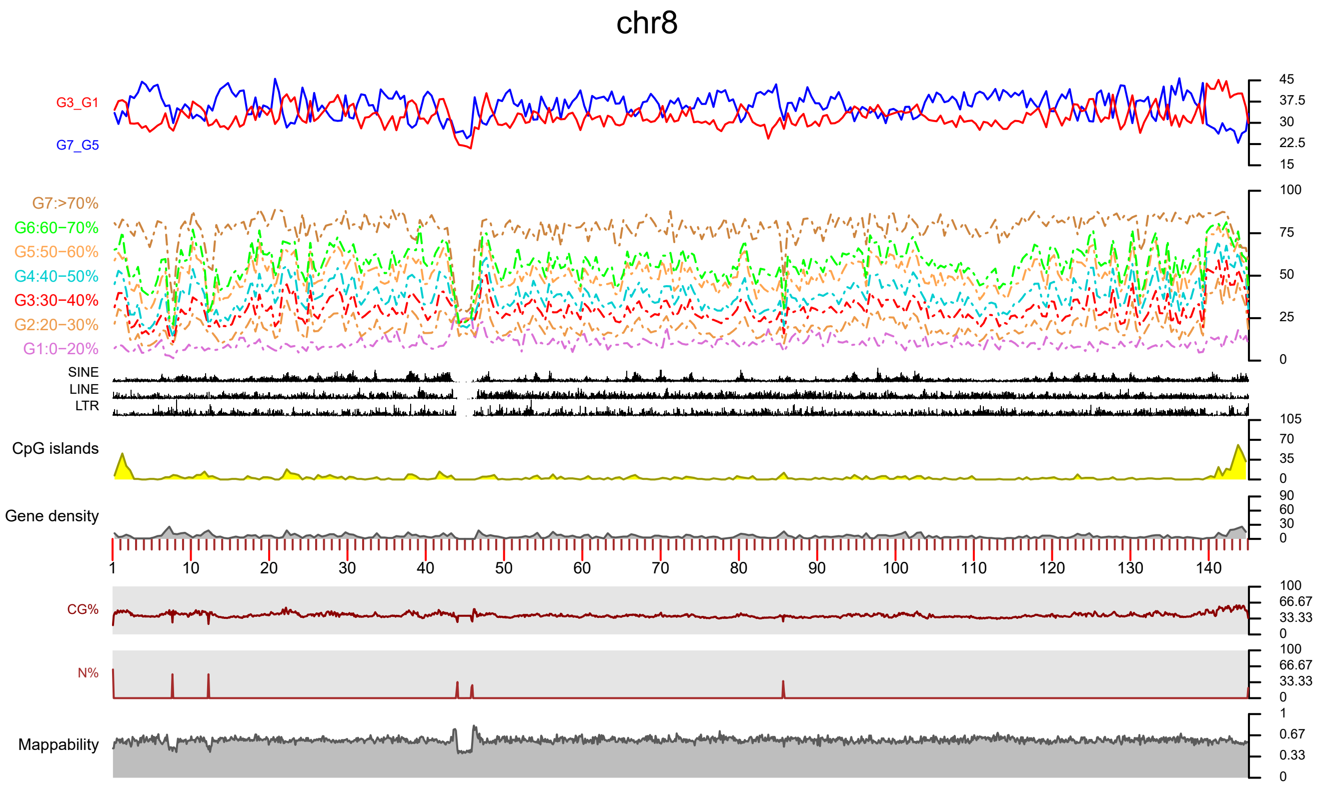
chr8
G3_G1
G7_G5
G7:>70%
G6:60–70%
G5:50–60%
G4:40–50%
G3:30–40%
G2:20–30%
G1:0–20%
SINE
LINE
LTR
CpG islands
Gene density
CG%
N%
Mappability

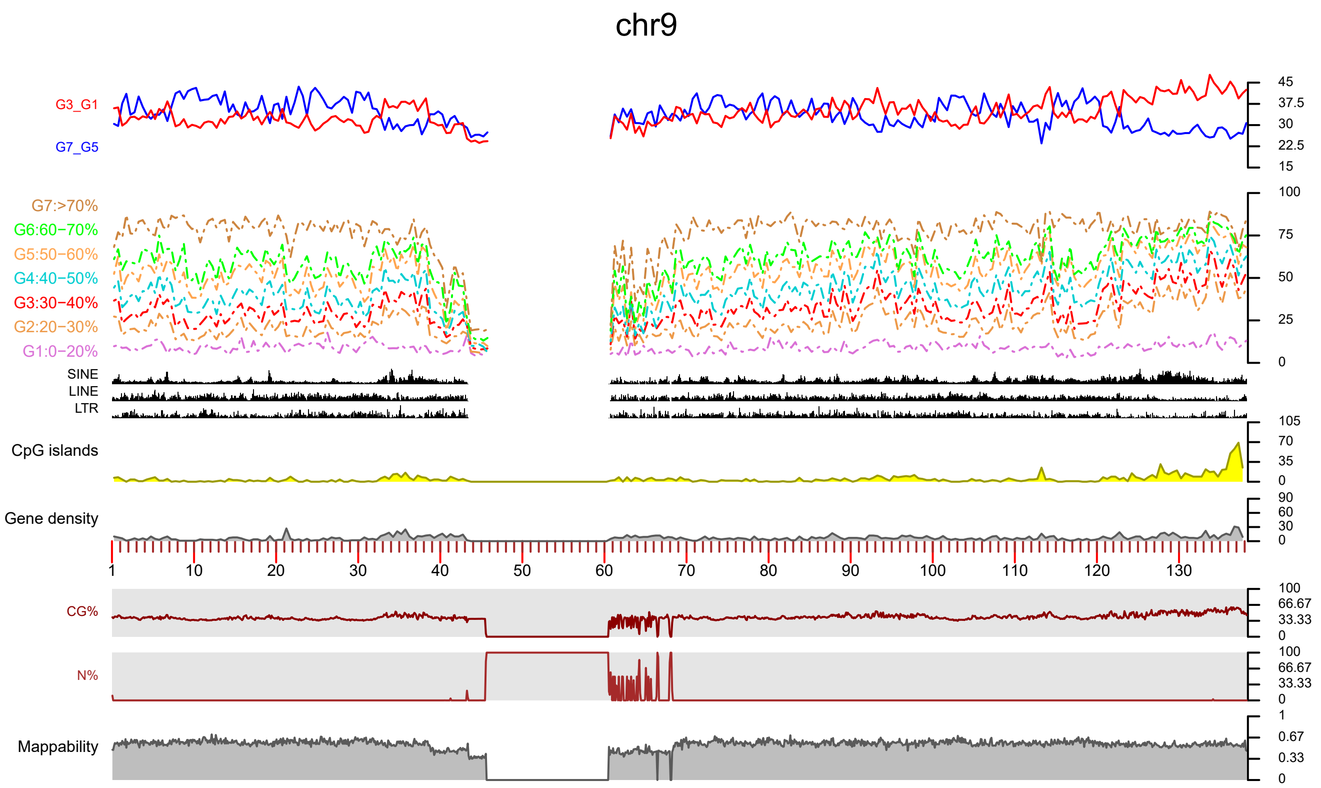
chr9
G3_G1
G7_G5
45
37.5
30
22.5
15
G7:>70%
G6:60−70%
G5:50−60%
G4:40−50%
G3:30−40%
G2:20−30%
G1:0−20%
100
75
50
25
0
SINE
LINE
LTR
CpG islands
105
70
35
0
Gene density
90
60
30
0
1
10
20
30
40
50
60
70
80
90
100
110
120
130
CG%
100
66.67
33.33
0
N%
100
66.67
33.33
0
Mappability
1
0.67
0.33
0

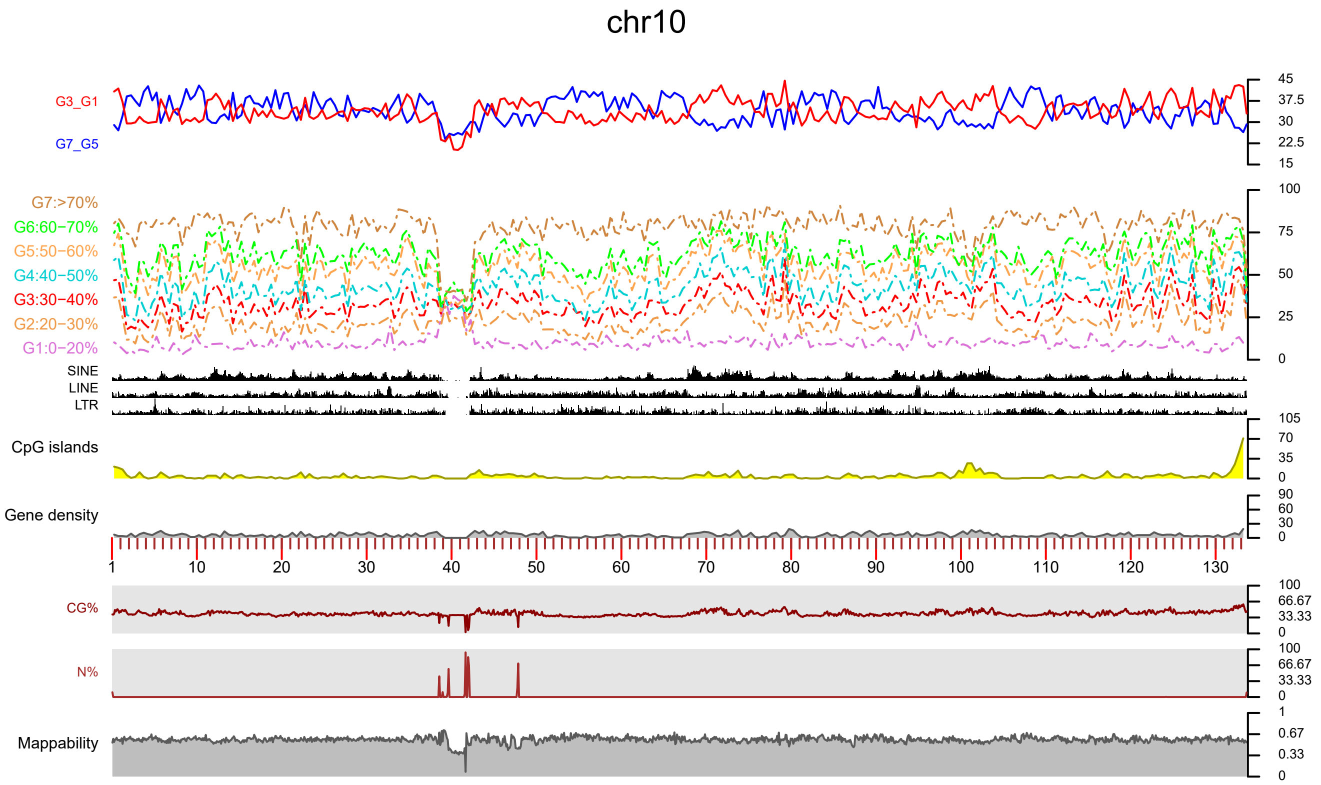
chr10
G3_G1
G7_G5
45
37.5
30
22.5
15
G7:>70%
G6:60−70%
G5:50−60%
G4:40−50%
G3:30−40%
G2:20−30%
G1:0−20%
100
75
50
25
0
SINE
LINE
LTR
CpG islands
105
70
35
0
Gene density
90
60
30
0
1
10
20
30
40
50
60
70
80
90
100
110
120
130
CG%
100
66.67
33.33
0
N%
100
66.67
33.33
0
Mappability
1
0.67
0.33
0

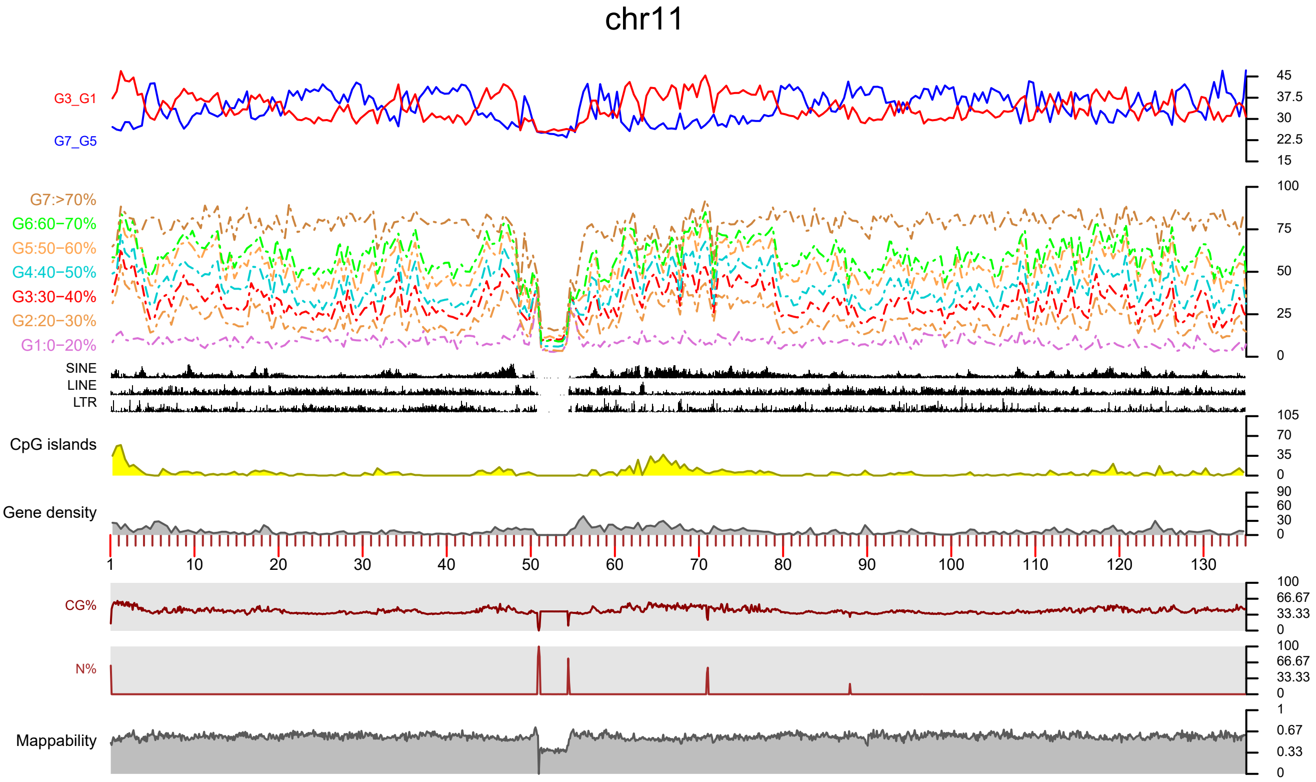
chr11
G3_G1
G7_G5
45
37.5
30
22.5
15
G7:>70%
G6:60–70%
G5:50–60%
G4:40–50%
G3:30–40%
G2:20–30%
G1:0–20%
100
75
50
25
0
SINE
LINE
LTR
CpG islands
105
70
35
0
Gene density
90
60
30
0
1
10
20
30
40
50
60
70
80
90
100
110
120
130
CG%
100
66.67
33.33
0
N%
100
66.67
33.33
0
Mappability
1
0.67
0.33
0

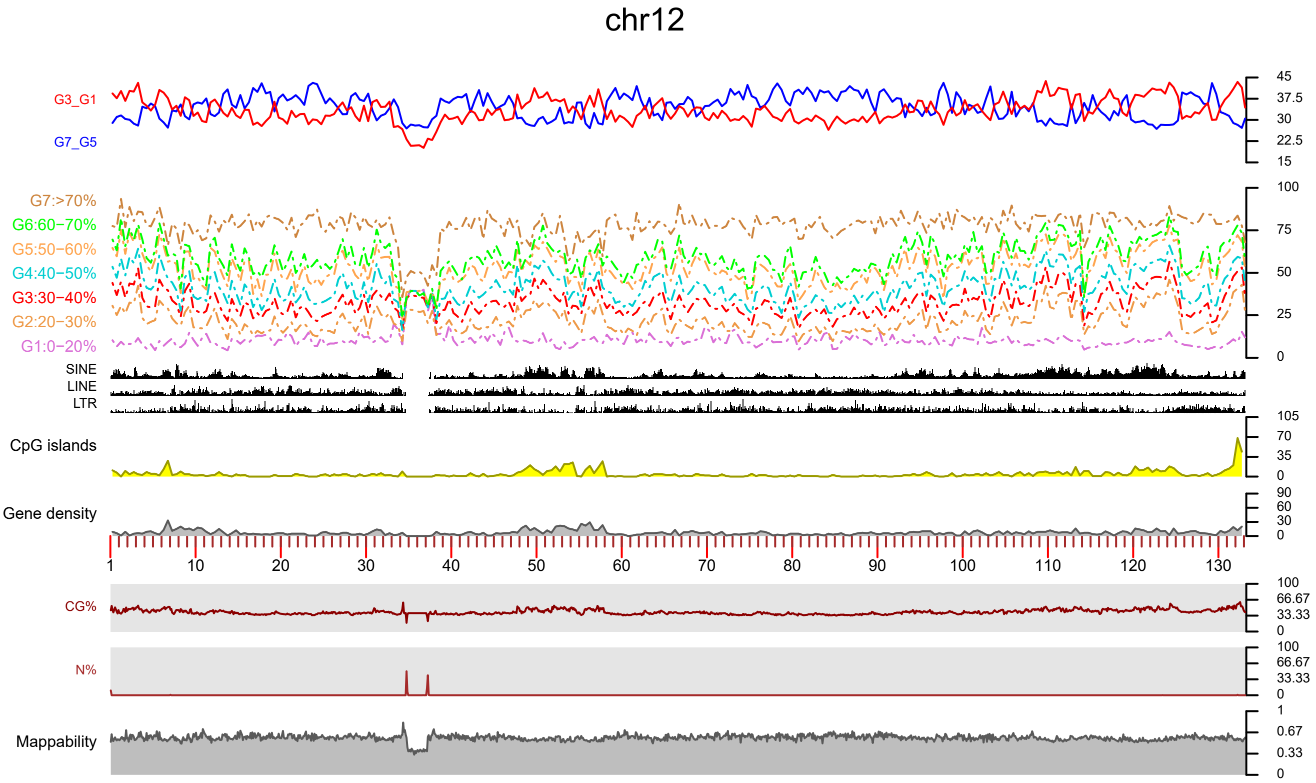
chr12
G3_G1
G7_G5
45
37.5
30
22.5
15
G7:>70%
G6:60–70%
G5:50–60%
G4:40–50%
G3:30–40%
G2:20–30%
G1:0–20%
100
75
50
25
0
SINE
LINE
LTR
CpG islands
105
70
35
0
Gene density
90
60
30
0
1
10
20
30
40
50
60
70
80
90
100
110
120
130
CG%
100
66.67
33.33
0
N%
100
66.67
33.33
0
Mappability
1
0.67
0.33
0

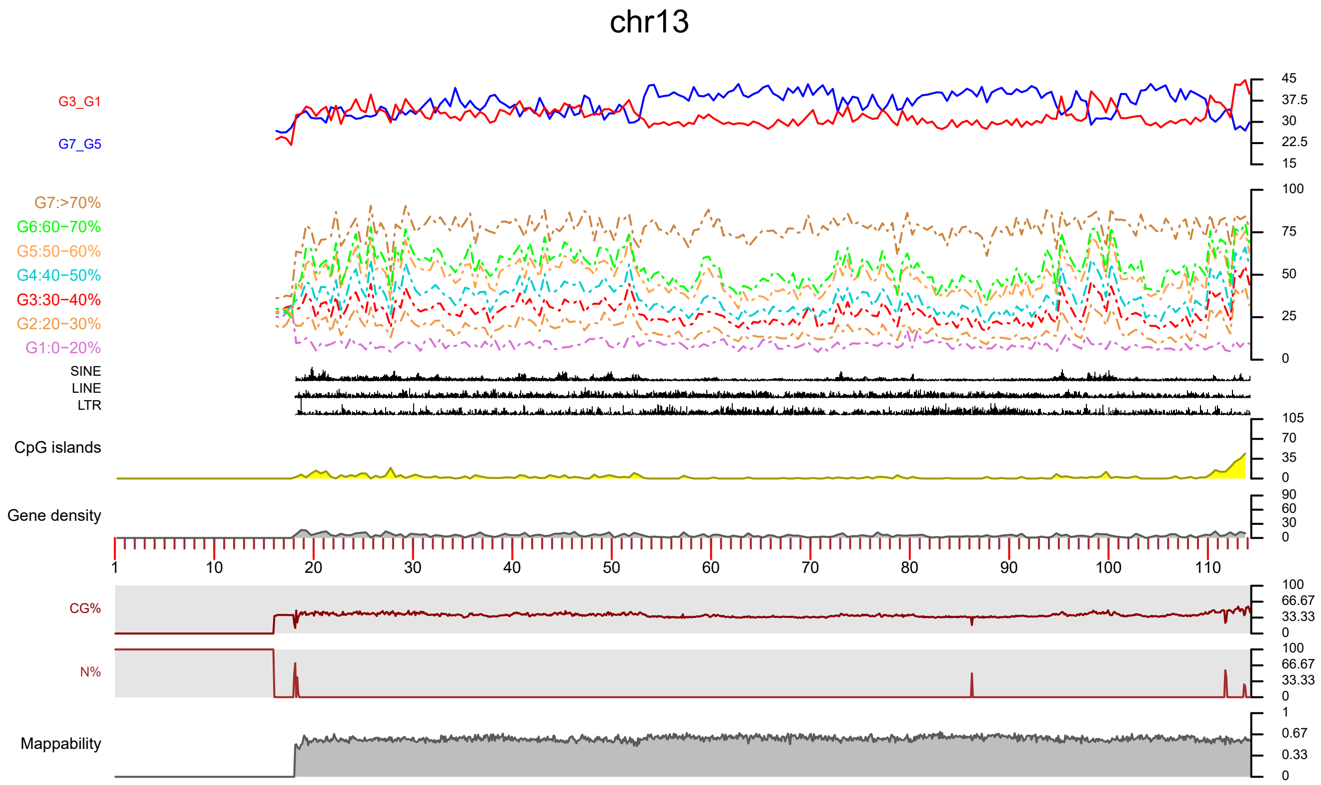
chr13
G3_G1
G7_G5
45
37.5
30
22.5
15
G7:>70%
G6:60–70%
G5:50–60%
G4:40–50%
G3:30–40%
G2:20–30%
G1:0–20%
100
75
50
25
0
SINE
LINE
LTR
CpG islands
105
70
35
0
Gene density
90
60
30
0
1
10
20
30
40
50
60
70
80
90
100
110
CG%
100
66.67
33.33
0
N%
100
66.67
33.33
0
Mappability
1
0.67
0.33
0

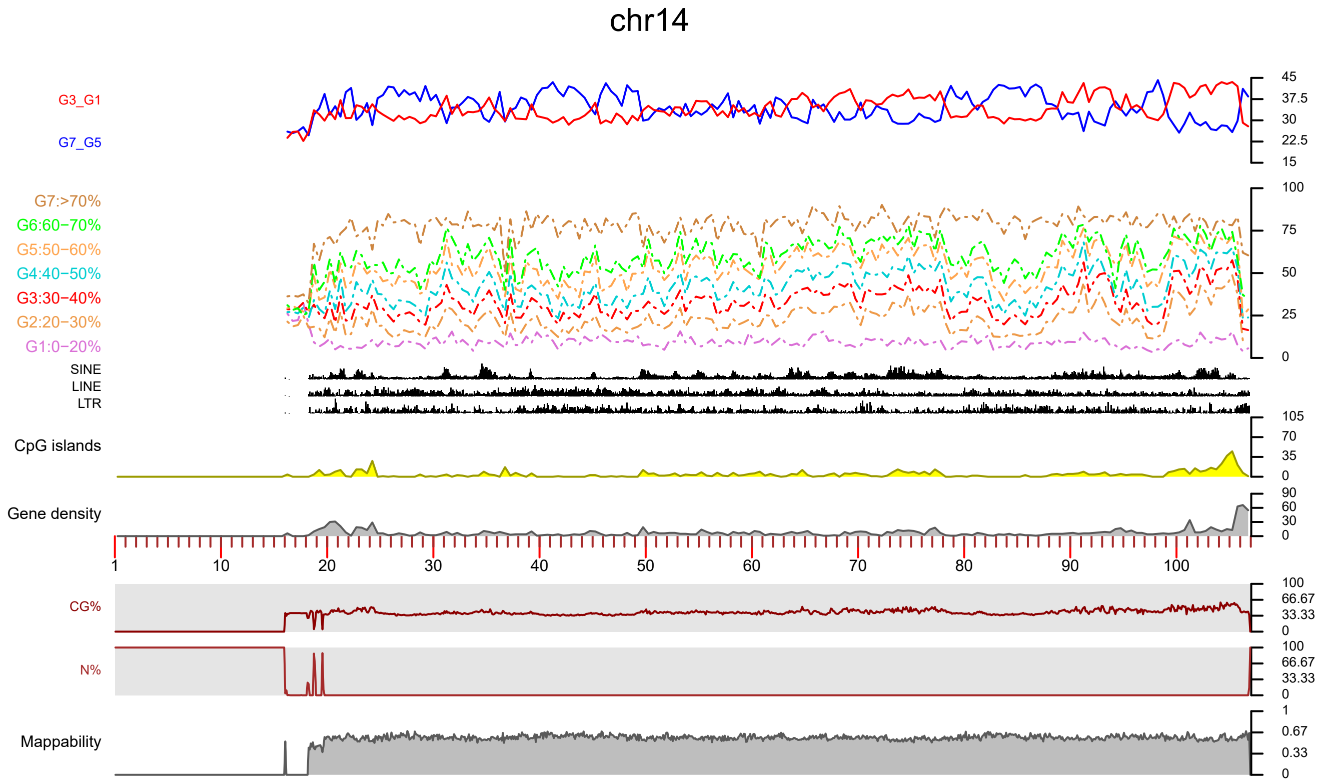
chr14
G3_G1
G7_G5
45
37.5
30
22.5
15
G7:>70%
G6:60–70%
G5:50–60%
G4:40–50%
G3:30–40%
G2:20–30%
G1:0–20%
100
75
50
25
0
SINE
LINE
LTR
CpG islands
105
70
35
0
Gene density
90
60
30
0
1
10
20
30
40
50
60
70
80
90
100
CG%
100
66.67
33.33
0
N%
100
66.67
33.33
0
Mappability
1
0.67
0.33
0

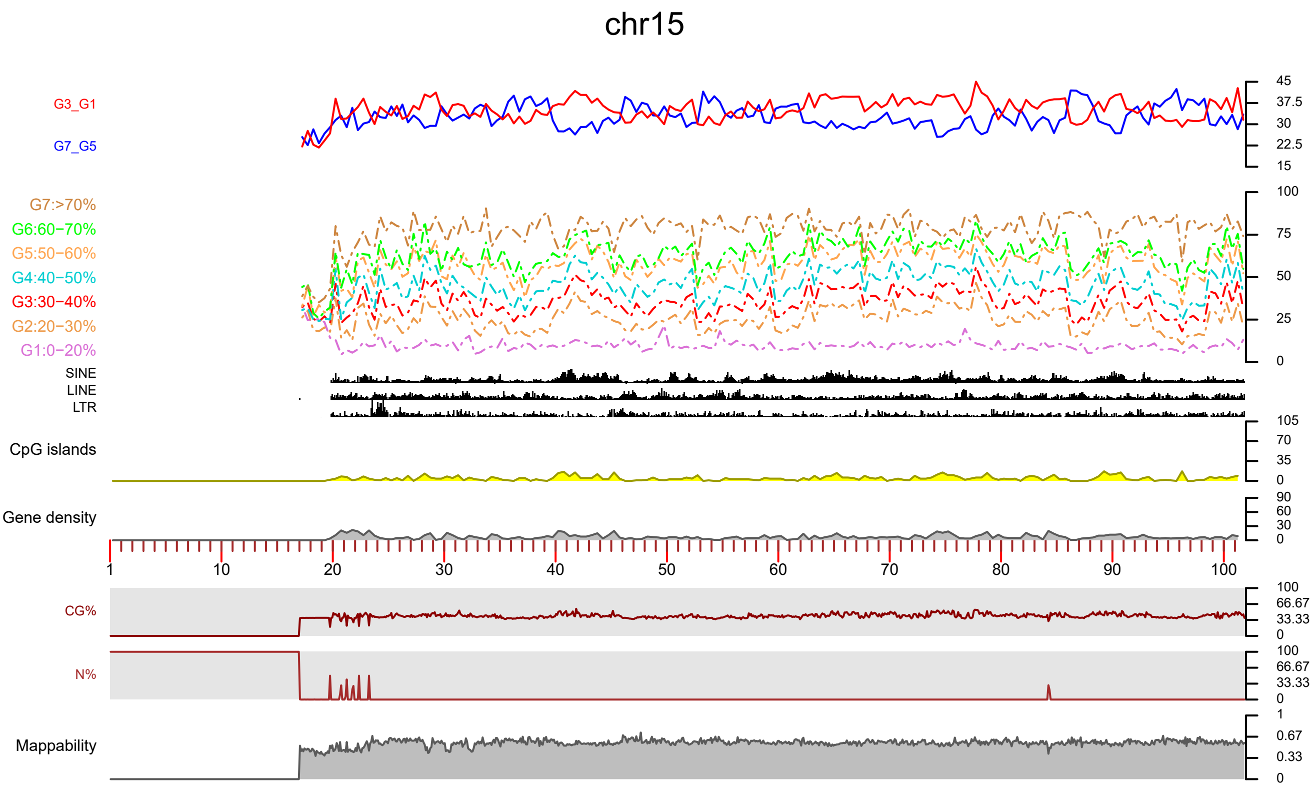
chr15
G3_G1
G7_G5
G7:>70%
G6:60−70%
G5:50−60%
G4:40−50%
G3:30−40%
G2:20−30%
G1:0−20%
SINE
LINE
LTR
CpG islands
Gene density
CG%
N%
Mappability
45
37.5
30
22.5
15
100
75
50
25
0
105
70
35
90
60
1
10
20
30
40
50
60
70
80
90
100
66.67
33.33
0.67
0.33

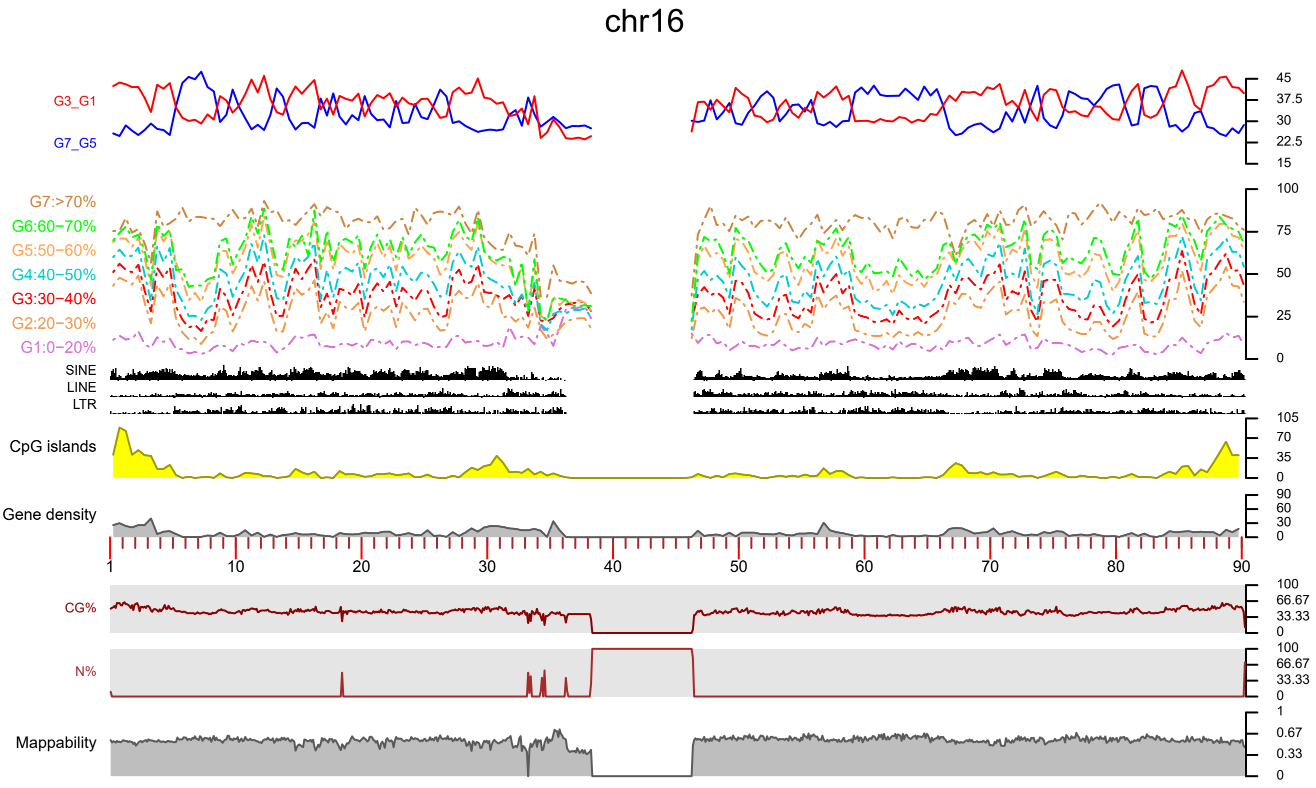
chr16
G3_G1
G7_G5
G7:>70%
G6:60−70%
G5:50−60%
G4:40−50%
G3:30−40%
G2:20−30%
G1:0−20%
SINE
LINE
LTR
CpG islands
Gene density
CG%
N%
Mappability

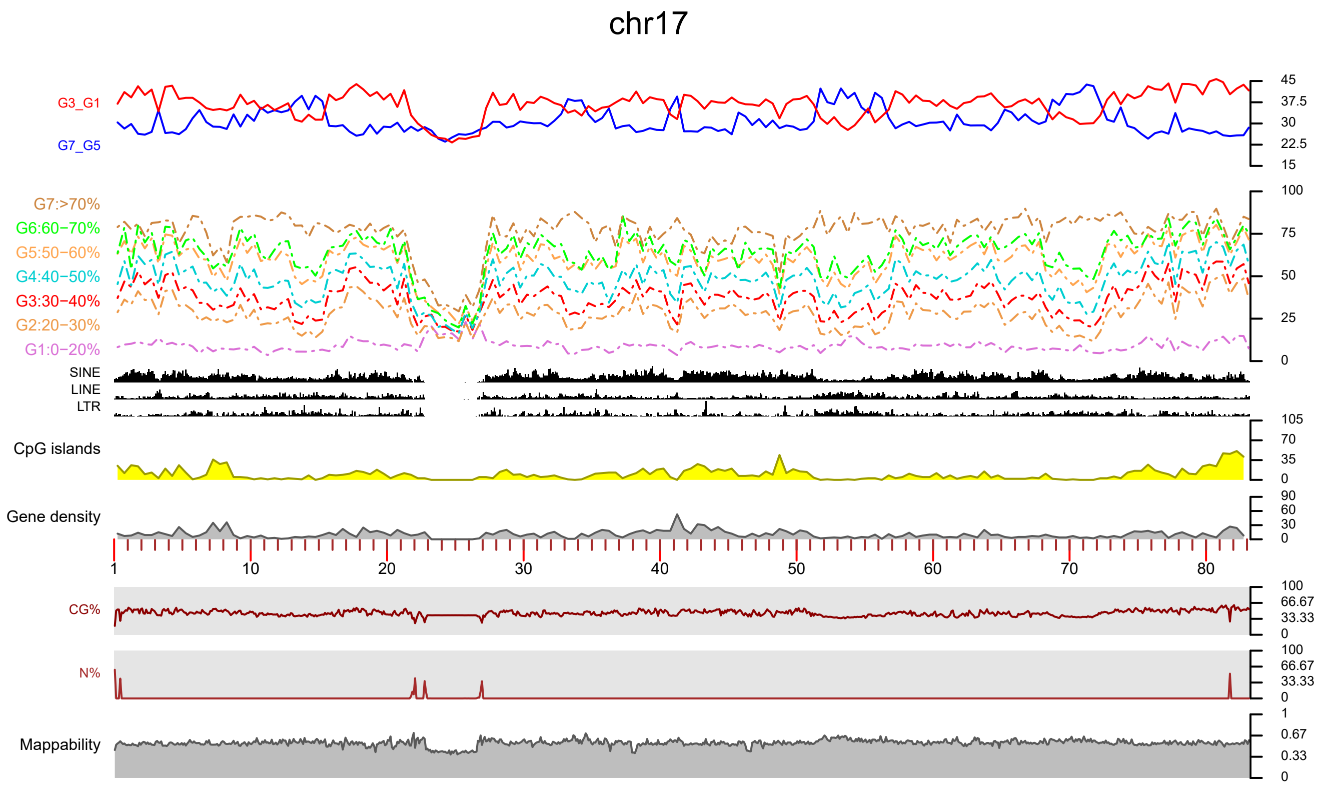
chr17
G3_G1
G7_G5
G7:>70%
G6:60−70%
G5:50−60%
G4:40−50%
G3:30−40%
G2:20−30%
G1:0−20%
SINE
LINE
LTR
CpG islands
Gene density
CG%
N%
Mappability

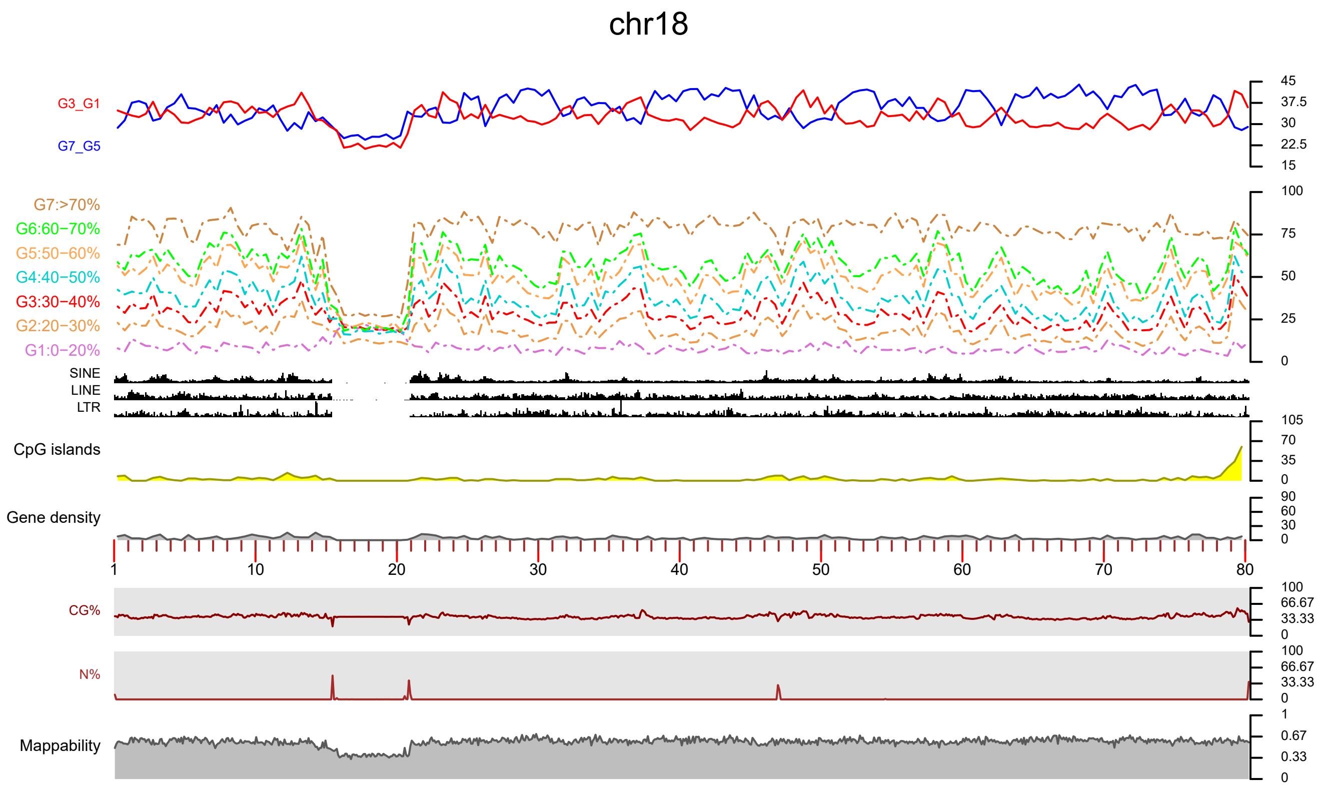
chr18
G3_G1
G7_G5
G7:>70%
G6:60−70%
G5:50−60%
G4:40−50%
G3:30−40%
G2:20−30%
G1:0−20%
SINE
LINE
LTR
CpG islands
Gene density
CG%
N%
Mappability

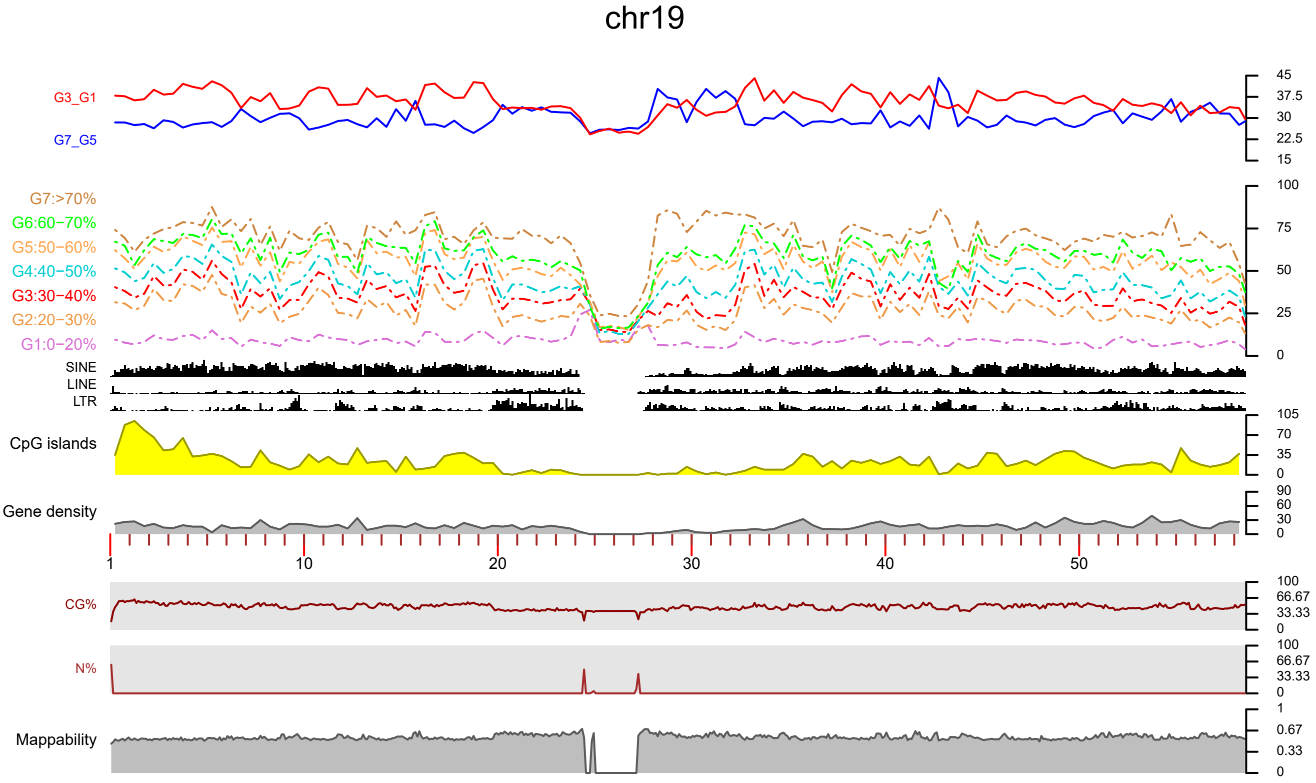
chr19
G3_G1
G7_G5
G7:>70%
G6:60−70%
G5:50−60%
G4:40−50%
G3:30−40%
G2:20−30%
G1:0−20%
SINE
LINE
LTR
CpG islands
Gene density
CG%
N%
Mappability

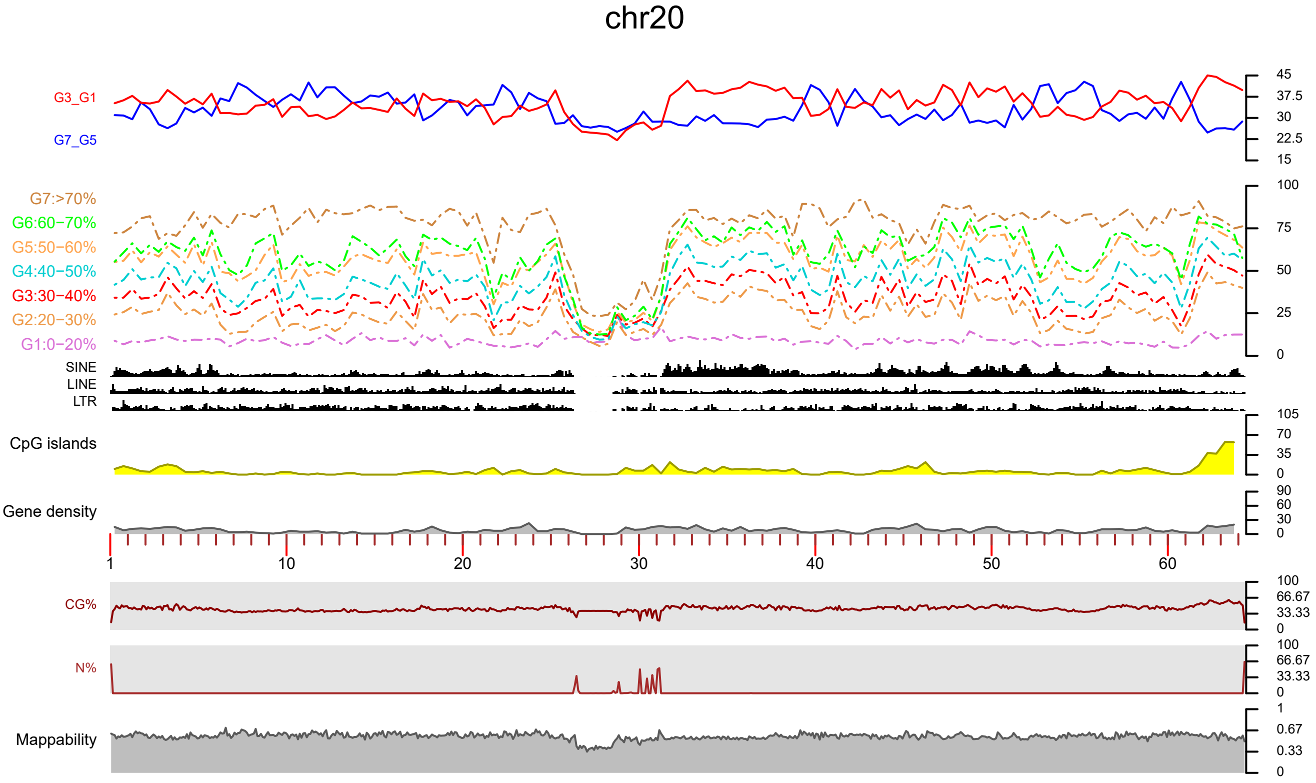
chr20
G3_G1
G7_G5
G7:>70%
G6:60−70%
G5:50−60%
G4:40−50%
G3:30−40%
G2:20−30%
G1:0−20%
SINE
LINE
LTR
CpG islands
Gene density
CG%
N%
Mappability

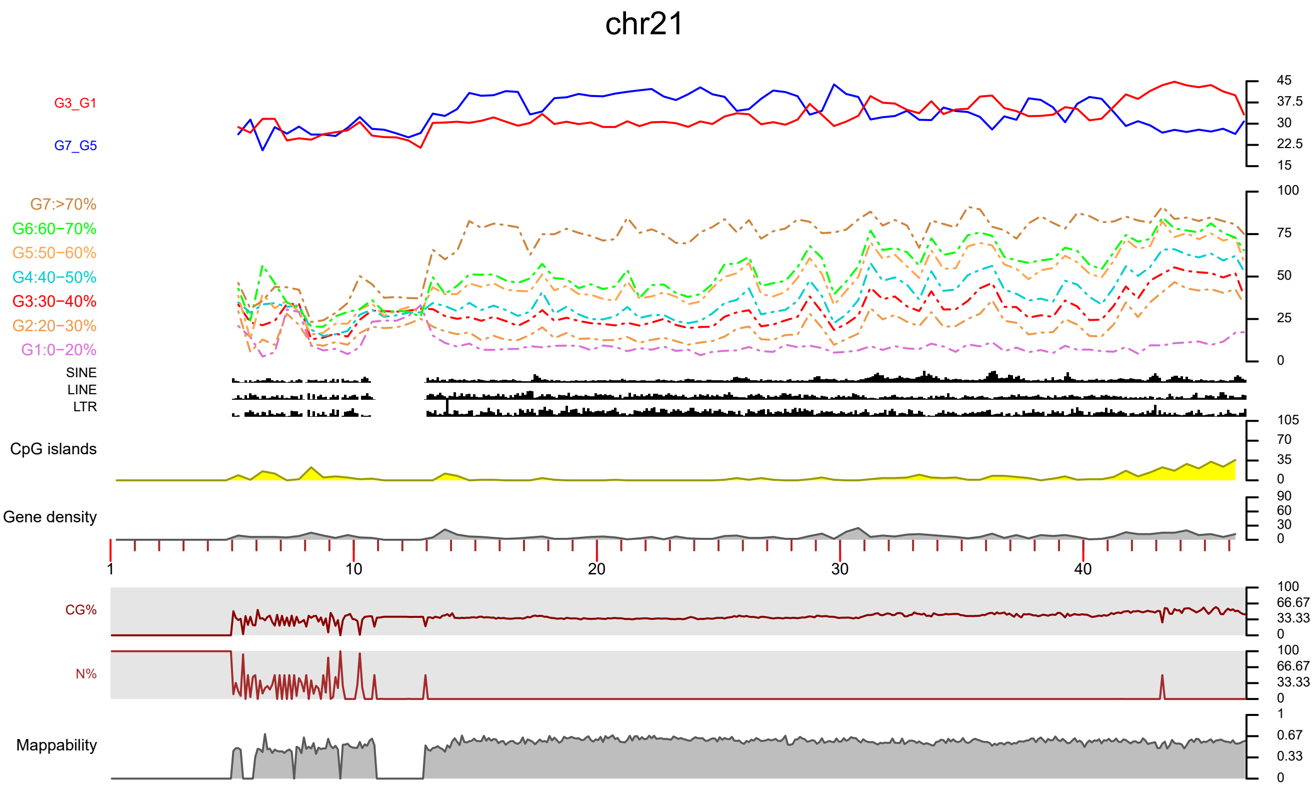
chr21
G3_G1
G7_G5
G7:>70%
G6:60−70%
G5:50−60%
G4:40−50%
G3:30−40%
G2:20−30%
G1:0−20%
SINE
LINE
LTR
CpG islands
Gene density
CG%
N%
Mappability
45
37.5
30
22.5
15
100
75
50
25
0
105
70
35
0
90
60
30
0
1
10
20
30
40
100
66.67
33.33
0
100
66.67
33.33
0
1
0.67
0.33
0

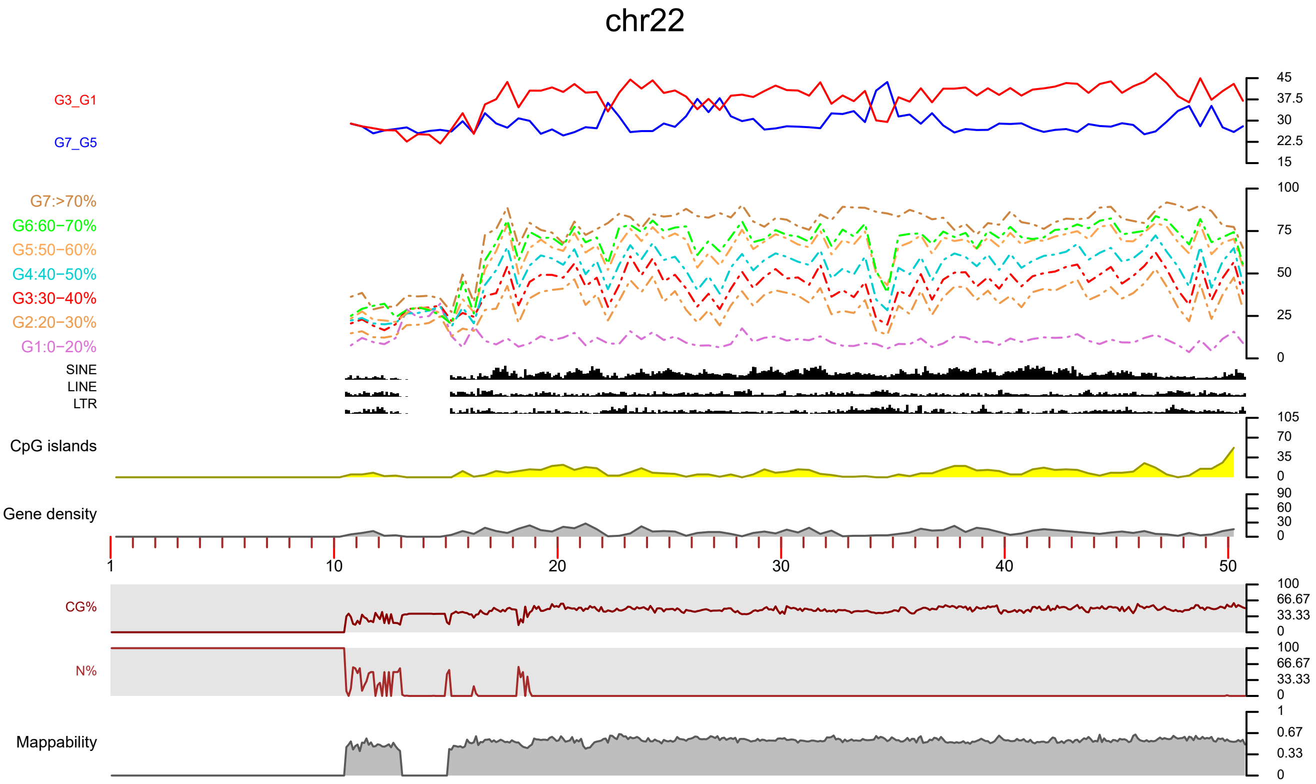
chr22
G3_G1
G7_G5
G7:>70%
G6:60−70%
G5:50−60%
G4:40−50%
G3:30−40%
G2:20−30%
G1:0−20%
SINE
LINE
LTR
CpG islands
Gene density
CG%
N%
Mappability
45
37.5
30
22.5
15
100
75
50
25
0
105
70
35
0
90
60
30
0
1
10
20
30
40
50
100
66.67
33.33
0
100
66.67
33.33
0
1
0.67
0.33
0

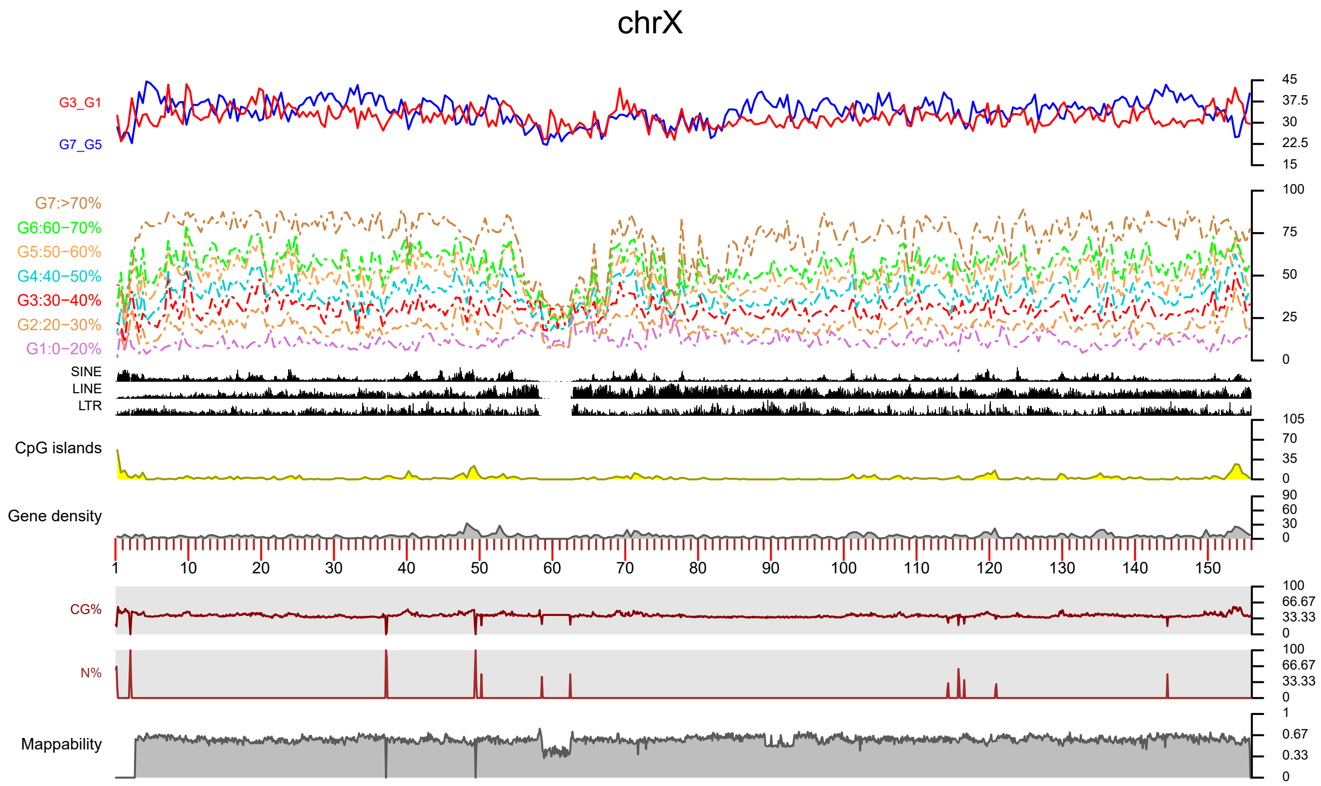
chrX
G3_G1
G7_G5
G7:>70%
G6:60−70%
G5:50−60%
G4:40−50%
G3:30−40%
G2:20−30%
G1:0−20%
SINE
LINE
LTR
CpG islands
Gene density
CG%
N%
Mappability
45
37.5
30
22.5
15
100
75
50
25
0
105
70
35
0
90
60
30
0
1
10
20
30
40
50
60
70
80
90
100
110
120
130
140
150
100
66.67
33.33
0
100
66.67
33.33
0
1
0.67
0.33
0

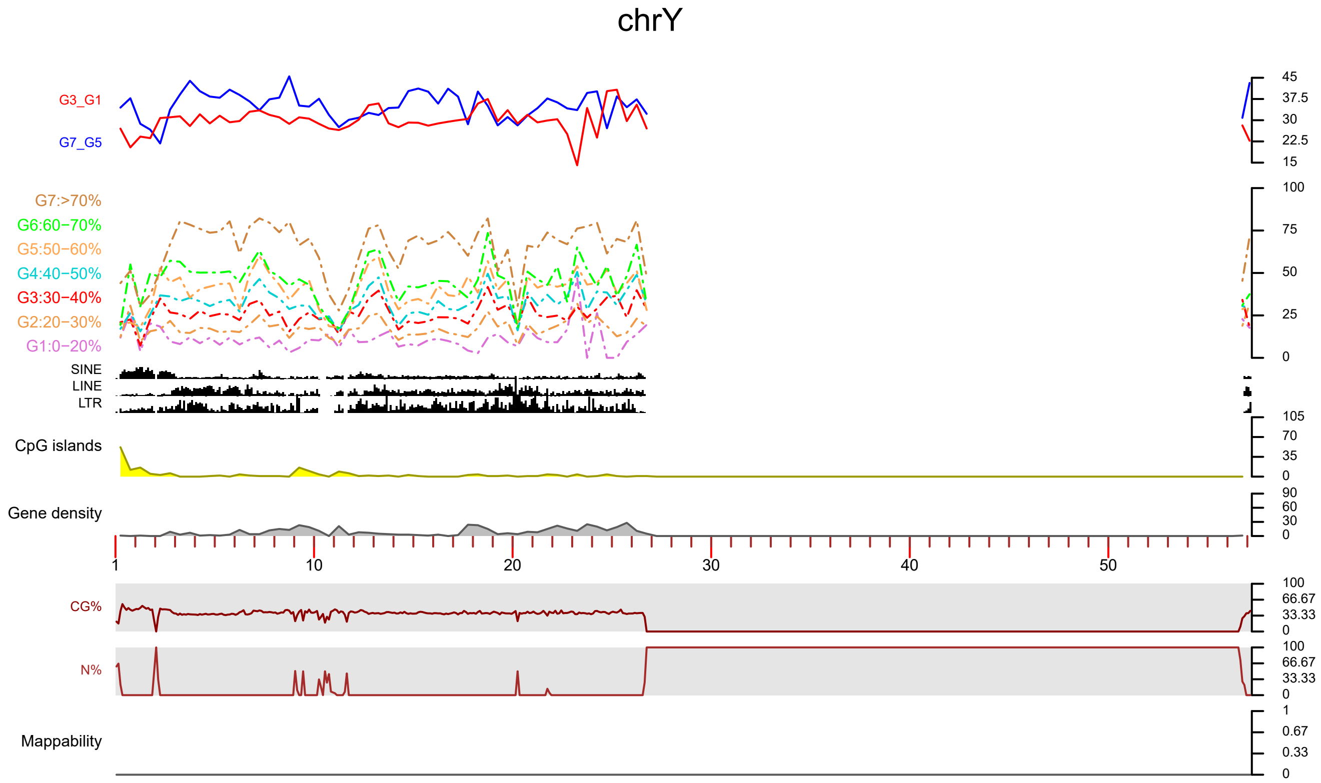
chrY
G3_G1
G7_G5
G7:>70%
G6:60−70%
G5:50−60%
G4:40−50%
G3:30−40%
G2:20−30%
G1:0−20%
SINE
LINE
LTR
CpG islands
Gene density
CG%
N%
Mappability
45
37.5
30
22.5
15
100
75
50
25
0
105
70
35
0
90
60
30
0
1
10
20
30
40
50
100
66.67
33.33
0
100
66.67
33.33
0
1
0.67
0.33
0

Supplementary Figure 8

A.

Marmoset 7M

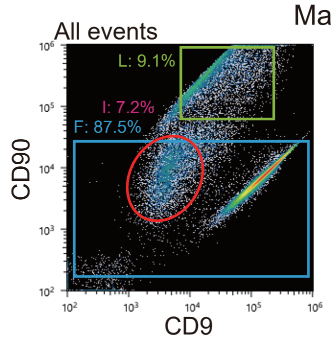

Gate	Germ cell
K	51 / 53
J	14 / 20
L	0 / 30
M	0 / 34

Cynomolgus monkey 1Y8M

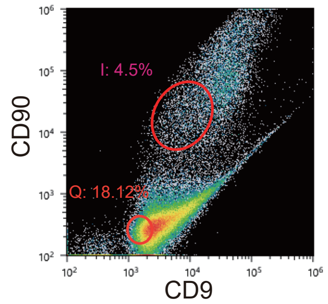

Gate	Germ cell
R	97 / 109
V	0 / 166
Q	0 / 112

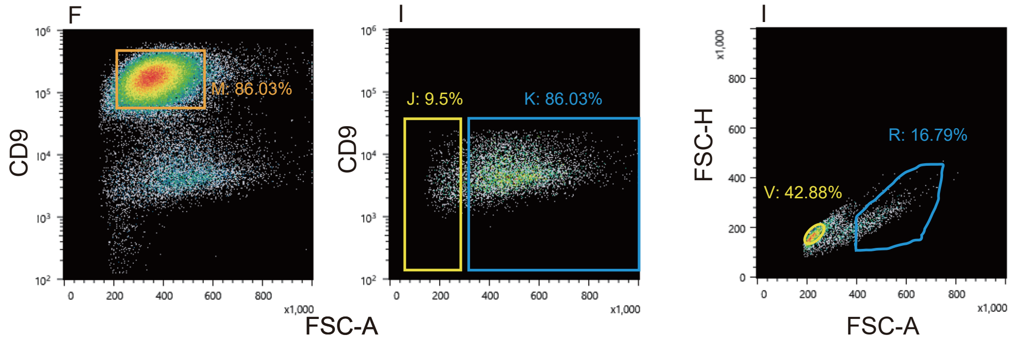

Human 1Y5M

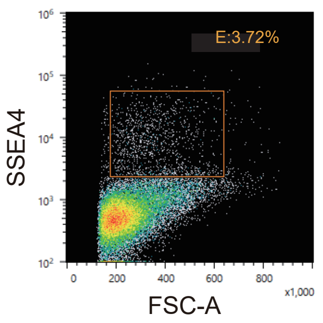

Gate	Germ cell
E	37 / 55

B.

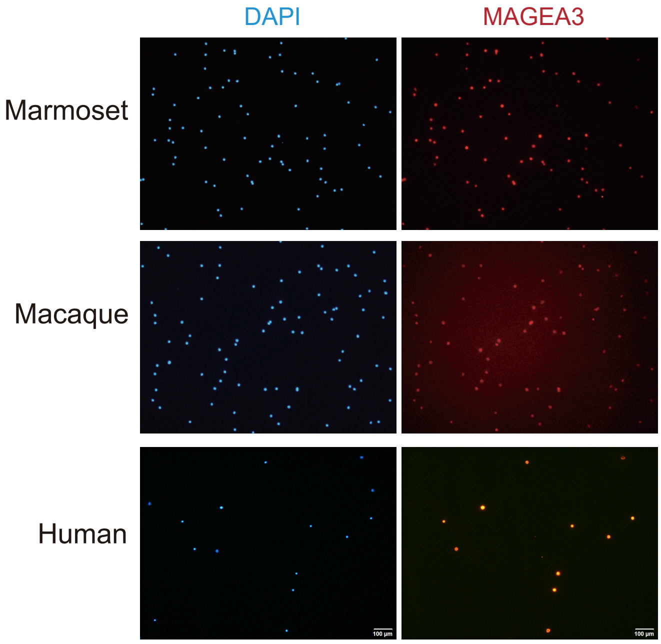

Supplement: Supplementary file 1 — Supplementary Information [file 41467_2026_71405_MOESM1_ESM.pdf]
