# Supplementary material for: Establishment of DNA methylation during primate germ cell development
Source: Nat Commun. 2026 Apr 8;17:4983. doi: 10.1038/s41467-026-71405-z (PMC13237064; doi:10.1038/s41467-026-71405-z)
Supplement: Supplementary file 3 — Reporting Summary [file 41467_2026_71405_MOESM3_ESM.pdf]

nature portfolio

Corresponding author(s): Toshiaki Watanabe

Last updated by author(s): 2026/2/27

Reporting Summary

Nature Portfolio wishes to improve the reproducibility of the work that we publish. This form provides structure for consistency and transparency in reporting. For further information on Nature Portfolio policies, see our Editorial Policies and the Editorial Policy Checklist.

Statistics

For all statistical analyses, confirm that the following items are present in the figure legend, table legend, main text, or Methods section.

n/a	Confirmed	
☐	☒	The exact sample size (*n*) for each experimental group/condition, given as a discrete number and unit of measurement
☐	☒	A statement on whether measurements were taken from distinct samples or whether the same sample was measured repeatedly
☐	☒	The statistical test(s) used AND whether they are one- or two-sided<br>*Only common tests should be described solely by name; describe more complex techniques in the Methods section.*
☒	☐	A description of all covariates tested
☒	☐	A description of any assumptions or corrections, such as tests of normality and adjustment for multiple comparisons
☐	☒	A full description of the statistical parameters including central tendency (e.g. means) or other basic estimates (e.g. regression coefficient) AND variation (e.g. standard deviation) or associated estimates of uncertainty (e.g. confidence intervals)
☐	☒	For null hypothesis testing, the test statistic (e.g. *F*, *t*, *r*) with confidence intervals, effect sizes, degrees of freedom and *P* value noted<br>*Give P values as exact values whenever suitable.*
☒	☐	For Bayesian analysis, information on the choice of priors and Markov chain Monte Carlo settings
☒	☐	For hierarchical and complex designs, identification of the appropriate level for tests and full reporting of outcomes
☒	☐	Estimates of effect sizes (e.g. Cohen's *d*, Pearson's *r*), indicating how they were calculated

Our web collection on statistics for biologists contains articles on many of the points above.

Software and code

Policy information about availability of computer code

Data collection	No software was used
Data analysis	Publicly available software and packages were mainly used to analyze scBS-seq data: for processing BAM files, Samtools version 1.21 (https://doi.org/10.1093/gigascience/giab008); for trimming adapter sequences, Trim Galore version 0.6.10 (https://zenodo.org/records/7598955), Cutadapt version 4.9 (https://doi.org/10.14806/ej.17.1.200), FastQC version 0.12.1; for mapping to the genome, Bismark version 0.24.2 (https://doi.org/10.1093/bioinformatics/btr167), Bowtie2 version 2.5.4 (https://doi.org/10.1109/SFCS.2000.892127); for annotation and analyzing data, bedtools version 2.31.0 (https://doi.org/10.1093/bioinformatics/btq033), Homer version 5.1 (https://doi.org/10.1016/j.molcel.2010.05.004); for displaying data on chromosomes, karyoploteR version 1.32.0 (https://doi.org/10.1093/bioinformatics/btx346), bismap version 1.2.1 (https://doi.org/10.1093/nar/gky677). Seurat version 5.1.0 (https://doi.org/10.1038/s41587-023-01767-y) and Cell Ranger were used to analyze scRNA-seq data. Cell Ranger and ArchR were used to analyze scATAC-seq data. The R and shell scripts used in the ATAC analyses can be obtained from GitHub (https://github.com/Hattyoriiiiii/scatac-caljac-watanabe).

For manuscripts utilizing custom algorithms or software that are central to the research but not yet described in published literature, software must be made available to editors and reviewers. We strongly encourage code deposition in a community repository (e.g. GitHub). See the Nature Portfolio guidelines for submitting code & software for further information.

Data

Policy information about availability of data

All manuscripts must include a data availability statement. This statement should provide the following information, where applicable:

- Accession codes, unique identifiers, or web links for publicly available datasets
- A description of any restrictions on data availability
- For clinical datasets or third party data, please ensure that the statement adheres to our policy

Accession codes for sequencing data are provided.

Research involving human participants, their data, or biological material

Policy information about studies with human participants or human data. See also policy information about sex, gender (identity/presentation), and sexual orientation and race, ethnicity and racism.

Reporting on sex and gender	Testes and ovaries were provided by male and female donors, respectively.
Reporting on race, ethnicity, or other socially relevant groupings	Samples were collected in Japanese and U.S. institutes. No information on race and ethnicity is available.
Population characteristics	Ovaries were excised from four uterine cancer patients of ages 26, 29, 36, and 38 years. Adult testicular tissue is from a 29-year-old patient with obstructive azoospermia. Infant testicular tissues were from teratoma patients (4 samples, 0-1 year-old), biopsy for fertility preservation (2 samples, 0-year-old), and biopsy of normal testis (1 sample, 8-year-old).
Recruitment	Ovary samples used for the analyses were selected based on the presence of oocytes in the sections. Testicular samples from the teratoma patients were from the excised testis containing tumor tissues. In these patients, the entire testis was removed due to difficulty enucleating the tumor tissue. Biopsies from fertility preservation were derived from patients (severe combined immunodeficiency and medulloblastoma) before undergoing anti-cancer therapy.
Ethics oversight	The collection of samples and the use of stored samples at NCCHD, Jichi Medical University, Toho University, and Niigata University were approved by the ethics committees of NCCHD (2022-138 and 2024-044). The study at UCSF was approved by the UCSF ethics committee (#23-39078).

Note that full information on the approval of the study protocol must also be provided in the manuscript.

Field-specific reporting

Please select the one below that is the best fit for your research. If you are not sure, read the appropriate sections before making your selection.

☒ Life sciences ☐ Behavioural & social sciences ☐ Ecological, evolutionary & environmental sciences

For a reference copy of the document with all sections, see nature.com/documents/nr-reporting-summary-flat.pdf

Life sciences study design

All studies must disclose on these points even when the disclosure is negative.

Sample size	The sample size of our single cell DNA methylation analyses is enough to reflect the actual DNA methylation dynamic in vivo. This is confirmed by the linear increase in the methylation levels along developmental stages.
Data exclusions	We included the outlier data.
Replication	The different experiments (immunohistochemistry and sequencing) with different samples were performed to confirm the findings.
Randomization	We allocated randomly.
Blinding	We did not perform the blinding, because blinding does not improve the quality of the data.

Reporting for specific materials, systems and methods

We require information from authors about some types of materials, experimental systems and methods used in many studies. Here, indicate whether each material, system or method listed is relevant to your study. If you are not sure if a list item applies to your research, read the appropriate section before selecting a response.

Materials & experimental systems

n/a	Involved in the study	
☐	☒	Antibodies
☒	☐	Eukaryotic cell lines
☒	☐	Palaeontology and archaeology
☐	☒	Animals and other organisms
☒	☐	Clinical data
☒	☐	Dual use research of concern
☒	☐	Plants

Methods

n/a	Involved in the study	
☒	☐	ChIP-seq
☐	☒	Flow cytometry
☒	☐	MRI-based neuroimaging

Antibodies

Antibodies used	Vasa antibody: AF2030, R&D 1/500<br>MageA3/4 antibody (MABC1150, Merck) 1/500<br>5mC antibody (ab10805, Abcam) 1/50<br>CD9-FITC (MCA469FT, BioRad) 1/50<br>CD90-PE (555596, BD) 1/100<br>DNMT3A (ab188470, Abcam) 1/500<br>DNMT3B (NB300-516-0.025 mg, Novus) 1/200<br>DNMT3L (ab194094, Abcam) 1/150
Validation	All antibodies listed are generally used, and validated.

Animals and other research organisms

Policy information about studies involving animals; ARRIVE guidelines recommended for reporting animal research, and Sex and Gender in Research

Laboratory animals	Marmosets (CLEA), Cynomolgus monkeys (Tsukuba primate center)
Wild animals	*Provide details on animals observed in or captured in the field; report species and age where possible. Describe how animals were caught and transported and what happened to captive animals after the study (if killed, explain why and describe method; if released, say where and when) OR state that the study did not involve wild animals.*
Reporting on sex	Analyses were performed in both ovaries and testes.
Field-collected samples	*For laboratory work with field-collected samples, describe all relevant parameters such as housing, maintenance, temperature, photoperiod and end-of-experiment protocol OR state that the study did not involve samples collected from the field.*
Ethics oversight	Sampling and experiments in this study were approved by the animal ethics committees of the National Center for Child Health and Development (NCCHD), CLEA Japan, Central Institute of Experimental Animals, and Tsukuba Primate Research Center (NIBIOHN).

Note that full information on the approval of the study protocol must also be provided in the manuscript.

Plants

Seed stocks	*Report on the source of all seed stocks or other plant material used. If applicable, state the seed stock centre and catalogue number. If plant specimens were collected from the field, describe the collection location, date and sampling procedures.*
Novel plant genotypes	*Describe the methods by which all novel plant genotypes were produced. This includes those generated by transgenic approaches, gene editing, chemical/radiation-based mutagenesis and hybridization. For transgenic lines, describe the transformation method, the number of independent lines analyzed and the generation upon which experiments were performed. For gene-edited lines, describe the editor used, the endogenous sequence targeted for editing, the targeting guide RNA sequence (if applicable) and how the editor was applied.*
Authentication	*Describe any authentication procedures for each seed stock used or novel genotype generated. Describe any experiments used to assess the effect of a mutation and, where applicable, how potential secondary effects (e.g. second site T-DNA insertions, mosiacism, off-target gene editing) were examined.*

Flow Cytometry

Plots

Confirm that:

☒ The axis labels state the marker and fluorochrome used (e.g. CD4-FITC).

☒ The axis scales are clearly visible. Include numbers along axes only for bottom left plot of group (a 'group' is an analysis of identical markers).

☒ All plots are contour plots with outliers or pseudocolor plots.

☒ A numerical value for number of cells or percentage (with statistics) is provided.

Methodology

Sample preparation	Monkey (Human) testes were dissociated using 1 mg/mL collagenase type I (type IV) in DMEM at 37°C with shaking for 30 min. After centrifugation (200 g, 5 min), the samples were digested with 0.25% trypsin-EDTA at 37C for 10 min. Reactions were halted by adding an equal volume of 10% FCS/DMEM. DNase I was added to a final concentration of 25 µg/mL, and the cells were incubated at room temperature for 1 min. After filtering through a 60-µm strainer, cells were centrifuged, and supernatants were discarded. The washing process was repeated twice using 10% FCS/DMEM. Collagenase treatment was omitted for testes from younger animals (<6 months old), with only trypsin treatment applied.
Instrument	SH800
Software	N/A
Cell population abundance	Percentage of cell population is described in Supplementary Figure
Gating strategy	Described in Supplementary information

☒ Tick this box to confirm that a figure exemplifying the gating strategy is provided in the Supplementary Information.
