# Supplementary material for: Establishment of DNA methylation during primate germ cell development
Source: Nat Commun. 2026 Apr 8;17:4983. doi: 10.1038/s41467-026-71405-z (PMC13237064; doi:10.1038/s41467-026-71405-z)
Supplement: Supplementary file 4 — Description of Additional Supplementary Files [file 41467_2026_71405_MOESM4_ESM.docx]

**Description of Additional Supplementary Files**

Supplementary Data 1

Description: Information on animal samples used for this study

Supplementary Data 2

Catalog of testis germ cells analyzed in this study

Supplementary Data 3

Marmoset scBS-seq and scRNA-seq datasets used in this study

Supplementary Data 4

Coordinates of the imprinted loci used for removing somatic cells

Supplementary Data 5

scBS-seq data from Cynomolgus monkeys

Supplementary Data 6

Human samples used in this study

Supplementary Data 7

Human scBS-seq data
